# *Indigofera suffruticosa* aerial parts extract induce G2/M arrest and ATR/CHK1 pathway in Jurkat cells

**DOI:** 10.1186/s12906-023-04325-w

**Published:** 2024-01-09

**Authors:** Hong-Loan Tran, Kuei-Hung Lai, Hsun-Shuo Chang, Yi-Siao Chen, Hui-Chun Wang, Shuen-Shin Yang, Hsueh-Wei Chang, Chin-Mu Hsu, Chia-Hung Yen, Hui-Hua Hsiao

**Affiliations:** 1 Graduate Institute of Natural Products, College of Pharmacy, Kaohsiung Medical University, Kaohsiung, 80708 Taiwan; 2 PhD Program in Clinical Drug Development of Herbal Medicine, College of Pharmacy, Taipei Medical University, Taipei, 11031 Taiwan; 3 Graduate Institute of Pharmacognosy, College of Pharmacy, Taipei Medical University, Taipei, 11031 Taiwan; 4 Drug Development and Value Creation Research Center, Kaohsiung Medical University, Kaohsiung, 80708 Taiwan; 5 School of Pharmacy, College of Pharmacy, Kaohsiung Medical University, Kaohsiung, 80708 Taiwan; 6 Ph.D. Program in Environmental and Occupational Medicine, College of Medicine, Kaohsiung Medical University and National Health Research Institutes, Kaohsiung, 80708 Taiwan; 7 Department of Biomedical Science and Environmental Biology, College of Life Science, Kaohsiung Medical University, Kaohsiung, 80708 Taiwan; 8 Department of Medical Research, Kaohsiung Medical University Hospital, Kaohsiung, 80708 Taiwan; 9 Division of Hematology and Oncology, Department of Internal Medicine, Kaohsiung Medical University Hospital, Kaohsiung, 80708 Taiwan; 10 Center for Liquid Biopsy and Cohort Research, Kaohsiung Medical University, Kaohsiung, 80708 Taiwan; 11 Faculty of Medicine, Kaohsiung Medical University, Kaohsiung, 80708 Taiwan; 12 Center for Cancer Research, Kaohsiung Medical University, Kaohsiung, 80708 Taiwan; 13 Cancer Center, Kaohsiung Medical University Hospital, Kaohsiung, 80708 Taiwan

**Keywords:** Folk medicine, *Indigofera suffruticosa*, Acute lymphoblastic leukemia

## Abstract

**Background:**

*Indigofera suffruticosa* Mill. is used as a folk medicine for treating patients with leukemia, however very little is known regarding the molecular mechanism of its anti-leukemic activity and the chemical profile of the active extract. The present study aimed to reveal the molecular effect of *I. suffruticosa* aerial parts extract (ISAE) on leukemia cells and its chemical constituents.

**Methods:**

Cytotoxicity of ISAE were determined by resazurin viability assay, multitox – Glo multiplex cytotoxicity assay, and Annexin V staining assay. Cell cycle profiles were revealed by propidium iodide staining assay. The effects of ISAE on G2/M arrest signaling and DNA damage were evaluated by Western blot assay and phospho-H2A.X staining assay. The chemical profile of ISAE were determined by tandem mass spectroscopy and molecular networking approach.

**Results:**

We showed that the acute lymphoblastic leukemia cell line Jurkat cell was more responsive to ISAE treatment than other leukemia cell lines. In contrast, ISAE did not induce cytotoxic effects in normal fibroblast cells. Cell cycle analysis revealed that ISAE triggered G2/M arrest in Jurkat cells in dose- and time-dependent manners. Elevation of annexin V-stained cells and caspase 3/7 activity suggested ISAE-induced apoptosis. Furthermore, ISAE alone could increase the phosphorylation of CDK1 at Y15 and activate the ATR/CHK1/Wee1/CDC25C signaling pathway. However, the addition of caffeine, a widely used ATR inhibitor to ISAE, reduced the phosphorylation of ATR, CHK1, and CDK1, as well as G2/M arrest in Jurkat cells. Moreover, increased phospho-H2A.X stained cells indicated the involvement of DNA damage in the anti-leukemic effect of ISAE. Finally, qualitative analysis using UPLC-tandem mass spectroscopy and molecular networking revealed that tryptanthrin was the most abundant organoheterocyclic metabolite in ISAE. At equivalent concentrations to ISAE, tryptanthrin induced G2/M arrest of Jurkat cells, which can be prevented by caffeine.

**Conclusions:**

ISAE causes G2/M arrest via activating ATR/CHK1/CDK1 pathway and tryptanthrin is one of the active components of ISAE. Our findings provide subtle support to the traditional use of *I. suffruitcosa* in leukemia management in folk medicine.

**Supplementary Information:**

The online version contains supplementary material available at 10.1186/s12906-023-04325-w.

## Background

Acute lymphoblastic leukemia (ALL) is a type of cancer that originates from the uncontrolled growth of immature lymphoid cells. These cells, stalled in their early stages of differentiation, can rapidly invade and affect vital sites such as the bone marrow, blood, and various extramedullary locations [1]. Globally, the incidence of ALL varies but sits between 1.1 to 2.1 cases per 100,000 individuals each year [2]. The incidence of ALL isn’t consistent across all age groups. It demonstrates a bimodal distribution, with incidence peaks observed around the ages of 5 and then again in the 50s [3]. A significant observation to note is that almost 40% of the total annual cases of ALL are diagnosed in adults. In pediatric patients, ALL can be treated successfully in more than 90% of cases. However, the scenario is less optimistic for adults. The available treatment options have often been less effective, leading to cure rates of under 40% [4]. This underscores the need for new therapeutic developments for ALL.

Natural products are metabolites that are produced by plants and microbes and are a major source of new drugs, adjuvant agents, or supplements [5, 6]. *Indigofera suffruticosa* Mill has been reported to show several biological activities. *I. suffruticosa* belongs to the Fabaceae family. This plant, known as a natural indigo dyer, is distributed in tropical and subtropical countries [7]. Several pharmacological activities of *I. suffruticosa* have been reported, including anti-cancer activity against sarcoma and cervical, breast, and lung cancers, embryotoxic activity, anti-inflammatory activity, anti-microbial activity, anti-oxidant activity, chemopreventive activity, hepatoprotective activity, and immunostimulatory activities (as reviewed in [7]).

*I. suffruticosa* has been used in Taiwanese folk medicine to treat cirrhosis, fever, and inflammation [8]. The traditional application of *I. suffruticosa* as a folk remedy has been inherited from the native region of one of the authors. In certain areas, a decoction prepared from the aerial parts of *I. suffruticosa* is used for leukemia treatment, though these practices are not documented in published literature. Details regarding the length of its usage, the degree of its adoption among local populations, and specific therapeutic results remain largely undocumented and difficult to ascertain. Presently, there exists no scientific evidence in the form of clinical or laboratory data to substantiate these claims. The specific anti-leukemic properties of *I. suffruticosa* remain largely unexplored. Therefore, the objectives of this study were to evaluate the anti-leukemic effects and to reveal the mechanism of action of *I. suffruticosa* extract, as well as to identify its potential active component(s).

## Methods

### The preparation of *I. suffruticosa* Mill. extract (ISAE)

The aerial parts of *I. suffruticosa* Mill. were collected from the Siaying District, Tainan, Taiwan, in July 2015. The plant material was taxonomically identified by Professor Hsun-Shou Chang of the School of Pharmacy, Kaohsiung Medical University. A voucher specimen (Chen 5673) was deposited in the herbarium of the College of Pharmacy, Kaohsiung Medical University, Kaohsiung, Taiwan. The aerial parts of *I. suffruticosa* were extracted with methanol:water (1:1) for three days at room temperature. The methanol-water extracted solution was collected and concentrated using a rotary evaporator under vacuum. Residual water and solvent were removed in a vacuum oven, after which the extract was stored at 4 °C. The *I. suffruticosa* aerial parts extract (ISAE) used in this study was prepared by dissolving 100 mg of the crude methanol-water extract in 1 mL of distilled water with 30 min of sonication, followed by the removal of the insoluble pellets by centrifugation. ISAE was sterilized by filtration with 0.2 μm syringe filter (Pall Corporation, USA) and then used for cell-based assays. To determine the concentration of ISAE, 1 mL of ISAE was dried under vacuum in a rotary evaporator and the weight of the resultant powder was measured to calculate the concentration of ISAE. The concentration of the ISAE stock solution was 29.2 mg/mL.

### Cell culture

Leukemia cells, including Jurkat (BCRC60424, BCRC, Hsinchu, Taiwan), HL-60 (BCRC60027, BCRC), and KG-1 (BCRC60158, BCRC), were cultured in complete Roswell Park Memorial Institute-1640 medium (Thermo Fisher Scientific, Waltham, MA, USA) supplemented with 10% heat inactivated fetal bovine serum (FBS, Thermo Fisher Scientific), penicillin (100 U/mL, Thermo Fisher Scientific), nonessential amino acids (0.1 mM, Thermo Fisher Scientific), and L-glutamine (2 mM, Thermo Fisher Scientific). Human dermal fibroblast cells Ccd-966sk (BCRC60153, BCRC) were cultured in complete Dulbecco’s modified Eagle’s medium (Thermo Fisher Scientific) supplemented with 10% heat inactivated FBS, penicillin (100 U/mL), nonessential amino acids (0.1 mM), and L-glutamine (2 mM). The passage number used when performing experiments ranged from 5th to 15th passages.

### Cell viability assay

10,000 cells/well were seeded in a 96-well plate containing 100 µL of culture medium/well in triplicate and treated with ISAE or the test compounds at the indicated concentrations for 72 h. At the assay time point, resazurin (Cayman Chemical, Ann Arbor, MI, USA) was added at a final concentration of 0.1 mg/mL and further incubated for 4 h at 37 °C. After incubation, resazurin fluorescence (ex/em: 530 nm/590 nm) was measured from the culture supernatant using a Synergy HT Multi-Mode Reader (BioTek Instruments, Winooski, VA, USA) to determine cell viability, and vehicle control was used as 100%.

### Multitox – Glo multiplex cytotoxicity assay

Cells were seeded as described in the cell viability assay and treated with ISAE at the indicated concentrations for 24 h. Then, the MultiTox – Glo Multiplex Cytotoxicity Assay (Promega Corporation, Madison, WI, USA) was used to measure live-cell and dead-cell protease activity according to the manufacturer’s instructions.

### Cell cycle analysis

Cell cycle analysis was performed as previously described [9]. Jurkat cells were treated with ISAE or the test compounds at the indicated concentrations and for the indicated times. Cells were collected, fixed in ice-cold 70% ethanol on ice for a minimum of two hours, then washed and resuspended in 0.5 mL of PBS containing propidium iodide (1 mg/mL, CAS: 25535-16-4, Sigma-Aldrich, St. Louis, Missouri, USA), RNase A (100 µg/mL, BIOTOOLS, New Taipei City, Taiwan), and 0.3% Triton X-100 (CAS: 9002-93-1, VWR Life Science, Radnor, PA, USA). After incubation at 37 °C for 30 min, the cells were analyzed by using a BD LSR II flow cytometer, and the data were analyzed by using FlowJo V10 software. In combination treatment experiments, Jurkat cells were pre-treated with caffeine for 1 h and then treated with ISAE or the test compounds in the presence of caffeine.

### Annexin V staining assay

Annexin V staining assay was performed as previously described [10]. Cells were treated with ISAE at the indicated concentrations for 24 h. Cells were stained with the annexin V-FITC Apoptosis Detection Kit (eBioscience, San Diego, CA, United States) according to the manufacturer’s instructions. The stained cells were then analyzed using a BD LSR II flow cytometer, and the data were analyzed using FlowJo V10 software.

### Western blot assay

Protein extraction and Western blotting were performed as previously described [11]. Information on the primary and secondary antibodies used in this study is provided in Supplementary Table S1. After transferring the membranes, we cut them into long strips corresponding to the molecular weight of each targeted molecule. This is done before incubating them with different primary antibodies, a step that helps conserve the use of antibodies. These sliced membranes are then placed on the development plate for the final exposure. Due to the varying number of samples processed with electrophoresis each time, some of the cut membranes are longer and contain more lanes, while others are shorter. This is hereby clarified.

### Immuno-fluorescence assay

Jurkat cells were treated with ISAE at the indicated concentrations for 24 h. Cells were then collected, washed with ice-cold PBS twice, and fixed in 4% paraformaldehyde for 20 min at 25 °C. Fixed cells were washed and resuspended in deionized water, and then a 10 µL cell suspension containing 5 × 10^4^ cells was spread on a microscope slide. Cell spreads were allowed to dry at room temperature for 30 min. The dried cell slides were used for the immuno-fluorescence assay. The slides were washed with washing buffer (0.1% BSA in PBS) and blocked with blocking buffer (5% BSA, 0.3% Triton X-100 in PBS) for 1 h at 25 °C. The slides were allowed to react with primary antibody (anti-p-H2A.X) at 4°C overnight, and then with tetramethyl rhodamine isothiocyanate-conjugated anti-mouse IgG antibody as secondary antibody for 1 h at room temperature. DNA was stained with Hoechst H33258 (Sigma-Aldrich) to localize the cell nuclei. Images of the nuclei and p-H2A.X were acquired and analyzed automatically using an HCS instrument (ImageXpress Micro System, Molecular Devices, Sunnyvale, CA, USA).

### MS/MS non-targeted fragment ion collection using ultra-performance liquid chromatography quadrupole time-of-flight mass spectrometry (UPLC-QTOF-MS)

The collection of MS/MS (MS^2^) data was conducted on a Waters SYNAPT G2 LC/Q-TOF system (Waters Corporation, Milford, MA, USA) as previously described [12]. Before the MS analysis, chromatographic separation was achieved using a Waters Acquity UPLC BEH C18 column (1.7 μm, 2.1 mm × 100 mm). The mobile phase utilized a gradient sequence of MeCN (A, with 0.1% formic acid) and water (W, with 0.1% formic acid): 0.01 min at 10% A and 30 min at 100% A. The flow was controlled at 0.5 mL/min, while the column’s temperature was consistently held at 40 °C in an oven. For sample preparation, 5 mg of ISAE was dissolved in 1 mL of methanol to achieve a concentration of 5,000 ppm and then passed through a 0.45 μm membrane filter. Each automatic sample injection had a volume of 5 µL. Both MS^1^ and MS^2^ non-targeted data were gathered within the m/z range of 100–2,000. The data-dependent acquisition method was employed for MS^2^ scans, fragmenting five precursor ions with a collision energy gradient from 10 to 50 V. Finally, the Waters MassFragment software (MassLynx4.1, Waters, MA, USA) was used to process the MS data.

### Global Natural products Social (GNPS)-based molecular networking analysis

The GNPS web-based platform (https://gnps.ucsd.edu) was used to analyze and output MS^2^ molecular networking data on October 20th, 2021. The MS^2^ spectra were window filtered according to the top three strongest ion peaks in the ± 50 Da window throughout the spectrum. A network was then created, in which linkages between nodes were filtered by a cosine value above 0.70 and at least four matched peaks. The nodes that appeared in the network were annotated based on experimental MS^2^ fragmentations of the isolates. The molecular network was visualized and laid out using Cytoscape 3.8.2 (Cytoscape 3.8.2, NRNB, CA, USA).

### Relative quantitative analysis using ultra-performance liquid chromatography-tandem mass spectrometry (UPLC-MS/MS)

A Shimazu Nexera X2 UPLC system (Shimazu, Kyoto, Japan) was used for relative quantitative analysis of three commercially available compounds in the ISAE extract. Liquid chromatography was carried out using a Thermo Hypersil GOLD C18 (1.9 μm, 2.1 mm × 100 mm) column (Waltham, MA, USA). The mobile phase was prepared by mixing acetonitrile (A, containing 0.1% formic acid) and water (W, containing 0.1% formic acid), the gradient sequence was executed as follows: 0.01–15 min, 30–100% A. The flow rate was fixed at 0.5 mL/min, the column temperature was maintained at 40 °C. To prepare the sample, 5 mg ISAE and 1 mg of tryptanthrin (CAS:13220-57-0, Selleckchem, Houston, TX, USA), indigo (CAS:482-89-3, Selleckchem), and indirubin (CAS:479-41-4, Selleckchem) were individually dissolved in 1 mL of methanol and filtered through a 0.45 μm membrane filter before loading into the UPLC column. The sample injection was implemented automatically with 1 µL (ISAE) and 0.3 µL (three commercially available compounds) per injection. Selected MS ion scan experiments (in positive mode) were performed using a Shimadzu LCMS-8045 mass spectrometer. The relative abundance of the three selected compounds in the ISAE extract was calculated by comparing the peak area ratios with those of the standard compounds. All acquired MS data were processed using the LCMS LabSolutions software (Version 5.93, Shimazu, Kyoto, Japan).

### Statistical analyses

All data were analyzed using the GraphPad Prism 8.0 software (La Jolla, CA, USA). Statistical analyses were performed using one-way ANOVA with Tukey’s post-hoc test. Statistical significance was set at *p* < 0.05.

## Results

### The *I. suffruticosa* aerial parts extract (ISAE) caused cytotoxic effect in Jurkat cells

**Fig. 1:**
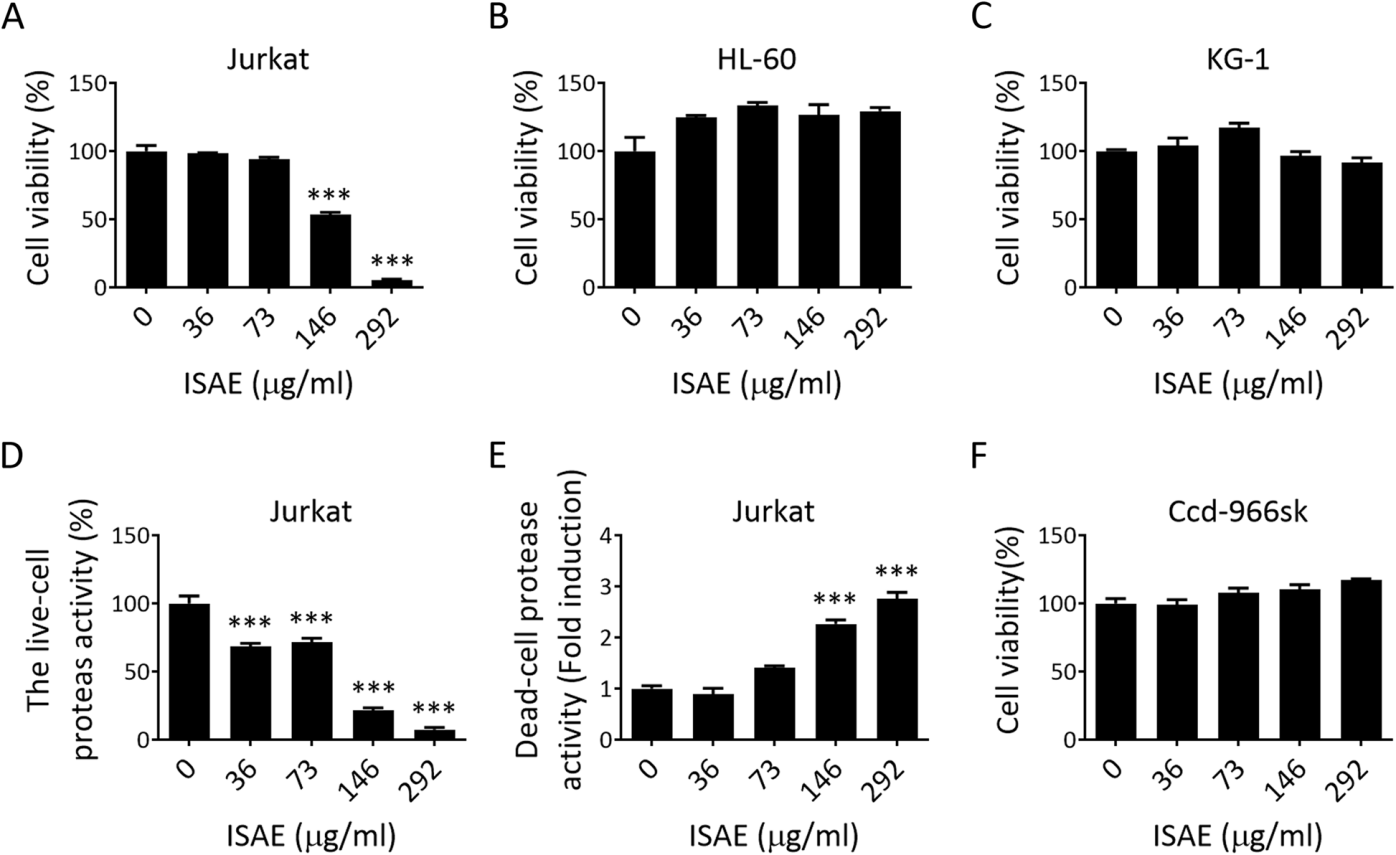
The *I. suffruticosa* aerial parts extract (ISAE) caused cytotoxic effects in Jurkat cells. **A**-**C** Jurkat (**A**), HL-60 (**B**), and KG-1 cells (**C**) were treated with indicated concentrations (36, 73, 146, 292 µg/mL) of ISAE for 72 h. Resazurin reagent was used to determine cell viability. Distilled water was used as solvent control for 100% viability. **D**-**E** The levels of live-cell protease activity (**D**) and dead-cell protease activity (**E**) in Jurkat cells after 24 h of ISAE treatment were measured by the Multitox – Glo multiplex cytotoxicity assay. **F** Human normal dermal fibroblasts (Ccd-966sk) were treated with the indicated concentrations (36, 73, 146, 292 µg/mL) of ISAE for 72 h. Cell viability was measured as described in (**A**). Data are presented as the mean ± SD from three independent experiments. * indicates significant difference from control group (***p < 0.001, one-way ANOVA). ISAE inhibited the viability of Jurkat cells but had no obvious cytotoxic effects on HL-60, KG-1, and Ccd-966sk cells

First, we investigated the potential anti-leukemic effect of the *I. suffruticosa* aerial parts extract (ISAE) on Jurkat (T cell acute lymphoblastic leukemia, T-ALL), HL-60 (acute promyelocytic leukemia, APML), and KG-1 (acute myelocytic leukemia, AML) cells. As shown in Fig. 1A, ISAE inhibited Jurkat cell viability in a concentration-dependent manner, with an IC50 of 155 µg/ml. In contrast, ISAE had no obvious cytotoxic effects on HL-60 or KG-1 cells (Fig. 1B, C). Next, the cytotoxic effect of ISAE on Jurkat cells was confirmed using a Multitox – Glo multiplex cytotoxicity assay. The results showed that ISAE suppressed live-cell protease activity and induced dead-cell protease activity in a concentration-dependent manner (Fig. 1D, E). Furthermore, we evaluated the cytotoxic effect of ISAE on Ccd-996sk cells, a normal human fibroblast cell line derived from normal human skin tissue. As shown in Fig. 1F, no cytotoxic effects were observed in the Ccd-996sk cells treated with ISAE. Taken together, these results suggest that ISAE can induce cytotoxic effects in Jurkat cells; thus, the following experiments were performed using this cell line.

### ISAE induced G2/M arrest and apoptosis in Jurkat cells

**Fig. 2:**
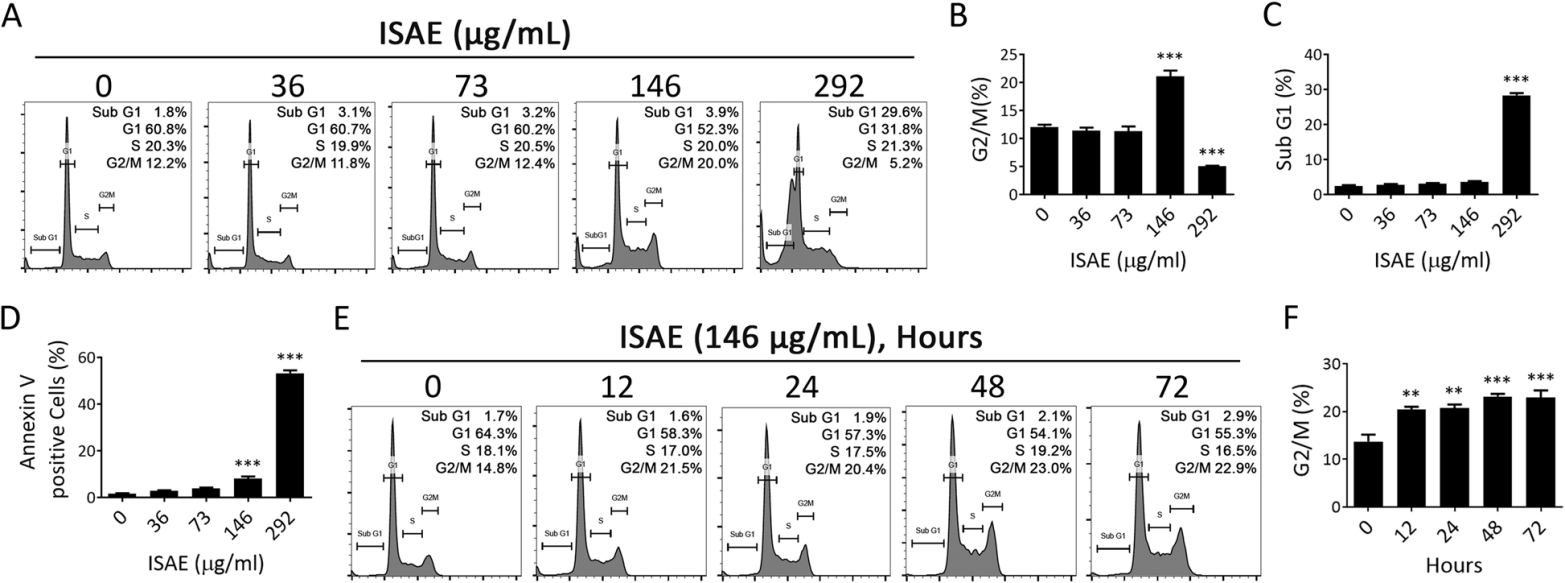
ISAE induced G2/M arrest and apoptosis in Jurkat cells. **A** Jurkat cells were exposed to indicated concentrations (36, 73, 146, 292 µg/mL) of ISAE for 24 h, then fixed for propidium iodide (PI) staining. Cell cycle profiles were recorded by flow cytometry. The Dotplot for cell gating of this experiment were presented in Supplementary Fig. S4**B**-**C** Quantitative results of G2/M phase (**B**) and sub-G1 phase (**C**) of panel (**A**). **D** Jurkat cells were exposed to indicated concentrations (36, 73, 146, 292 µg/mL) of ISAE for 24 h, then harvested for annexin V staining and flow cytometry analysis. Representative histogram was showed in Supplementary Fig. S1. **E** Jurkat cells were treated with 146 µg/ml ISAE for indicated times (12, 24, 48, 72 h). Cell cycle profile were determined as described in (**A**). **F** Quantitative results of G2/M phase of panel (**E**). Data are presented as mean ± SD from three independent experiments. * indicates significant difference from control group (**p < 0.01, ***p < 0.001, one-way ANOVA)

To further investigate the mechanisms underlying this cytotoxic effect, we analyzed the cell cycle profiles of Jurkat cells treated with ISAE. Exposing Jurkat cells to ISAE for 24 h significantly induced G2/M arrest from 12 to 21% (*p* < 0.001) at concentration around IC50 and accumulation of sub-G1 population at concentration around IC90 (Fig. 2A-C). The increased sub-G1 cell population implied apoptosis in ISAE-treated cells. This observation was further supported by the increase in the population of annexin V-positive cells and caspase 3/7 activity upon ISAE treatment (Fig. 2D and Supplementary Figs. S1 and S2). Moreover, our results showed that ISAE induced G2/M arrest in a time-dependent manner (Fig. 2E, F).

### ISAE activated ATR-CHK1-Wee1-CDC25C-CDK1 cell cycle arrest axis in Jurkat cells

**Fig. 3:**
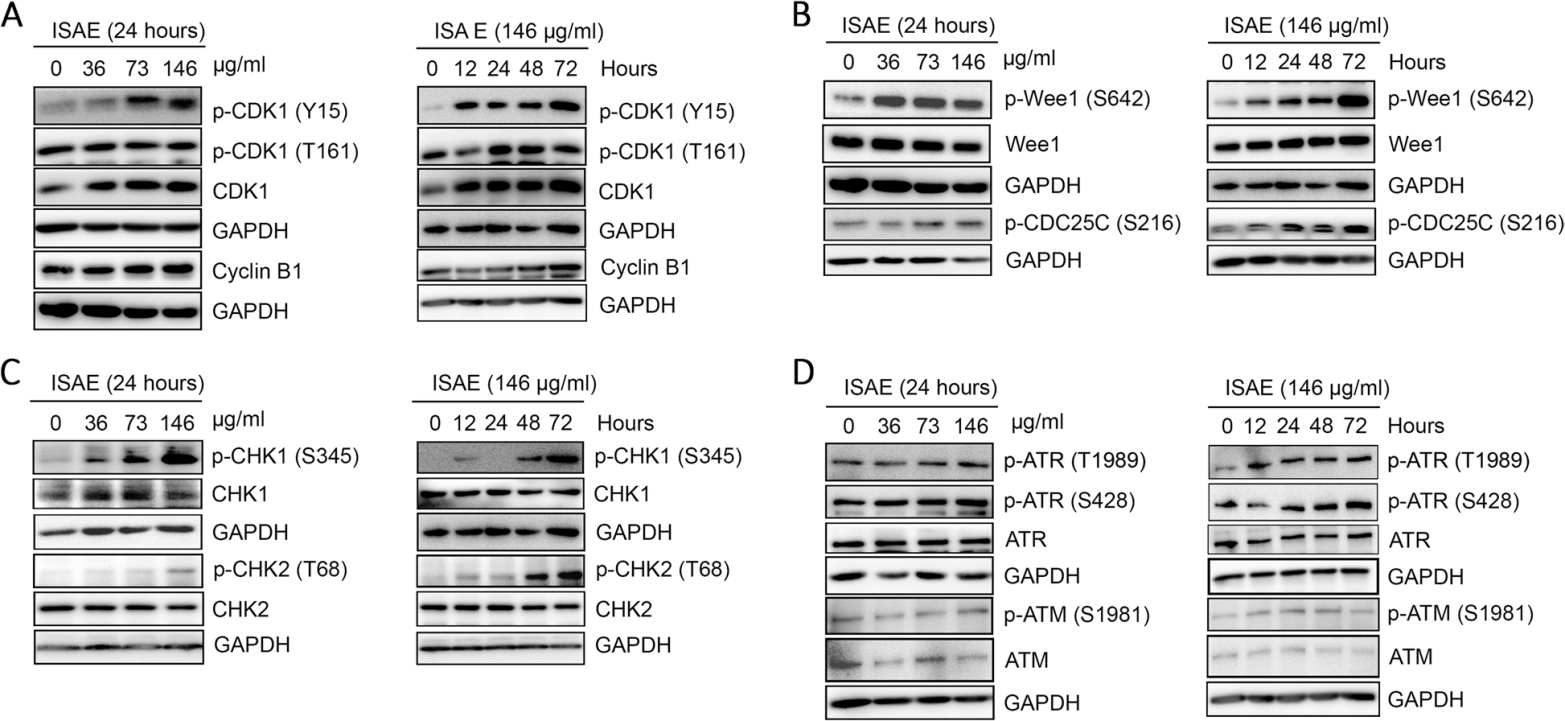
ISAE activated ATR-CHK1-Wee1-CDC25C-CDK1 axis in Jurkat cells. Jurkat cells were treated with ISAE at indicated concentrations (36, 73, 146 µg/mL) for indicated times (12, 24, 48, 72 h). Cells were harvested for western blot analysis. **A** The expressions of Cyclin-dependent kinases 1 (CDK1), phospho-CDK1 (p-CDK1 Y15), p-CDK1 (T161), and Cyclin B1. **B** The expressions of p-Wee1 (S642), Wee1, and p-CDC25 (S216). **C** The expressions of p-CHK1 (S345), p-CHK2 (T68), CHK1, and CHK2. **D** The expressions of ataxia-telangiectasia-mutated protein kinases (ATM), p-ATM (S1981), ATM- and Rad3-related protein kinases (ATR), p-ATR (T1989), and p-ATR (S428). ISAE increased the phosphorylation of CDK1 at tyrosine 15 (Y15), the phosphorylation of Wee1 at serine 642 (S642), the inhibitory phosphorylation of CDC25C at serine 216 (S216), the phosphorylation of CHK1 at serine 345 (S345), and phosphorylation of ATR at both threonine 1989 (T1989) and serine 428 (S428). The original images of each blot can be found in the Supplementary materials

Next, we investigated the molecular mechanism by which ISAE induces G2/M arrest in Jurkat cells. Our data revealed that ISAE increased the phosphorylation of CDK1 at tyrosine 15 (Y15) in concentration- and time-dependent scenarios (Fig. 3A). In contrast, ISAE had no effect on the phosphorylation of CDK1 at threonine 161 (T161) and the expression of total CDK1, and caused a slight accumulation of cyclin B1 in Jurkat cells (Fig. 3A). These results suggest that ISAE induces G2/M arrest by increasing inhibitory phosphorylation of CDK1 at Y15. Y15 is phosphorylated by Wee1 kinase which can be removed by CDC25C phosphatase [13]. We then tested which protein was responsible for the increased phosphorylation of CDK1 at Y15 after ISAE treatment. Interestingly, our results showed that both active phosphorylation of Wee1 at serine 642 (S642) and the inhibitory phosphorylation of CDC25C at serine 216 (S216) were induced by ISAE treatment in a concentration- and time-dependent scenario (Fig. 3B). Wee1 kinase and CDC25C phosphatase are targets of CHK1 and CHK2, which regulate cell cycle progression in response to DNA damage [13]. Thus, we investigated the effects of ISAE on CHK1 and CHK2. As shown in Fig. 3C, ISAE increased the phosphorylation of CHK1 at serine 345 (S345) and CHK2 at threonine 68 (T68) in a concentration- and time-dependent manner, although CHK2 appeared to respond to ISAE treatment after 24 h. Next, we tested whether ISAE could activate ataxia-telangiectasia-mutated (ATM) and ATM- and Rad3-related (ATR) protein kinases that act upstream of CHK1/CHK2. We found that ISAE triggered a notable elevation in ATR phosphorylation at both threonine 1989 (T1989) and serine 428 (S428) at a concentration of 146 µg/ml (Fig. 3D). In contrast, no changes in the expression of phospho-ATM (S1981) were observed in any of the ISAE-treated groups (Fig. 3D). Taken together, these findings suggest that ISAE triggers G2/M arrest in Jurkat cells via the ATR-CHK1-Wee1-CDC25C-CDK1 axis.

### ATM/ATR kinase inhibitor rescued ISAE-mediated G2/M arrest

**Fig. 4:**
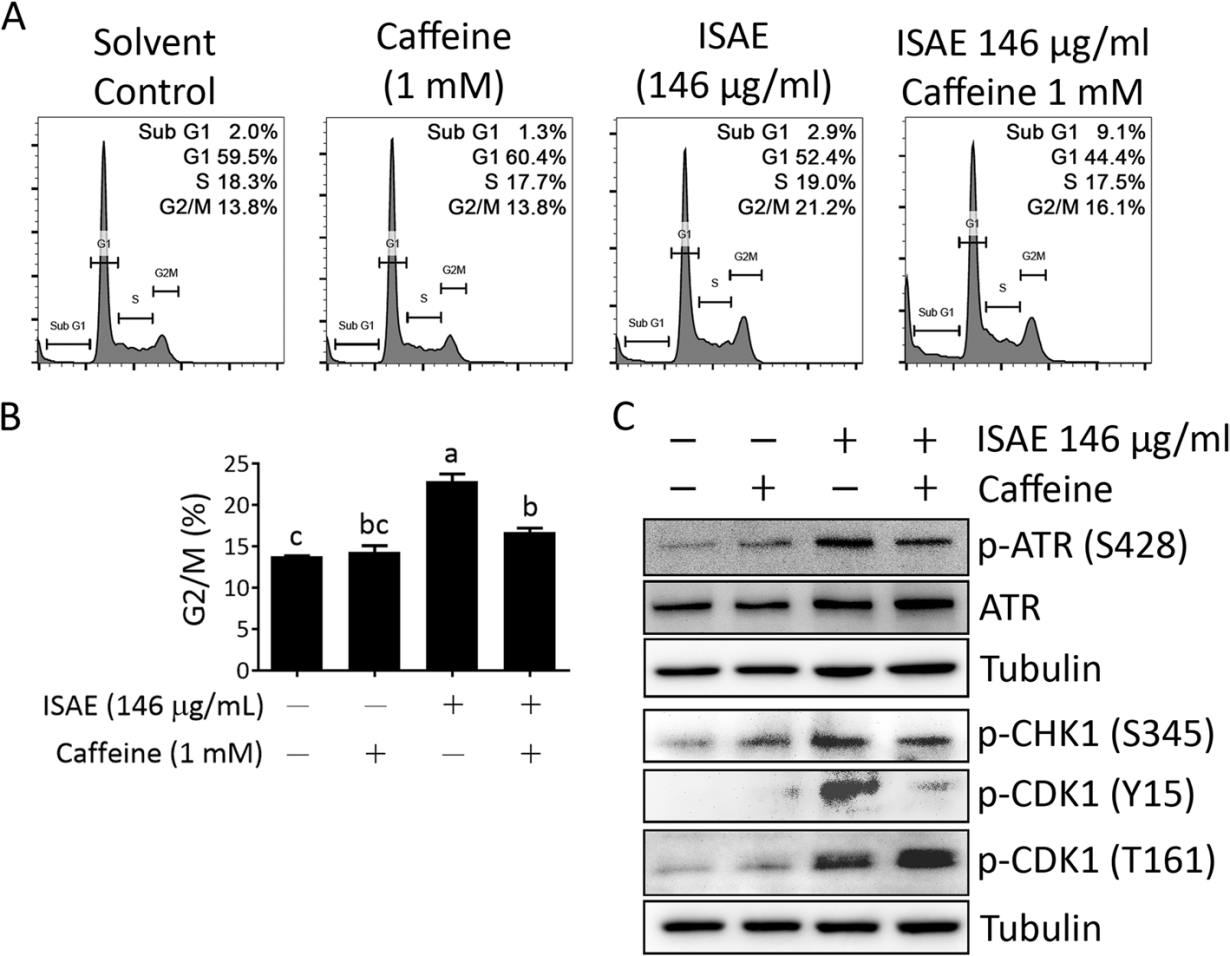
Inhibition of ATM/ATR signaling rescued ISAE-mediated G2/M arrest. Jurkat cells were pre-exposed for 1 h to Caffeine (1 mM), a widely used ATM/ATR inhibitor, and then treated with ISAE (146 µg/mL) for 48 h. Cells were then harvested for cell cycle analysis (**A**-**B**) and western blot (**C**). **A** Representative result of cell cycle analysis. **B** Quantitative results of G2/M phase of panel (**A**). Data are presented as mean ± SD from three independent experiments. Statistical analysis was performed using ANOVA, and different superscript letters indicate statistically significant differences (*p* < 0.05). **C** The expressions of p-ATR (S428), ATR, p-CHK1 (S345), p-CDK1 (Y15), and p-CDK1 (T161). Caffeine abolished ISAE-induced ATR singling and rescued ISAE-mediated G2/M arrest. The original images of each blot can be found in the Supplementary materials

To confirm the involvement of ATR signaling in ISAE-induced G2/M arrest, we tested the effect of ISAE in the presence of caffeine, a widely used ATM/ATR inhibitor [14]. Jurkat cells were pre-treated with caffeine for 1 h and then treated with ISAE in the presence of caffeine. The cell cycle profile and phosphorylation status of the components of ATR signaling were determined. As shown in Fig. 4A and B, caffeine significantly rescued ISAE-mediated G2/M arrest (*p* < 0.05). Furthermore, caffeine clearly diminished ATR signaling, as evidenced by the reduction of phospho-ATR (S428), phospho-CHK1 (S345), and phospho-CDK1 (Y15) in the caffeine/ISAE co-treatment group compared to the ISAE single-treated group (Fig. 4C). Accordingly, these findings support the notion that ISAE-mediated G2/M arrest in Jurkat cells is ATR signaling-dependent.

### ISAE induced DNA damage in Jurkat cells

**Fig. 5:**
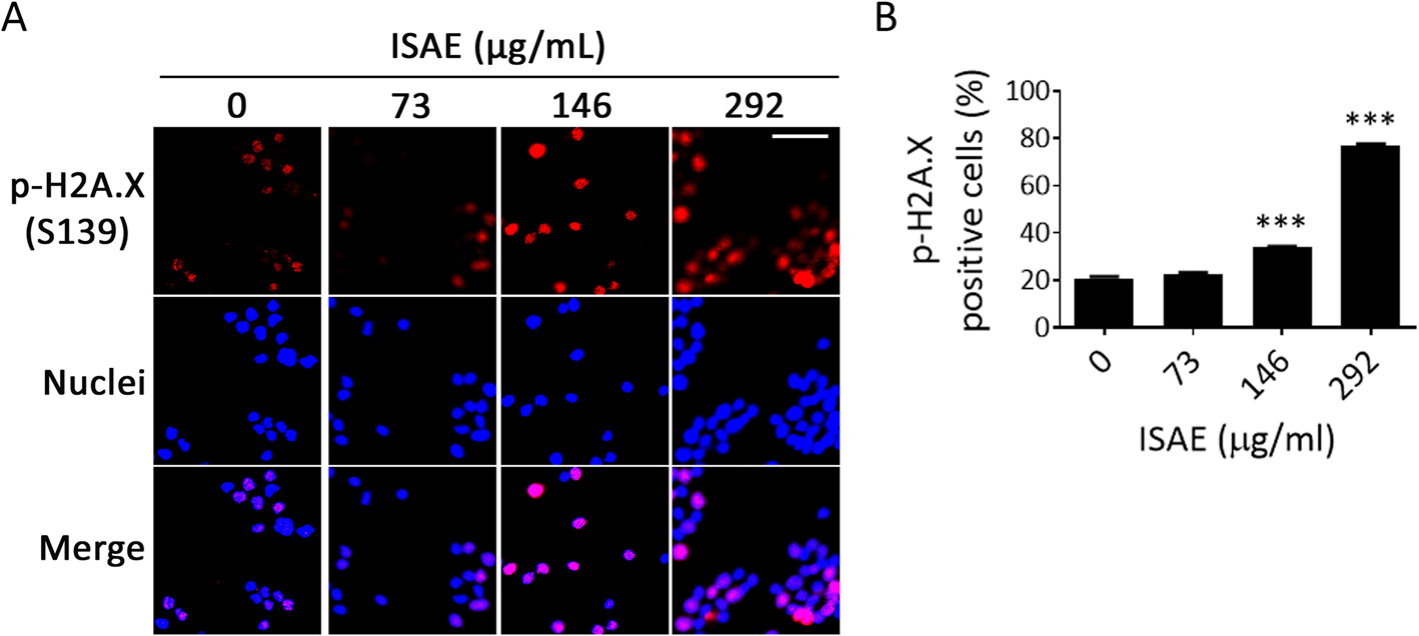
ISAE increased γ-H2A.X expression in Jurkat cells. **A** Jurkat cells were treated with indicated concentrations (73, 146, 292 µg/mL) of ISAE for 24 h. The expression of γ-H2A.X (phosphorylation of histone H2A.X at serine 139 (phospho-H2A.X (S139)) was revealed by immunofluorescence staining. Nuclei were stained with Hoechst 33258. Bar: 35 μm. **B** Quantitative results of panel (**A**). The graph presents the percentage of positive phospho-H2A.X (S139) staining cells of six fields for each slide. * indicates significant difference from control group (***p < 0.001, one-way ANOVA). ISAE significantly increased the number of γ-H2A.X foci positive cells

ATR plays a regulatory role in initiating the cell cycle arrest cascade following DNA damage [15]. Thus, it is reasonable to speculate that ISAE might activate ATR and the subsequent G2/M arrest by inducing DNA damage in Jurkat cells. Phosphorylation of histone H2A.X at serine 139 (γ-H2A.X) by ATM/ATR is one of the most sensitive markers of DNA damage. Thousands of γ-H2A.X molecules form foci at DNA break sites and can be detected using fluorescence microscopy [16]. As shown in Fig. 5, ISAE significantly increased the number of γ-H2A.X focus-positive cells (*p* < 0.001). These results indicated that ISAE could induce DNA damage in Jurkat cells.

### Characterizing the chemical constituents of ISAE using tandem mass spectroscopy and molecular networking approach

**Fig. 6:**
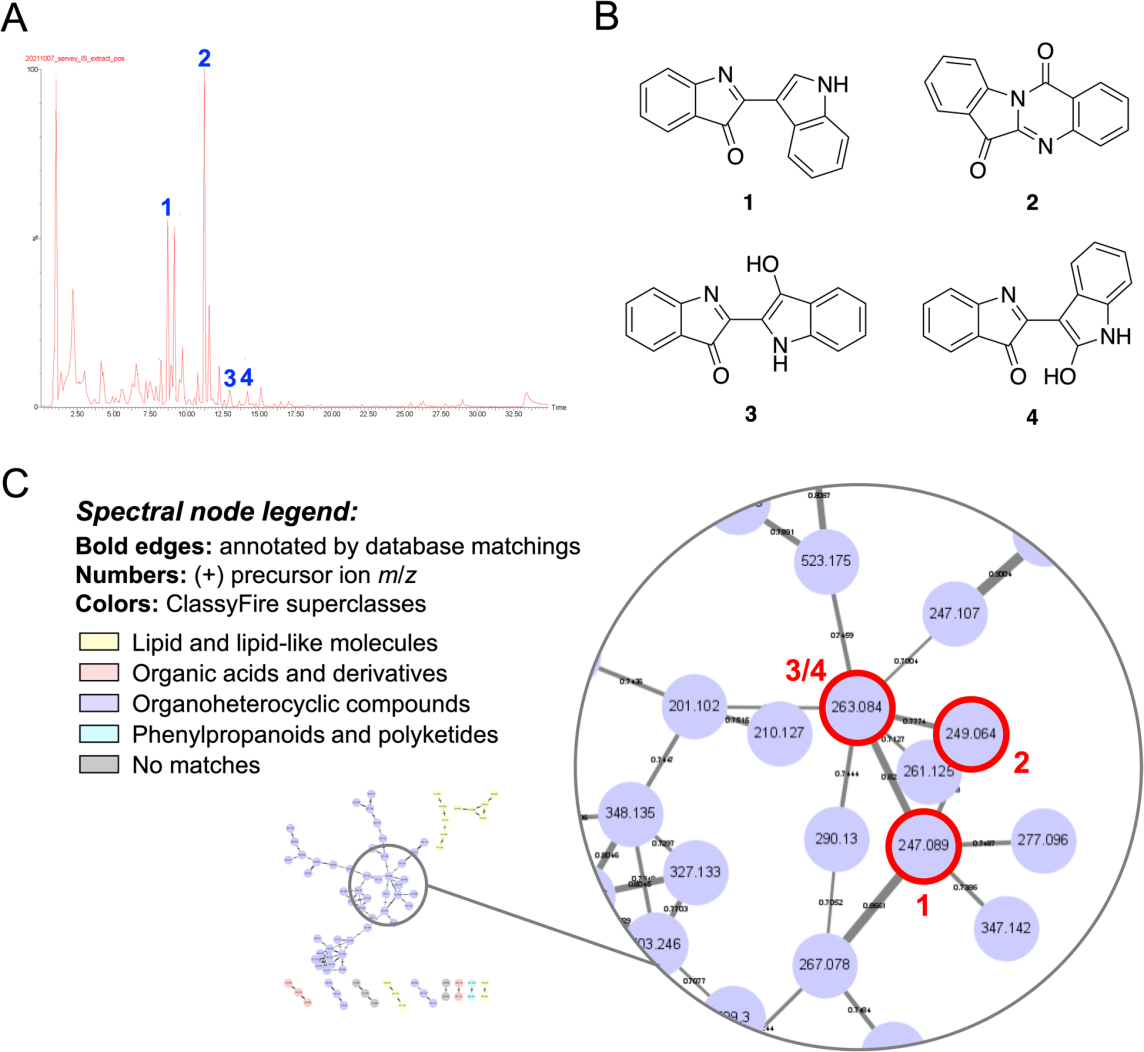
MS/MS spectroscopy coupled molecular networking illustrated the metabolites contained in ISAE. **A** The Ultra-Performance Liquid Chromatography (UPLC)-Mass (MS) total ion chromatogram in positive mode. **B** The chemical structures of identified constituents, including 2-(indol-3-yl)-3H-indol-3-one (1), tryptanthrin (2), indigo (3), and indirubin (4). **C** The classical molecular network spectral nodes colored according to ClassyFire superclasses. ClassyFire software from Global Natural Products Social (GNPS) platform revealed the majority of organoheterocyclic metabolites of ISAE

To identify the bioactive indicators, ultra-performance liquid chromatography quadrupole time-of-flight mass spectrometry (UPLC-QTOF-MS) was used to carry out a qualitative analysis of ISAE. The acquired non-targeted MS^2^ data were then interpreted and annotated based on the GNPS and Reaxys databases, resulting in the identification of four indole alkaloids: 2-(indol-3-yl)-3H-indol-3-one (1) [17], tryptanthrin (2) [18], indigo (3) [17], and indirubin (4) [17] (Fig. 6A, B). Furthermore, the visualized molecular networking using ClassyFire software from the GNPS platform revealed most organoheterocyclic metabolites of ISAE (Fig. 6C).

### Tryptanthrin is one of active components in ISAE

**Fig. 7:**
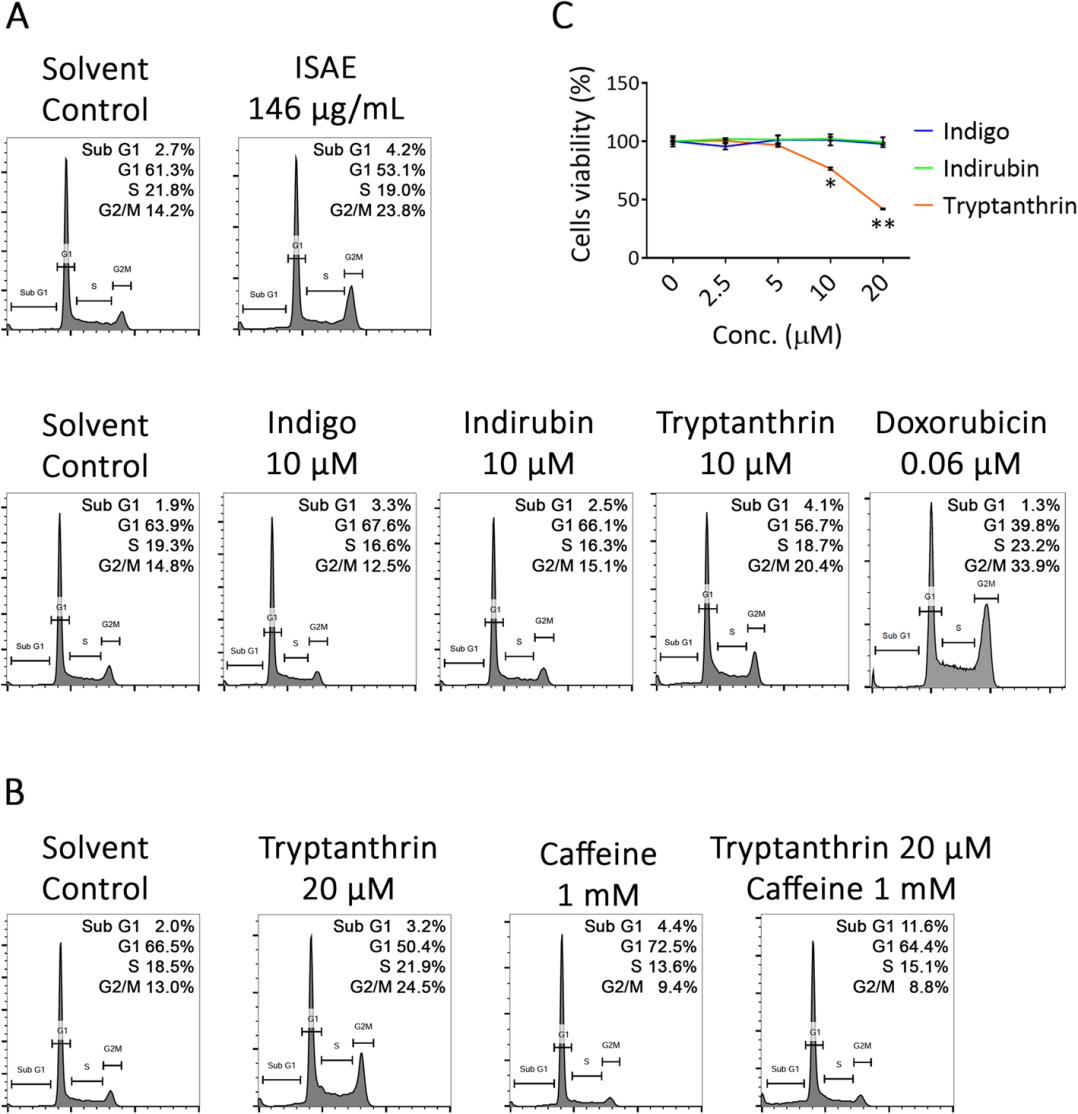
Tryptanthrin affected cell cycle profile resembled that of ISAE. **A** Jurkat cells were exposed to indicated concentrations of ISAE (146 µg/mL), indigo (10 µM), indirubin (10 µM), tryptanthrin (10 µM), or doxorubicin (0.06 µM) for 24 h, then fixed for propidium iodide (PI) staining. Cell cycle profiles were recorded by flow cytometry. Control solvent was distilled water for ISAE and Dimethyl sulfoxide (DMSO) at final concentration of 0.1% for pure compounds. **B** Jurkat cells were pre-exposed for 1 h to Caffeine, and then treated with tryptanthrin (20 µM) for 24 h. Cells were then harvested for cell cycle analysis. **C** Jurkat cells were treated with indicated concentrations (2.5, 5, 10, 20 µM) of indigo, indirubin, or tryptanthrin for 72 h. The resazurin reagent was used to determine cell viability. DMSO at 0.1% was used as solvent control (“0” concentration) for 100% viability. Data are presented as mean ± SD from three independent experiments. * indicates significant difference from control group (*p < 0.05, **p < 0.01, one-way ANOVA). Tryptanthrin, but not indigo nor indirubin, can induce G2/M arrest to a similar extent as ISAE did in Jurkat cells. Co-treatment with caffeine blocked tryptanthrin-induced G2/M arrest in Jurkat cells

Next, we performed selected MS ion scan experiments for the relative quantification analysis of three commercially available compounds, including tryptanthrin (2), indigo (3), and indirubin (4). The relative amounts of tryptanthrin and indirubin were analyzed to be 3.74% and 1.55%, respectively (Supplementary Fig. S3). These amounts are equivalent to 21.9 µM of tryptanthrin and 8.6 µM of indirubin when cells were treated with 146 µg/mL of ISAE. Given the established ability of tryptanthrin derivatives to inhibit Topoisomerase II (TopoII) and induce G2/M cell cycle arrest [19], we focused our investigation on the specific impact of tryptanthrin on cell cycle dynamics in Jurkat cells. Cell cycle analysis revealed that tryptanthrin, akin to doxorubicin—a well-known TopoII inhibitor widely used in leukemia treatment—effectively induced G2/M arrest in Jurkat cells (Fig. 7A). Notably, at a concentration of 10 µM, tryptanthrin demonstrated a comparable level of cell cycle arrest (20%) to that induced by ISAE (23%) in Jurkat cells. Conversely, neither indigo nor indirubin, at similar concentrations, significantly induced G2/M arrest, as evidenced by lower rates of 12% and 15%, respectively (Fig. 7A). Importantly, the effects of tryptanthrin at 20 µM on the cell cycle profile and cell viability of Jurkat cells closely resembled those observed in 146 µg/mL ISAE-treated cells (Figs. 1A, and 7B, C). Moreover, we demonstrated that co-treatment with caffeine blocked tryptanthrin-induced G2/M arrest in Jurkat cells (Fig. 7B), which was in accordance with the above results. Taken together, these findings support the notion that tryptanthrin, but not indigo or indirubin, is the main active component of ISAE responsible for its anti-leukemic activity.

## Discussion

*I. suffruitcosa* Mill., is a Taiwanese folk medicine used to treat leukemia. In this study, we investigated the anti-leukemic effect of ISAE. Our data indicated that ISAE caused cytotoxicity in Jurkat cells without affecting normal fibroblasts (Ccd-996sk). It induced G2/M arrest via the ATR/CHK1/Weel/CDC25C signaling pathway, which might be the result of ISAE-induced DNA damage. Furthermore, we analyzed the chemical constituents of ISAE and identified that tryptanthrin is abundant in ISAE and responsible for its anti-leukemic activity. To the best of our knowledge, this is the first study to delineate the anti-leukemic mechanism of *I. suffruitcosa* and profile the chemical constituents in the active extract.

By utilizing tandem mass spectroscopy and a molecular networking approach, we found that organoheterocyclic compounds mainly comprised ISAE, of which bisindole alkaloids could be the main components. According to the annotation of the MS^2^ data, our findings suggest the presence of 2-(indol-3-yl)-3H-indol-3-one or indoxyl red (1) in *I. suffruitcosa*. Although several methods have been reported to synthesize indoxyl red [20], to the best of our knowledge, our findings are the first to imply the natural existence of indoxyl red in plants. Currently, as indoxyl red is not commercially available, further studies are required to isolate this compound from ISAE and confirm its structure by nuclear magnetic resonance spectrometry. Moreover, Song et al. demonstrated that indoxyl red showed cytotoxic activity against the mouse B-cell lymphoma cell line NSF202 [20]. Thus, it would also be interesting to investigate whether indoxyl red contributes to the anti-leukemic effect of ISAE.

We also showed the presence of tryptanthrin (2), indigo (3), and indirubin (4) in ISAE. Indirubin is a key active component of indigo naturalis (IN) [21]. IN, also known as “Qingdai” in Chinese, is a deep blue powder derived from the leaves or stems of plants like *Strobilanthes cusia* (Nees) Kuntze, *Persicaria tinctoria* (Aiton) Spach, and *Isatis tinctoria* L [21]. Notably, *I. suffruticosa* has been documented as a potential source for Qingdai preparation [22, 23]. For over 1,400 years, IN/Qingdai has been used in traditional Chinese medicine to treat conditions such as hemoptysis, epistaxis, chest pain, aphthous ulcers, and infantile convulsions [21]. By the 1970s, IN gained attention for its therapeutic potential in treating various cancers, leading to the introduction of indirubin as a novel treatment for leukemia in China [21]. Several compounds, including indigo, indirubin, tryptanthrin, among others, are believed to contribute to the medicinal properties of IN [21, 24, 25]. Regarding the mechanism of action, indirubin and its derivatives target STAT5 and CDKs, leading to G1 or G2/M arrest [26–29]. On the other hand, tryptanthrin and its related compounds inhibit STAT3, reduce the expression of cyclin D2, and induce G1 arrest [30, 31]. In addition, tryptanthrin derivatives can reduce the expression of cycline A1, cycline B1, and CDK2, resulting in S-phase arrest in hepatocellular carcinoma cells [32]. A derivative of tryptanthrin, benzo[b]tryptanthrin, has been identified as a non-intercalative catalytic inhibitor of topoI/II that exhibits cytotoxicity against cancer cells [19]. Here, we demonstrated that tryptanthrin, instead of indirubin or indigo, induced G2/M arrest in Jurkat cells, which resembles the effect produced by ISAE. Similarly, the G2/M arrest activity of tryptanthrin can be blocked by an ATR inhibitor. These findings not only indicate that trypthathrin is the major active component of ISAE, but also suggest that tryptanthrin may be able to exert anti-cancer activity through diverse mechanisms in diverse cell types. Based on these findings, it seems reasonable to speculate that 2-(indol-3-yl)-3H-indol-3-one may also have no effect on the cell cycle in Jurkat cells because of its structural similarity to indirubin.

Interestingly, ISAE did not appear to be cytotoxic to acute promyelocytic leukemia (APML) HL-60 cells and acute myelocytic leukemia (AML) KG-1 cells. Since the potential mechanism of action of ISAE in Jurkat cells could be part of DNA damage response signaling, we speculated that the difference in the sensitivity to ISAE between Jurkat (acute lymphoblastic leukemia, ALL) cells and HL-60 and KG-1 could be associated with DNA damage repair (DDR) systems. Previous studies have demonstrated that proteins involved in DDR are differentially expressed in Jurkat cells and HL-60 [33, 34]. However, a previous report showed that tryptanthrin, at the concentration around 25 µM, caused a 50% cytotoxicity in HL-60 cells [35]. We found that the amount of tryptanthrin in the ISAE was 21.9 µM. Thus, it is also possible that other components of ISAE may counteract the cytotoxic effects of tryptanthrin in HL-60 and KG-1 cells, but not in Jurkat cells.

From a chemical structure perspective, tryptanthrin, like the TopoI inhibitor camptothecins and the TopoII inhibitor doxorubicin, possesses a planar polycyclic structure. Although camptothecins and doxorubicin have different molecular mechanisms, both can induce DNA damage and G2/M cell cycle arrest [36, 37]. Interestingly, previous studies have shown that ALL cells respond to doxorubicin-induced DNA damages by activating the ATR-CHK1 pathway [36]. Additionally, derivatives of tryptanthrin have also been found to exhibit inhibitory effects on TopoII [19]. Therefore, we believe that tryptanthrin might exert anti-leukemic activity through a mechanism similar to that of doxorubicin. Another interesting observation was that the combination of ISAE and caffeine induced a slight increase in the sub-G1 population. This observation aligns with findings from previous studies that investigated the combined effects of TopoI/II inhibitors and ATR inhibitors. Hur et al. showed that belotecan, a topoisomerase I inhibitor, induced DNA damage, activated the ATR pathway, and consequently caused G2/M arrest [37]. However, when AZD6738, an ATR inhibitor, was added to belotecan, the phosphorylation of ATR induced by belotecan alone was suppressed. The increase in sub-G1 and decrease in G2/M indicated the release of G2/M arrest and the induction of apoptosis. They inferred that belotecan induces DNA damage and leads to mitotic exit, that is, G2/M arrest, via the ATR/CDC25C/CDK1 axis. In contrast, ATR inhibition by the addition of AZD6738 to belotecan reverses this process and allows DNA-damaged cells to enter mitosis, leading to mitotic catastrophe [37]. Similar results were reported by Ghelli Luserna Di Rorà et al., demonstrating that doxorubicin activates the ATR/CHK1 pathway and induces G2/M cell cycle arrest in ALL cell lines [36]. Following treatment with the ATR inhibitor VE-821 or the CHK1 inhibitor prexasertib on doxorubicin-exposed cells, their study revealed that inhibiting the ATR-CHK1 pathway enhances the cytotoxic effects of doxorubicin in ALL cells [36]. This notion is further supported by studies showing the synergistic anti-cancer effects of the combination of ATR inhibition and DNA damage–inducing chemotherapy [38, 39]. ATR plays a pivotal role in DNA damage response by activating cell cycle arrest and DNA repair [40]. Activation of oncogenes and loss of G1 checkpoint control drives replication and increases replication stress. DNA damage induced by chemotherapy or radiotherapy simultaneously with ATR inhibition in the context of heightened levels of replication stress could overwhelm the ability of cancer cells to repair damaged DNA and lead to synergistic anti-cancer effects [40]. The phospho-H2A.X staining results suggest that ISAE caused DNA damage. Treated ISAE at a high concentration of ISAE (IC_90_) resulted in a profound increase in phospho-H2A.X positvie cells, suggesting severe DNA damage, which was accompanied by a sharply elevated sub-G1 population and annexin V-positive population, whereas treatment with ISAE at sub-IC_50_ concentration may only cause moderate DNA damage and induce G2/M arrest. Thus, it is worth investigating the combinatory effect of potent and selective ATR inhibitors with ISAE as well as tryptanthrin.

Several major advances have been made in the treatment of adult ALL. Current treatment options for adult and elderly patients with ALL are not sufficient, 60–70% of these patients do not achieve 5-year overall survival [41]. In particular, the relapsed T cell acute lymphoblastic leukemia (T-ALL) in adult patients is incurable, with less than 10% cases surviving at 5 years [42]. Thus, there is an urgent need for new drugs to supplement the current treatment and improve patient outcomes. Herein, we report that the ISAE possesses anti-leukemic activity in Jurkat cells while showing mild cytotoxic effects in normal fibroblast cells. Thus, it is worth investigating the potential of utilizing ISAE or tryptanthrin as a adjuvant agents for T-ALL treatment.

## Conclusion

Our studies revealed that ISAE induces G2/M arrest in the T cell acute lymphoblastic leukemia cell line—Jurkat cells, and stimulates the ATR/CHK1/Wee1/CDC25C signaling pathway. Through tandem mass spectroscopy analyses, we detected the presence of tryptanthrin and indirubin in ISAE. Further experiments suggested that tryptanthrin could be the main component responsible for the cell cycle arrest activity of ISAE on Jurkat cells. These observations provide subtle support to the traditional use of *I. suffruitcosa* in Taiwanese folk medicine for leukemia treatments.

### Supplementary Information

**Additional file 1: Supplementary Table S1.** Antibodies used in this study. **Supplementary Fig. S1.** Representative plot of Annexin V staining results. This figure is related to Fig. 2D. **Supplementary Fig. S2.** The caspase-3/7 activities in Jurkat cells after 12 h of ISAE treatment were assessed by Caspase-Glo® 3/7 assay (Promega) according to the manufacturer's instructions. The graph was expressed as fold changes to control group. **Supplementary Fig. S3.** Relative quantitative analysis of tryptanthrin, indigo, and indirubin in ISAE. The selected ion current chromatograms of the ISAE extract, tryptanthrin, indigo, and indirubin were carried out through MS full scan experiment in positive mode. The relative abundance of the three selected compounds in ISAE extract were calculated based on comparing the peak area ratios with standard compounds. **Supplementary Fig. S4.** Cell gating for cell cycle analysis. For cell cycle analysis, cells were gated using forward scatter (FSC) and side scatter (SSC) properties. This helped in excluding cell debris and selecting the desired cells (within the black circle) for analysis. The gating area was established based on the solvent control group (0 μg/mL of ISAE) and was consistently applied to all other groups. This illustration corresponds to Fig. 2A. **Original images of western blot. Multiple exposure images of WB.**

## Data Availability

The data presented in this study are available on request from the corresponding author.

## Abbreviations

ISAE: *I. suffruticosa* aerial parts extract
ALL: Acute lymphoblastic leukemia
APML: Acute promyelocytic leukemia
AML: Acute myelocytic leukemia
RPMI: Roswell Park Memorial Institute
DMEM: Dulbecco’s modified Eagle’s medium
FBS: Fetal bovine serum
PI: Propidium iodide
UPLC-QTOF-MS: Ultra-Performance Liquid Chromatography Quadrupole Time-of-Flight Mass Spectrometry
GNPS: Global Natural Products Social
ATM: Ataxia-telangiectasia-mutated protein kinases
ATR: ATM- and Rad3-related protein kinases
CDK1: Cyclin-dependent kinases 1
DDR: DNA damage repair
IC50: 50% maximal inhibitory concentration
