# Supplementary material for: Indigofera suffruticosa aerial parts extract induce G2/M arrest and ATR/CHK1 pathway in Jurkat cells
Source: BMC Complement Med Ther. 2024 Jan 9;24:28. doi: 10.1186/s12906-023-04325-w (PMC10775588; doi:10.1186/s12906-023-04325-w)

Supplementary materials for

Indigofera suffruticosa aerial parts extract induces ATR/CHK1 pathway and G2/M arrest in Jurkat cells

Hong-Loan Tran, Kuei-Hung Lai, Hsun-Shuo Chang, Yi-Siao Chen, Hui-Chun Wang, Shuen-Shin Yang, Hsueh-Wei Chang, Chin-Mu Hsu, Chia-Hung Yen*, and Hui-Hua Hsiao*

Content:

Supplementary Table S1.
Supplementary Fig. S1.
Supplementary Fig. S2.
Supplementary Fig. S3.
Supplementary Fig. S4.
Original images of western blot
Multiple exposure images of western blot

Supplementary Table S1: Antibodies used in this study

Targets	Vendors	Catalogy No.
p-CDC25 (S216)	Cell signaling	9528
p-CHK1 (S345)	Cell signaling	2348
CHK1	BETHYL	800-338-9579
p-CHK2 (T68)	Cell signaling	2197
CHK2	Cell signaling	3440
p-ATR (S428)	Cell signaling	2853
p-ATR (T1989)	GeneTex	41354
ATR	Abnova	PAB9924
p-ATM (T1981)	EPITOMIC	YH101212D
ATM	Gene Tex	GTX70103
p-CDK1 (Y15)	Cell signaling	4539
p-CDK1 (T161)	Cell signaling	9114
CDK1	Cell signaling	77055
p-Wee1 (S642)	Cell signaling	4910
Wee1	Cell signaling	13084
Cyclin A	Cell signaling	4656
Cyclin B1	Cell signaling	12231
Cyclin E2	Cell signaling	4132
p-H2A.X (S139)	MILLPORE	05-636
Anti-rabbit IgG, HRP-linked Antibody	Cell signaling	7074
Anti-mouse IgG, HRP-linked Antibody	Cell signaling	7076
Goat anti-Mouse IgG (H+L) Secondary Antibody, Alexa Fluor 594	Invitrogen	R37121

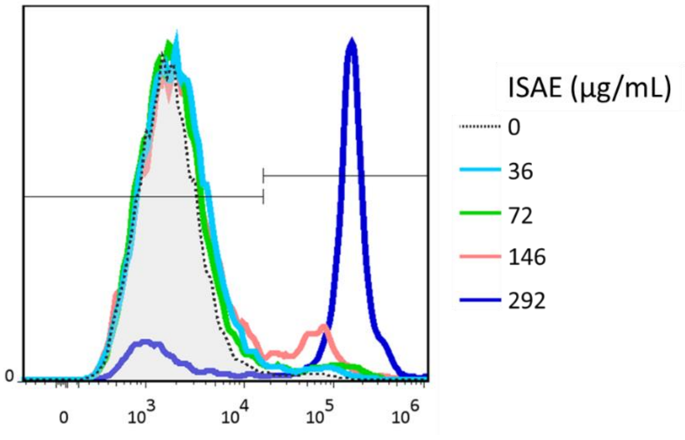

Supplementary Fig. S1. Representative plot of Annexin V staining results. This figure is related to Fig. 2D.

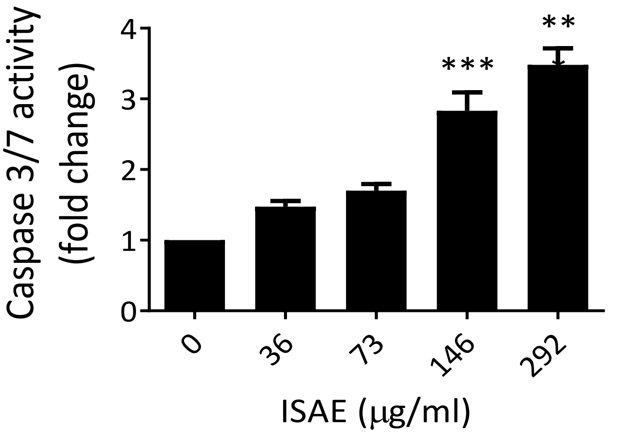

Supplementary Fig. S2. The caspase-3/7 activities in Jurkat cells after 12 hours of ISAE treatment were assessed by Caspase-Glo® 3/7 assay (Promega) according to the manufacturer's instructions. The graph was expressed as fold changes to control group.

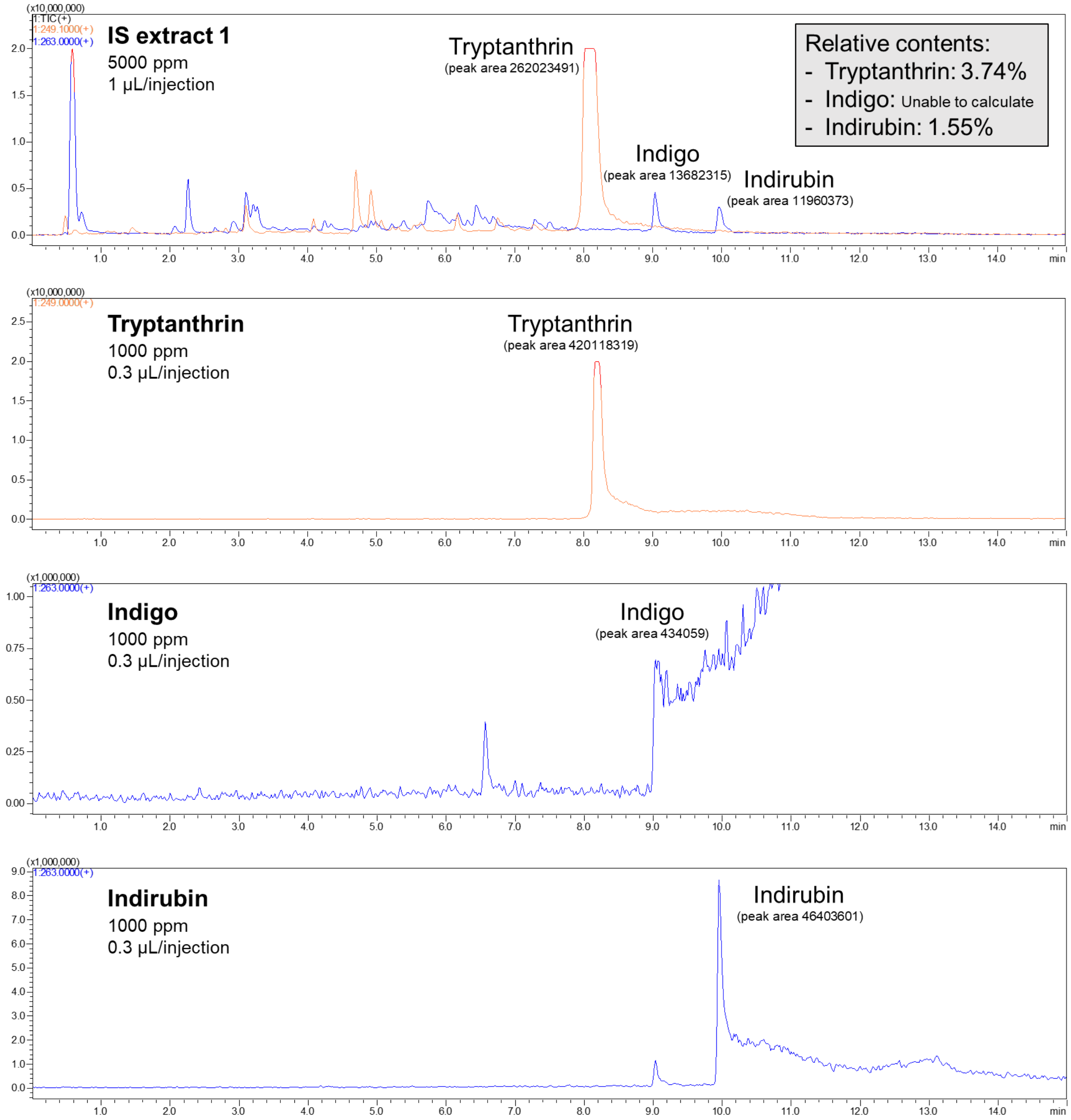

Supplementary Fig. S3. Relative quantitative analysis of tryptanthrin, indigo, and indirubin in ISAE. The selected ion current chromatograms of the ISAE extract, tryptanthrin, indigo, and indirubin were carried out through MS full scan experiment in positive mode. The relative abundance of the three selected compounds in ISAE extract were calculated based on comparing the peak area ratios with standard compounds.

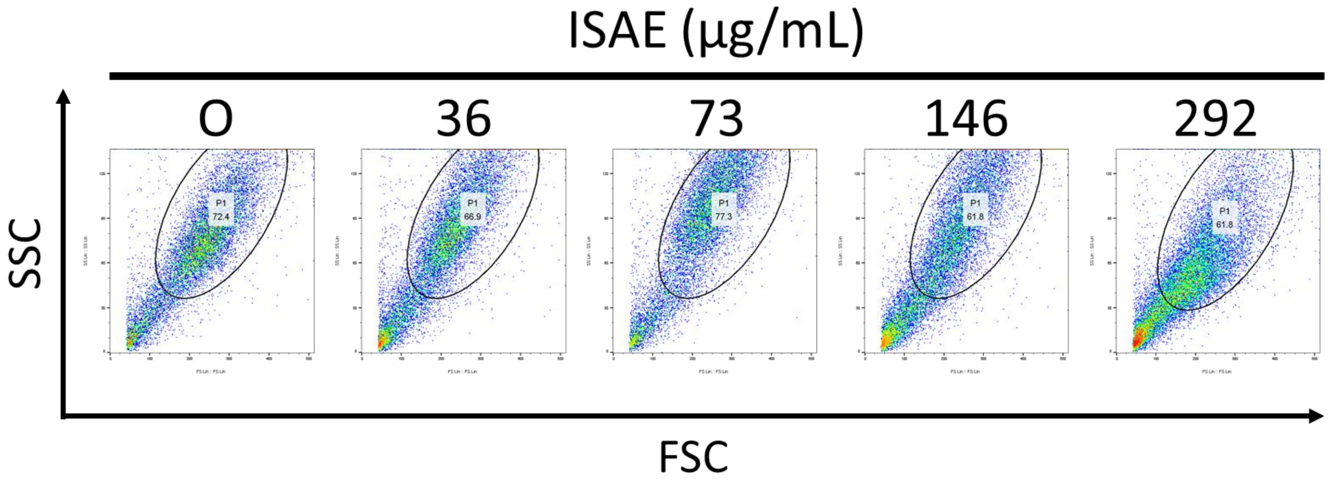

Supplementary Fig. S4. Cell gating for cell cycle analysis. For cell cycle analysis, cells were gated using forward scatter (FSC) and side scatter (SSC) properties. This helped in excluding cell debris and selecting the desired cells (within the black circle) for analysis. The gating area was established based on the solvent control group (0 μg/mL of ISAE) and was consistently applied to all other groups. This illustration corresponds to Fig. 2A.

Original images of western blot

Figure 3A-left panel

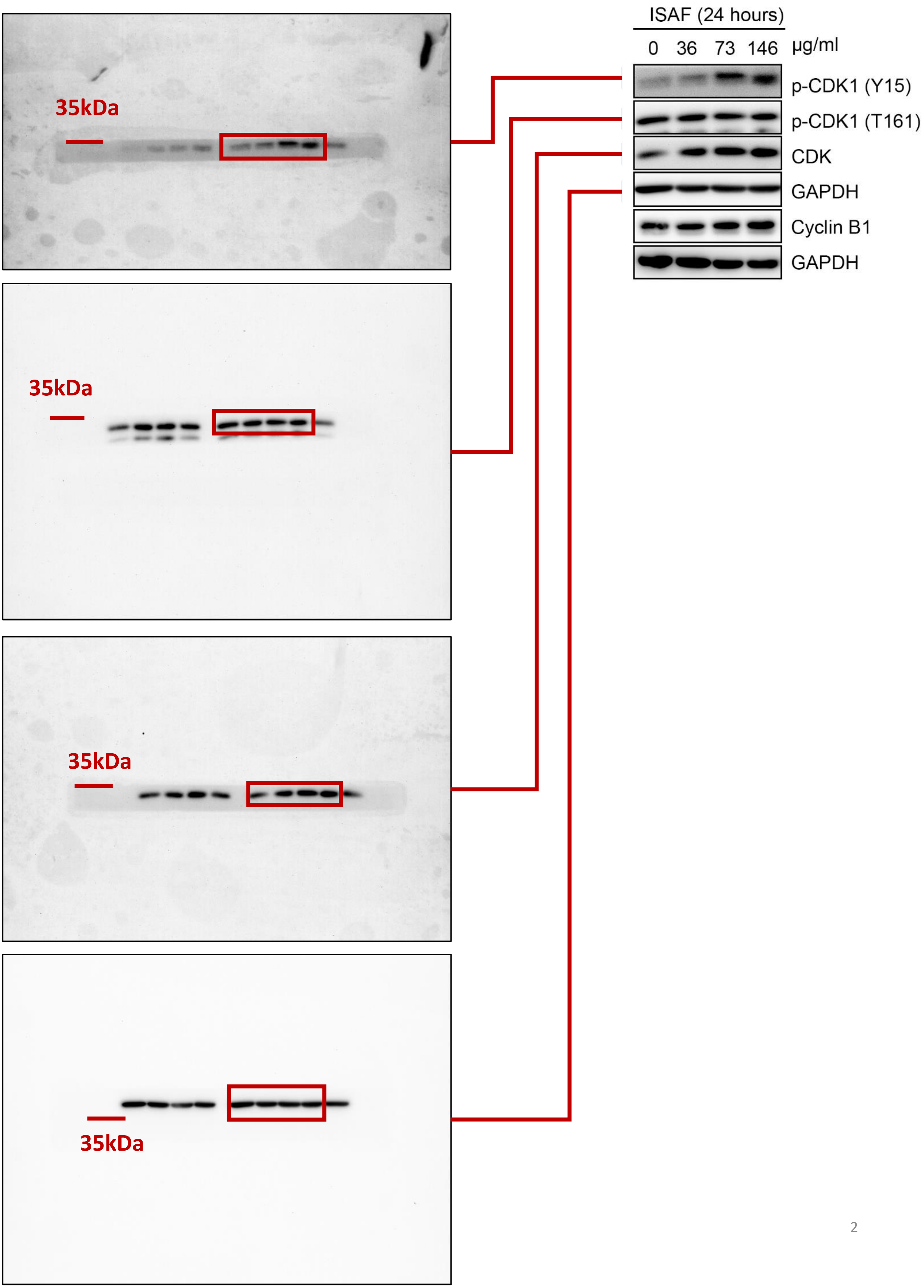

Figure 3A-left panel

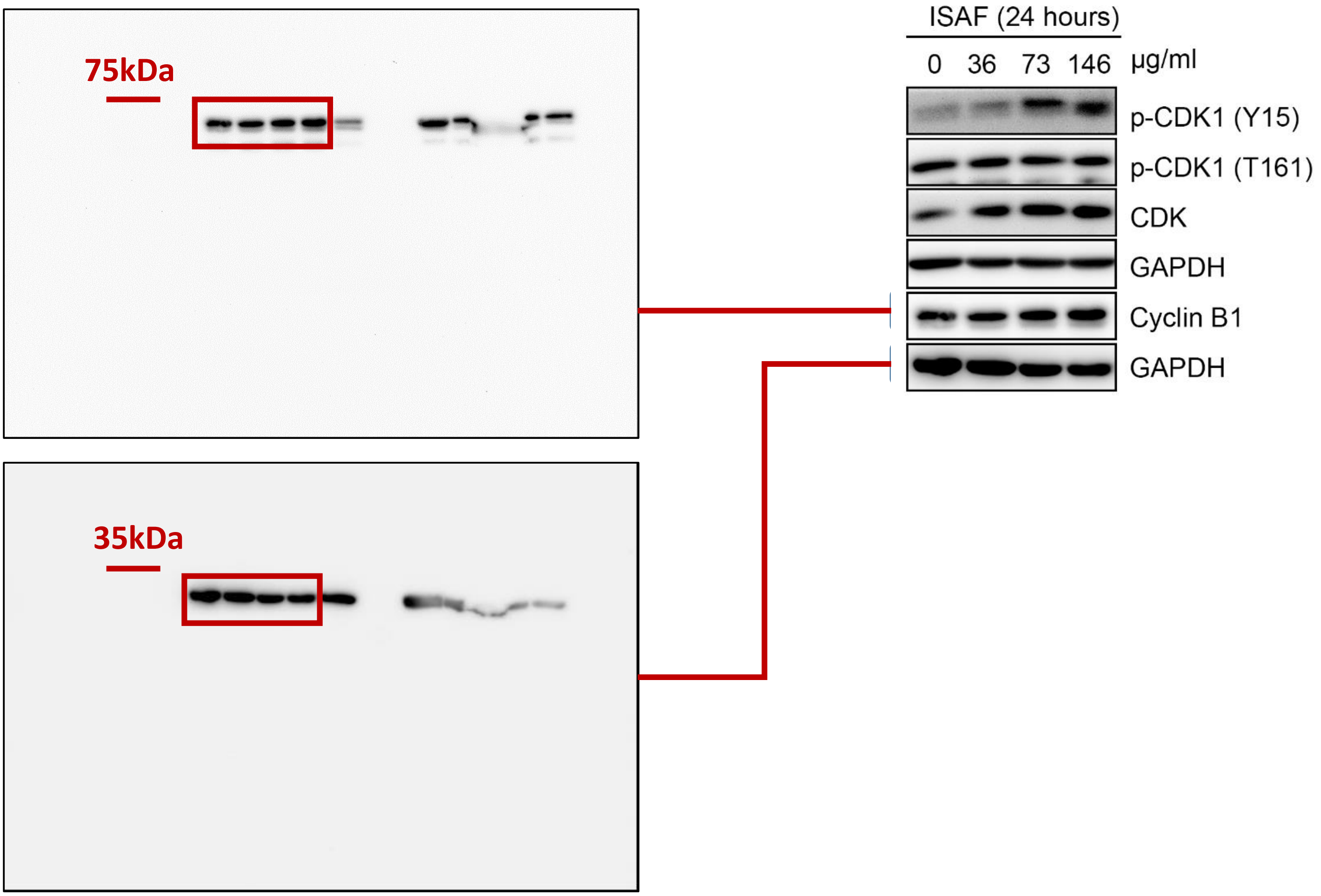

Figure 3A-right panel

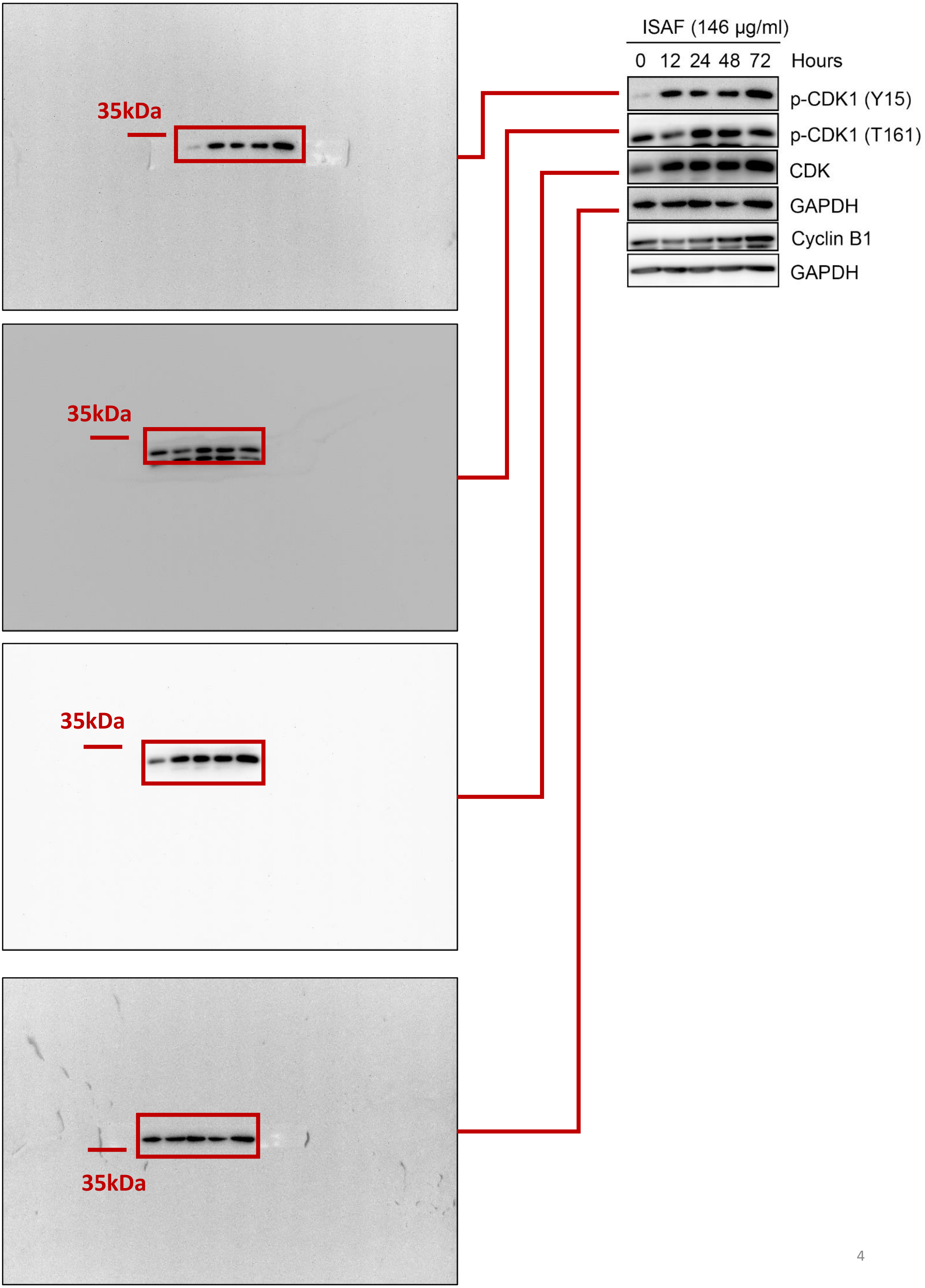

Figure 3A-right panel

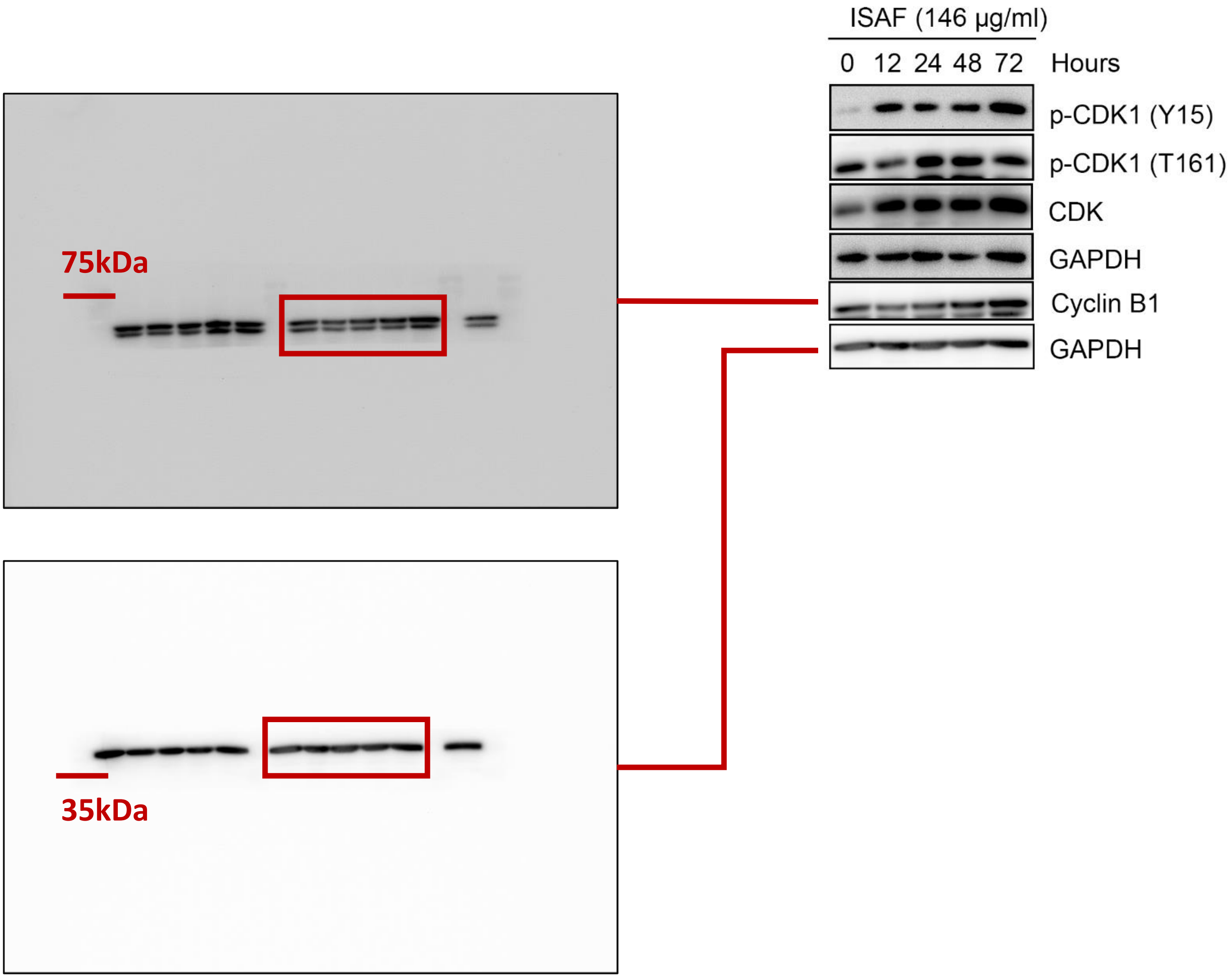

Figure 3B-left panel

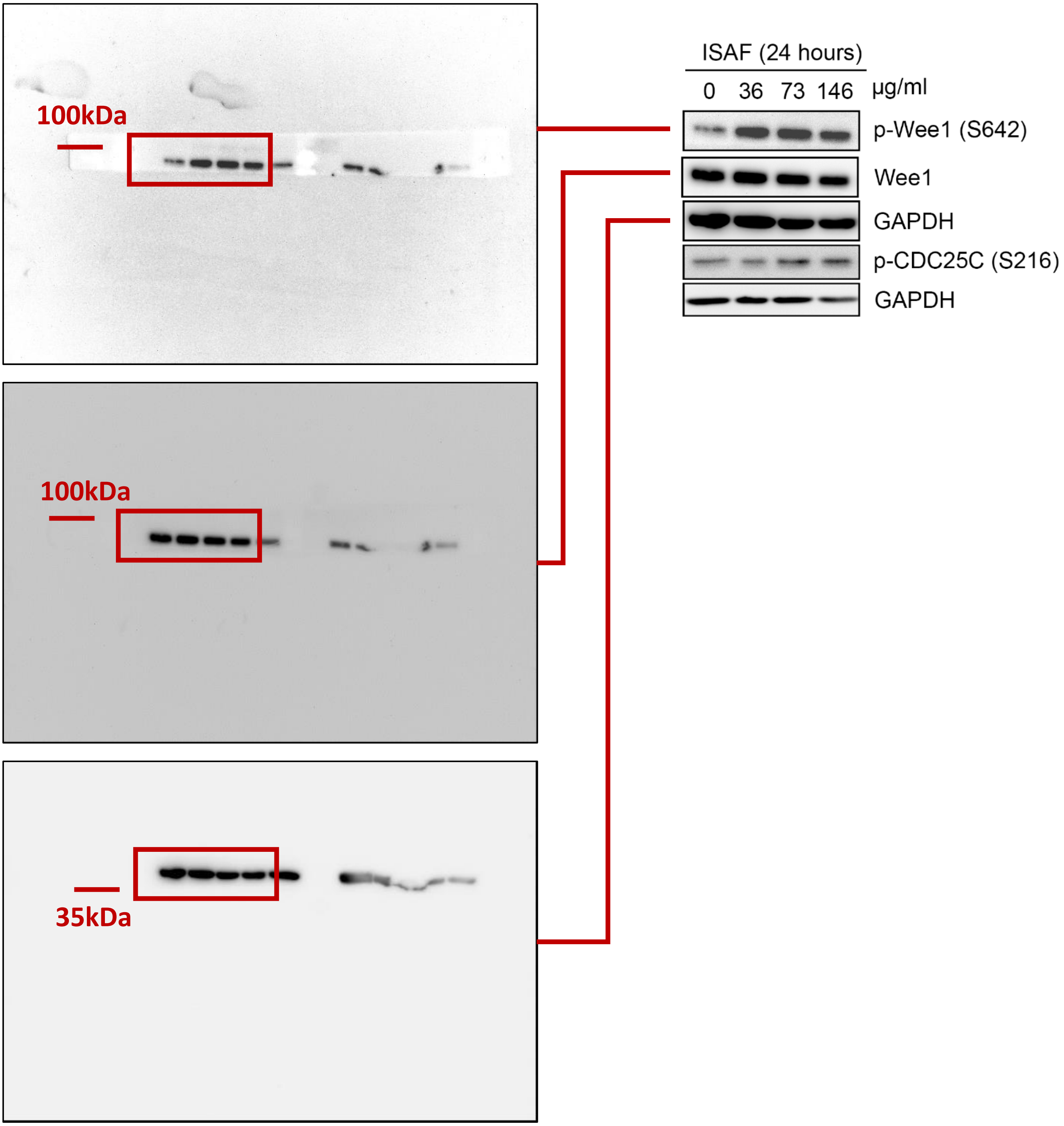

Figure 3B-left panel

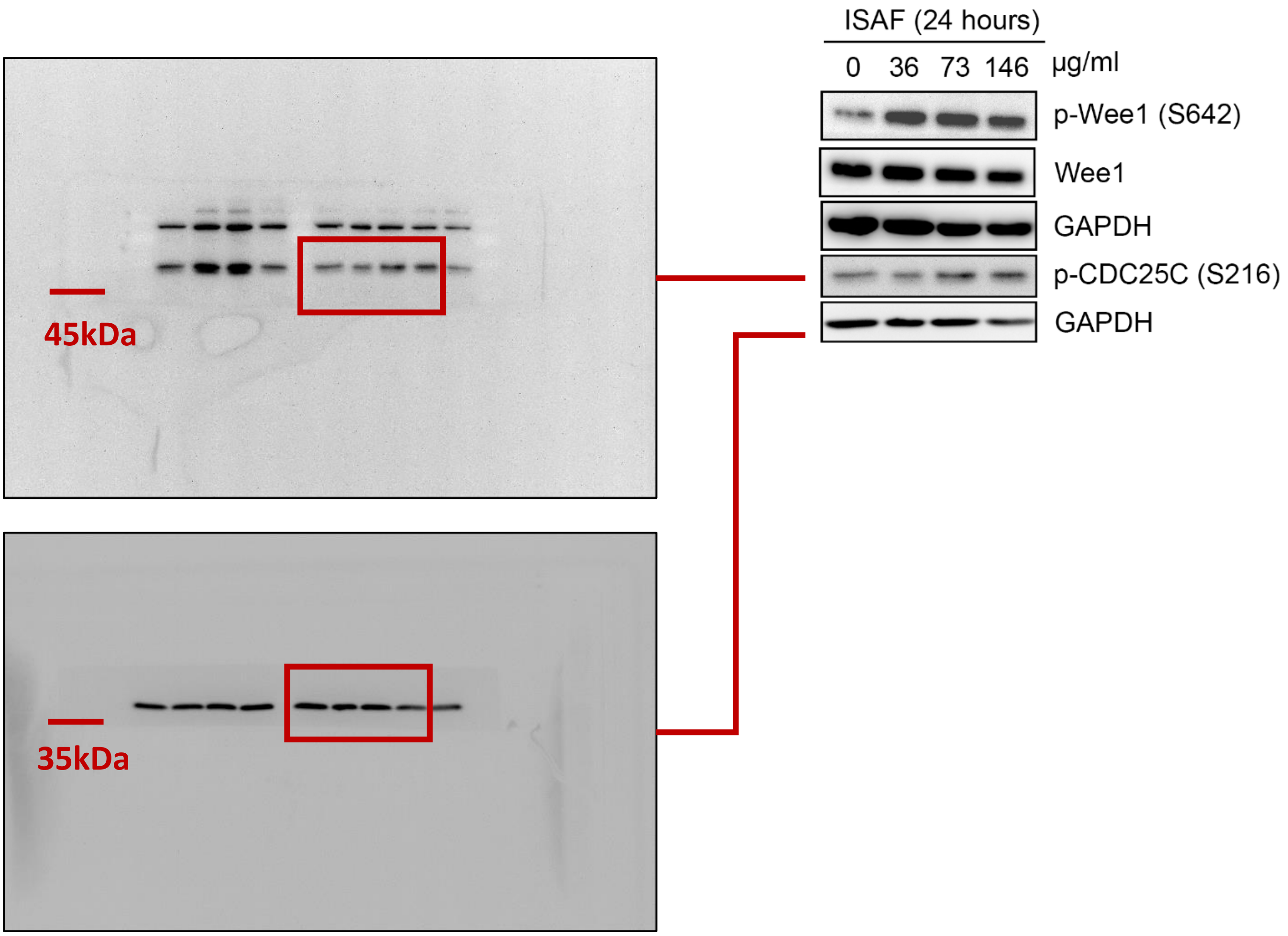

Figure 3B-right panel

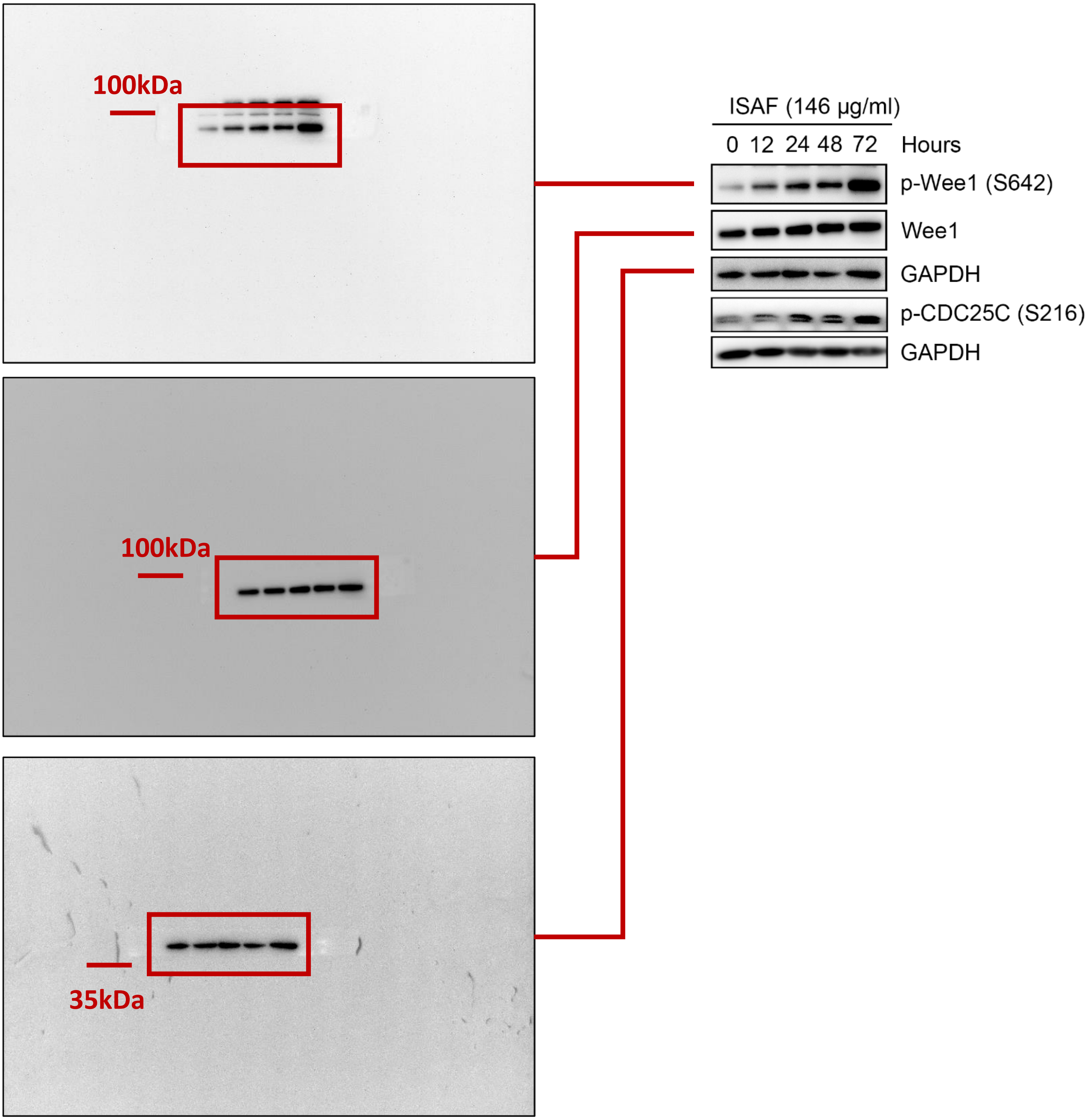

Figure 3B-right panel

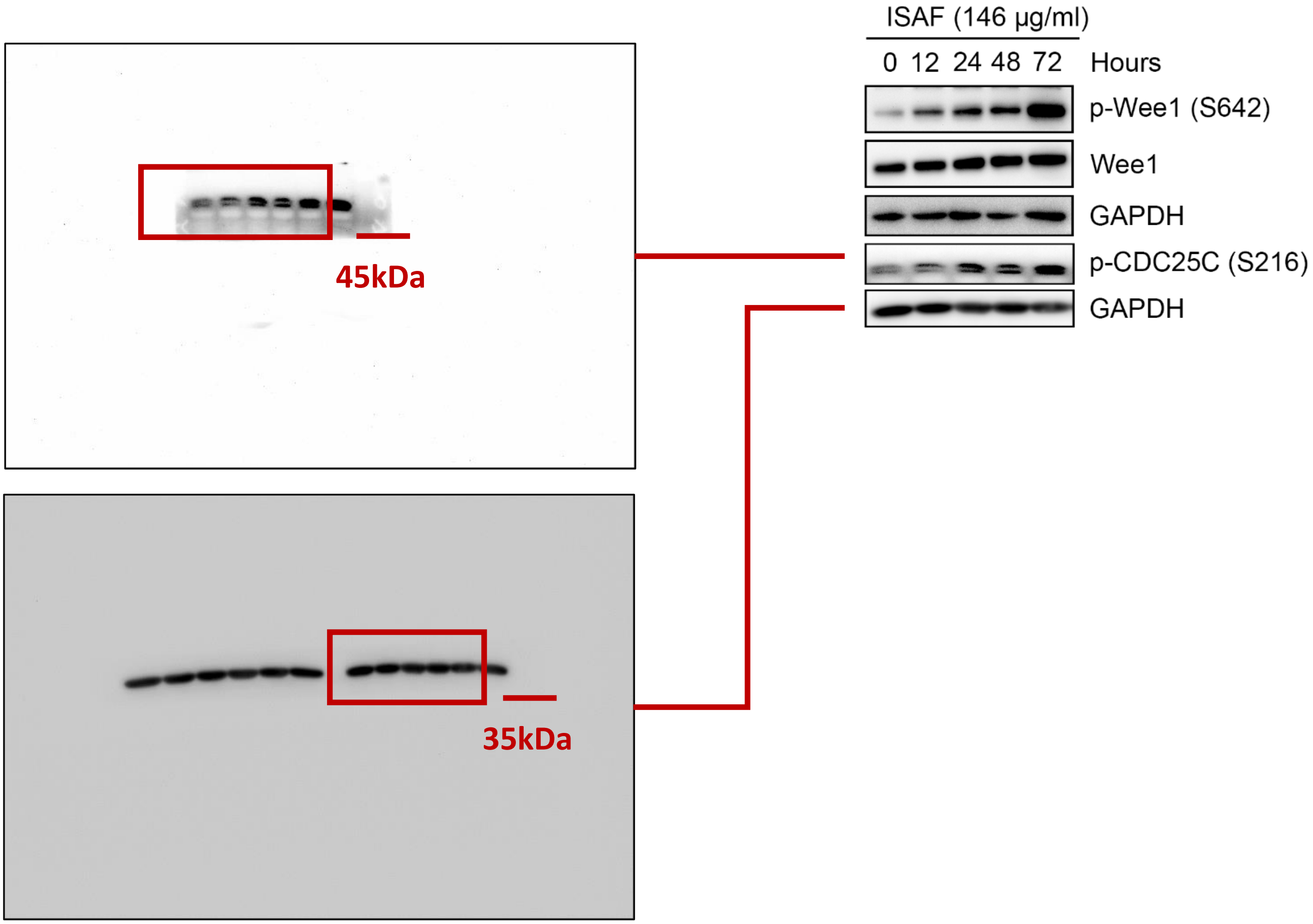

Figure 3C-left panel

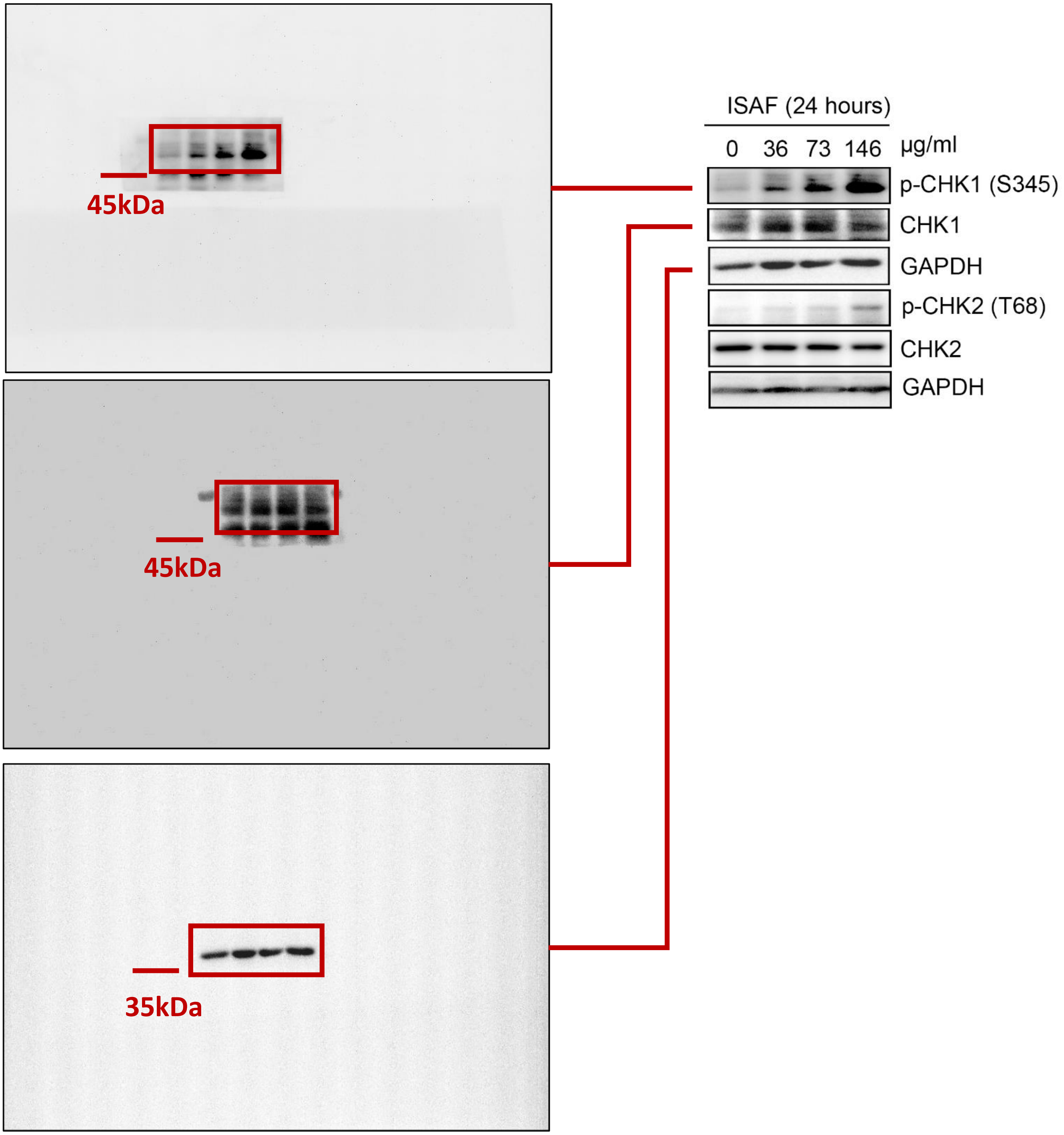

Figure 3C-left panel

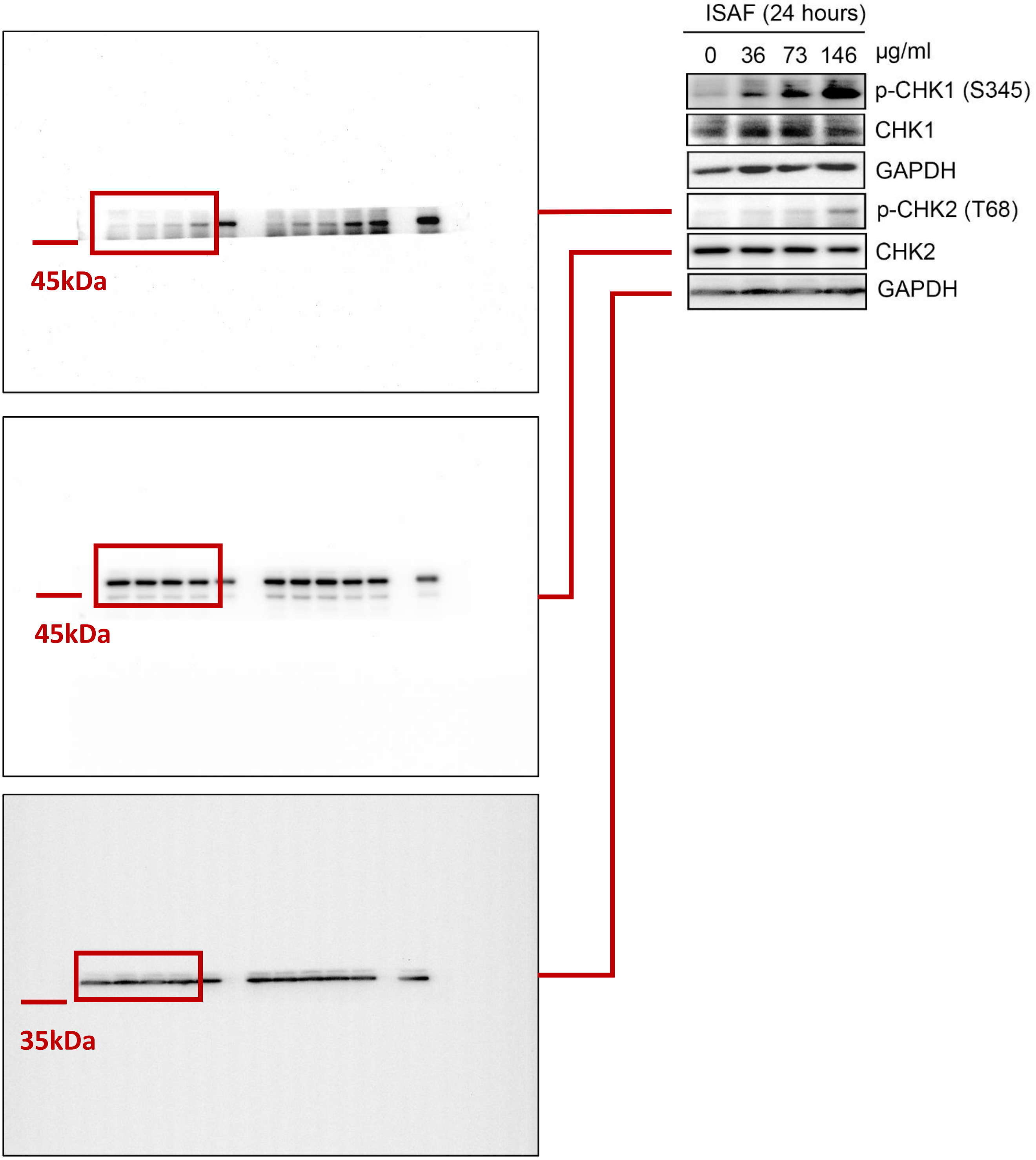

Figure 3C-right panel

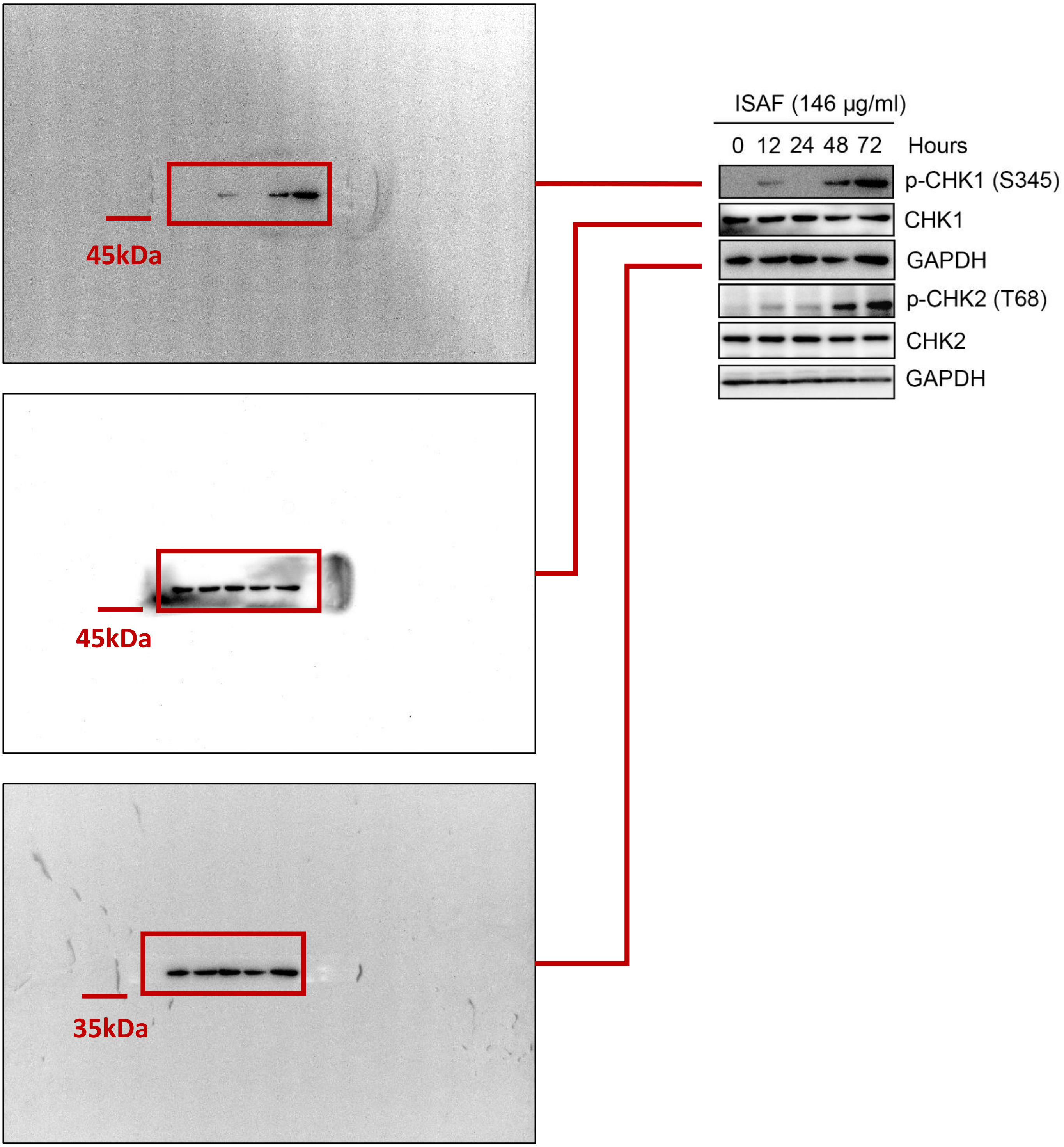

Figure 3C-right panel

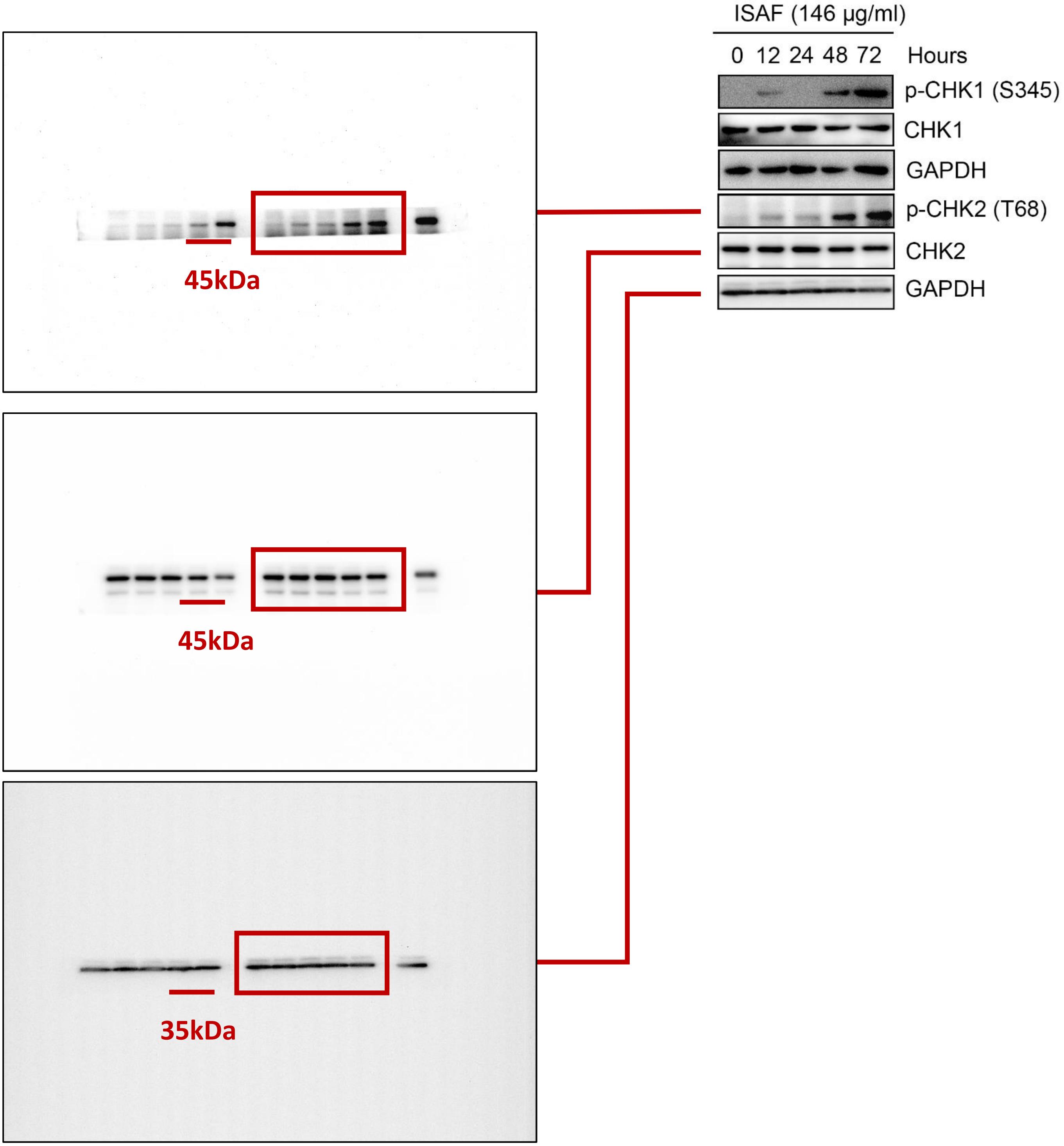

Figure 3D-left panel

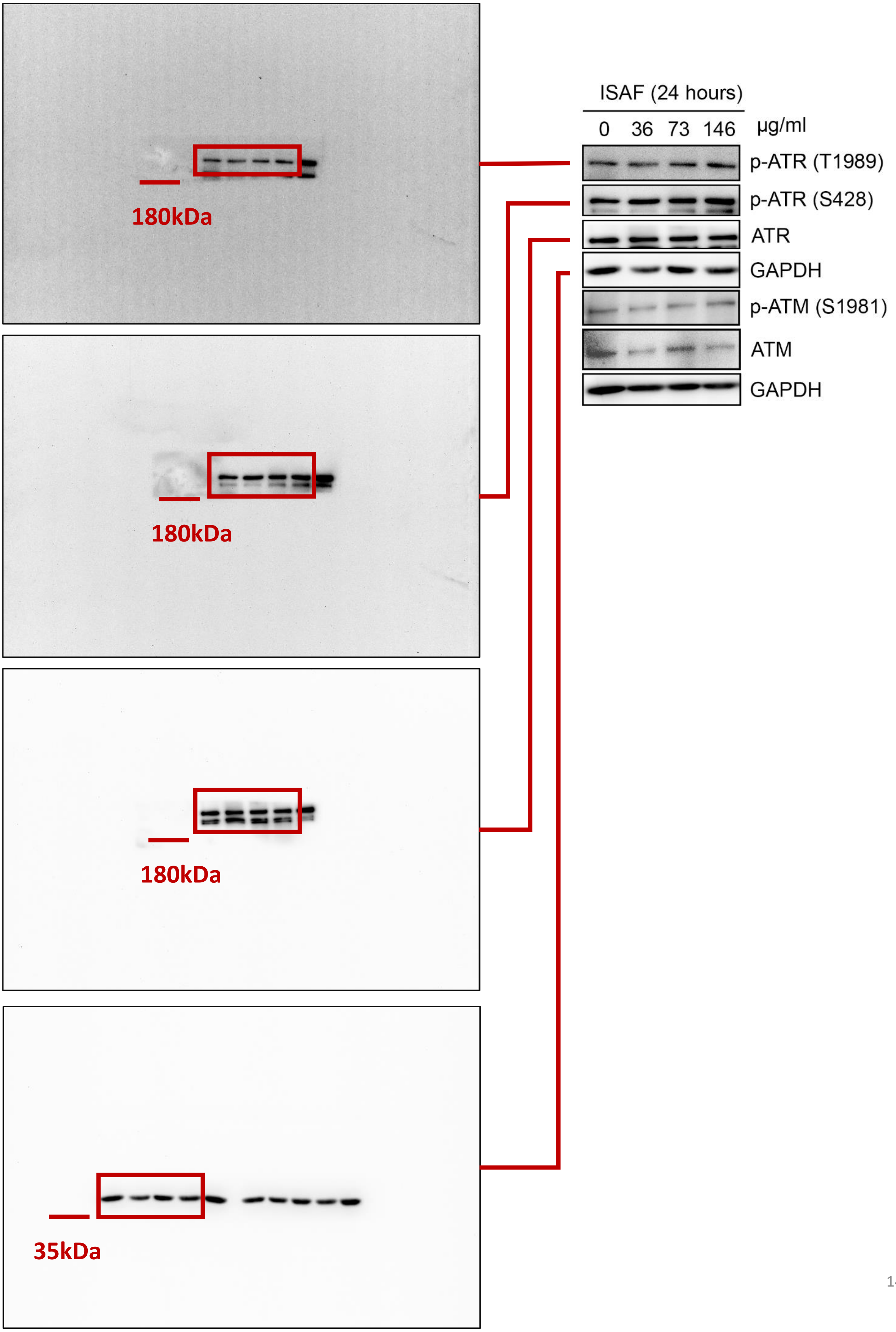

Figure 3D-left panel

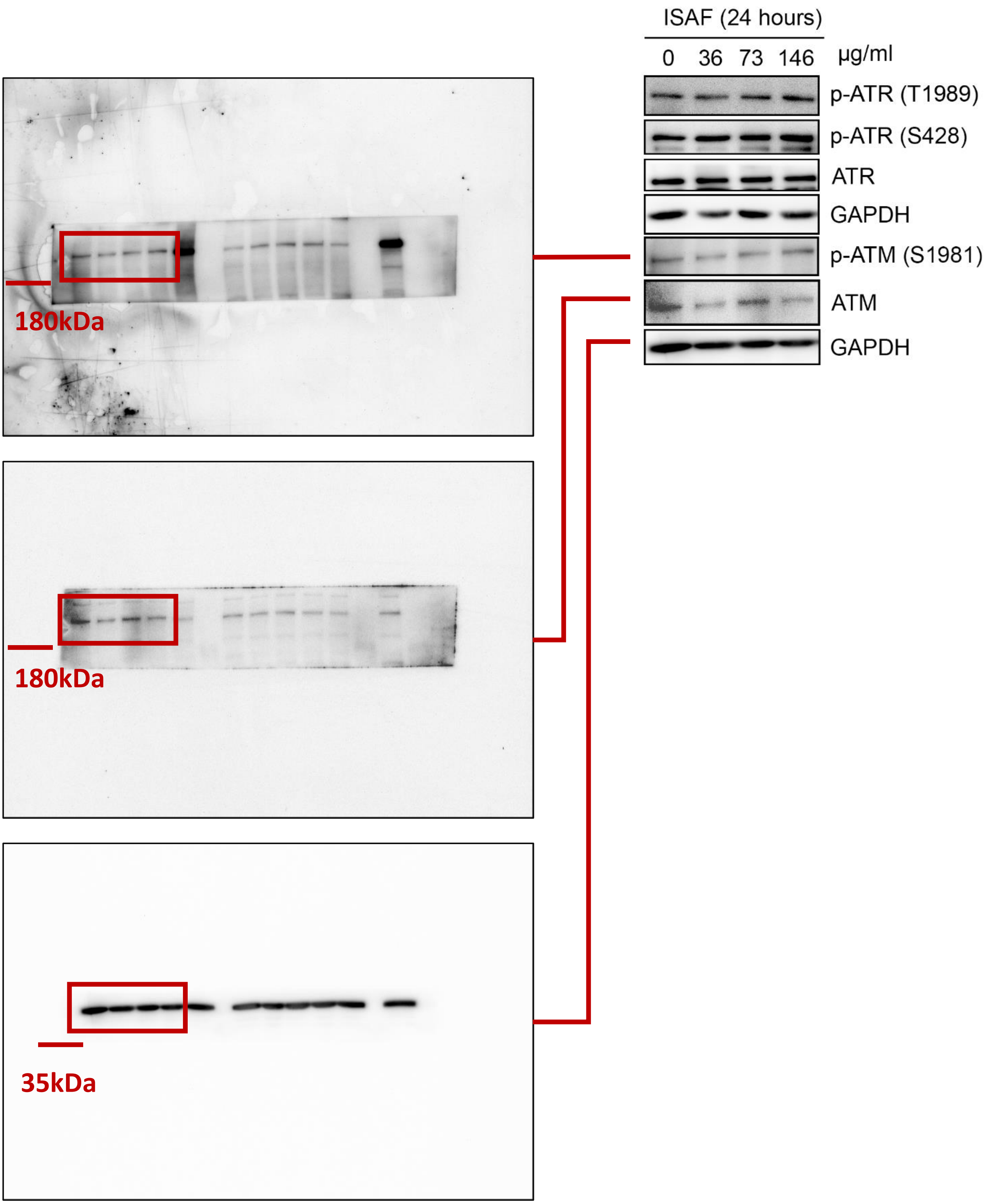

Figure 3D-right panel

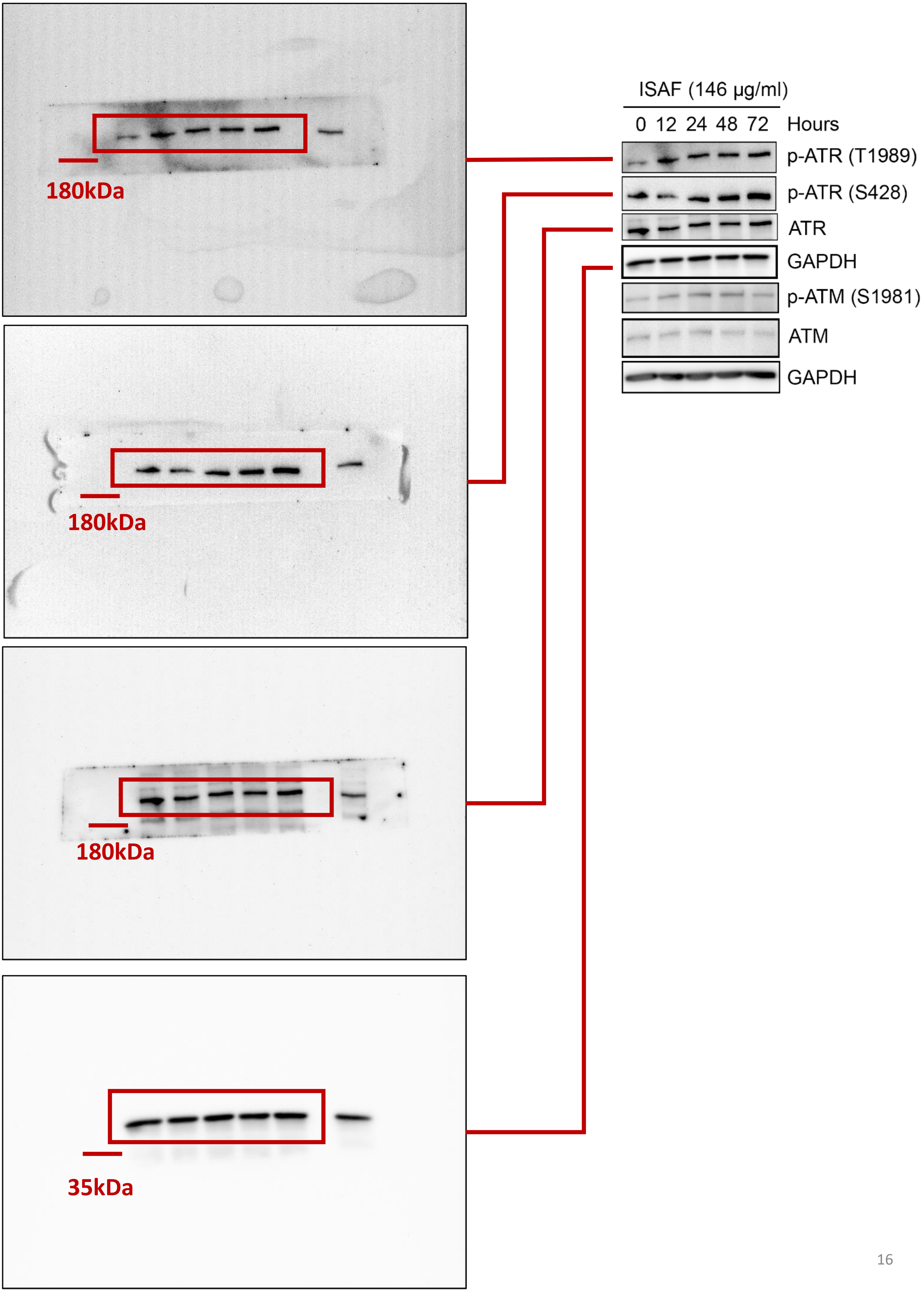

Figure 3D-right panel

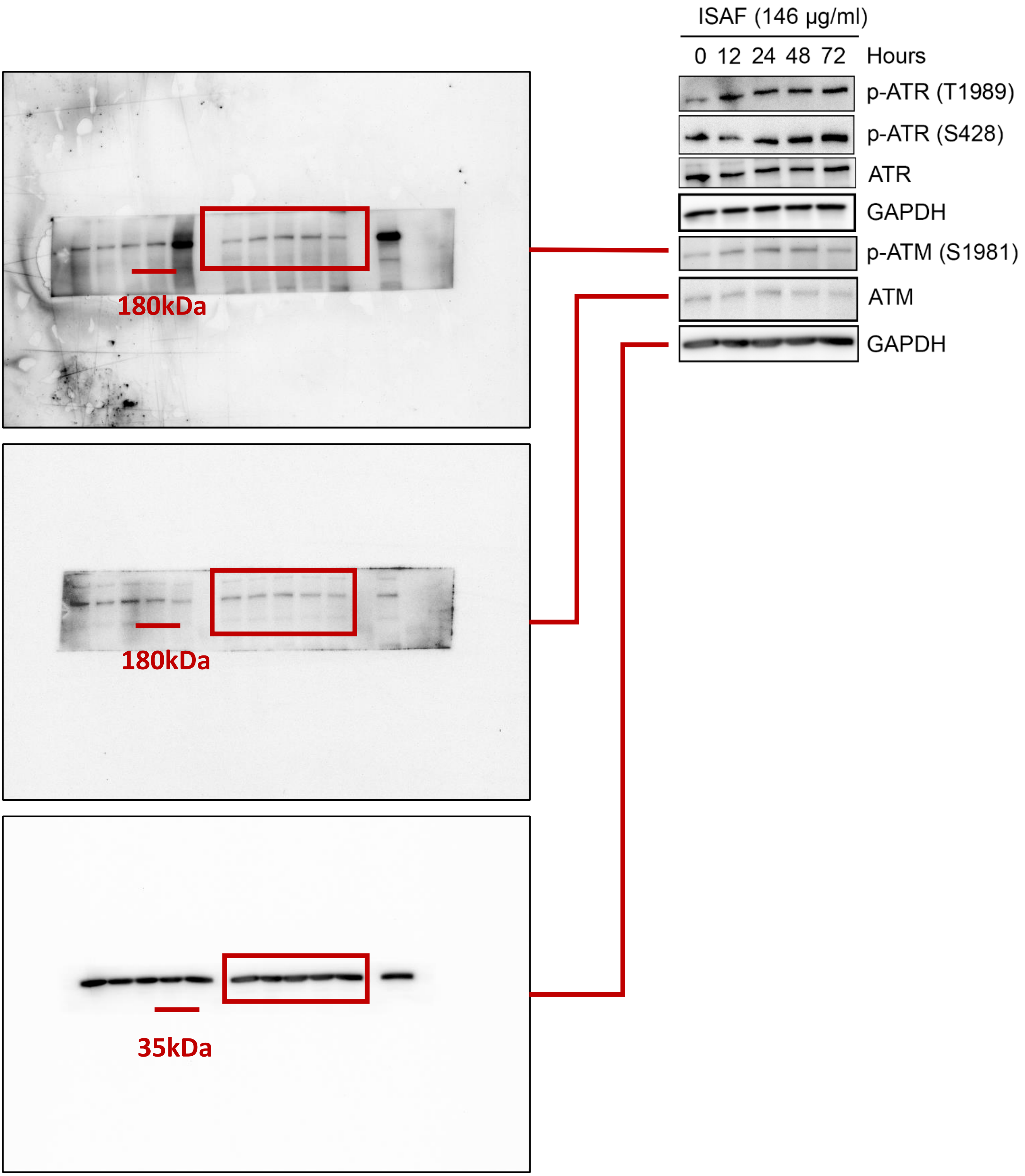

Figure 4C

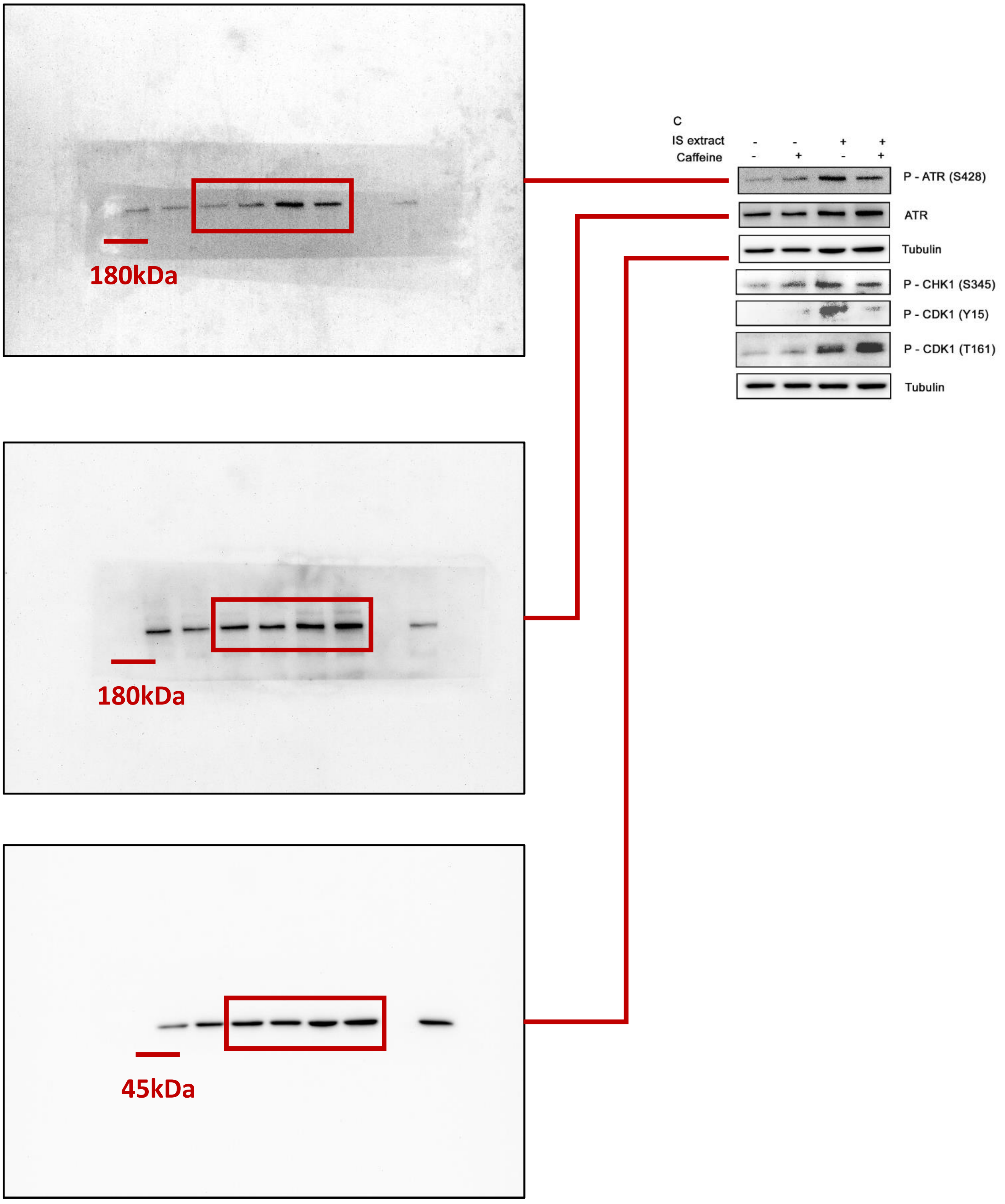
180kDa
180kDa
45kDa
C
IS extract
Caffeine
- - + +
- + - +
P - ATR (S428)
ATR
Tubulin
P - CHK1 (S345)
P - CDK1 (Y15)
P - CDK1 (T161)
Tubulin

Figure 4C

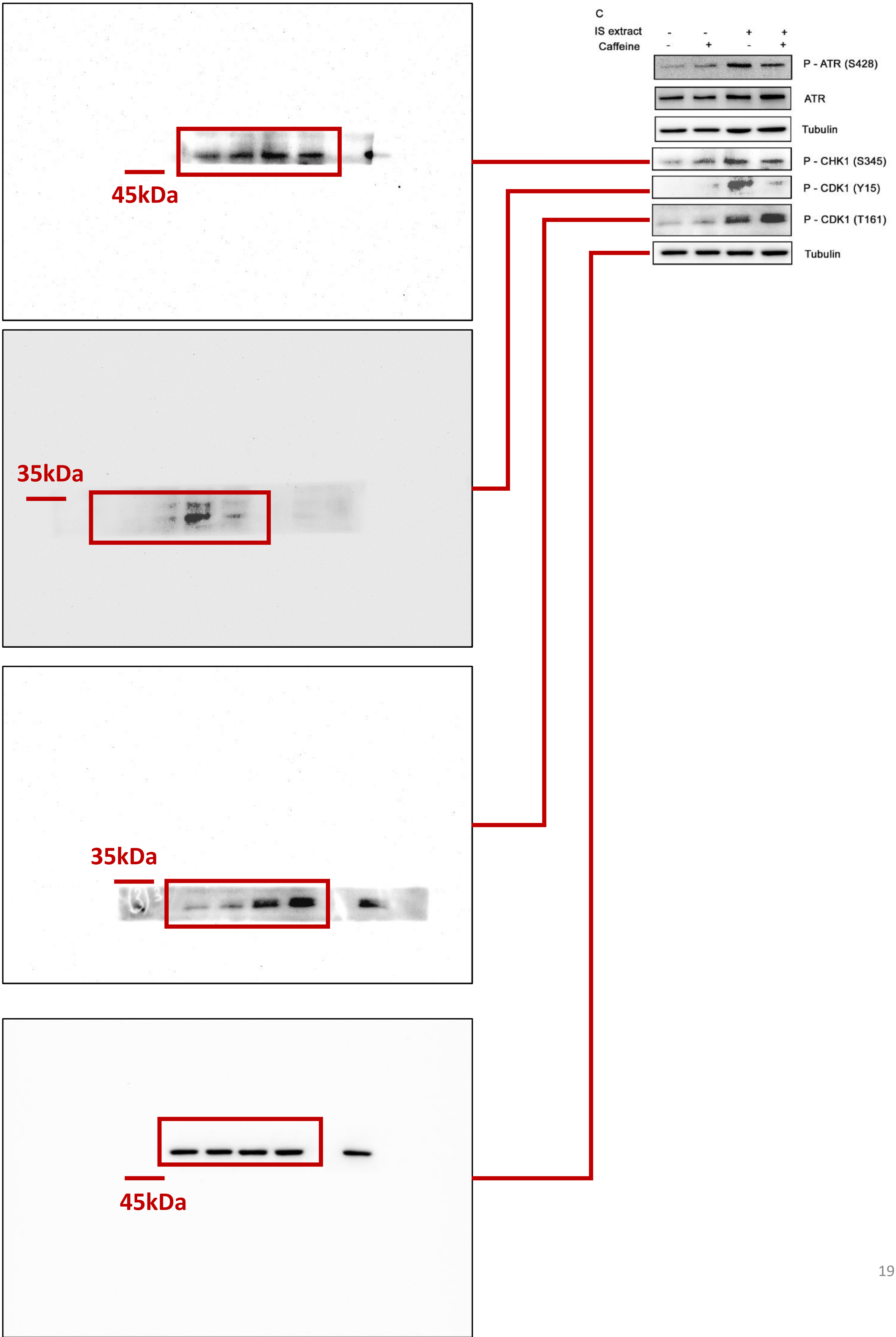

45kDa
35kDa
35kDa
45kDa
C
IS extract - - + +
Caffeine - + - +
P - ATR (S428)
ATR
Tubulin
P - CHK1 (S345)
P - CDK1 (Y15)
P - CDK1 (T161)
Tubulin

Multiple exposure images of WB

Figure 3A-dose-manner

1

*: Presented photo

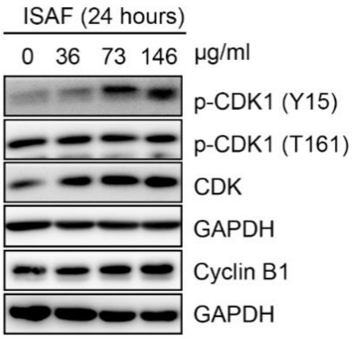

Increasing exposure time

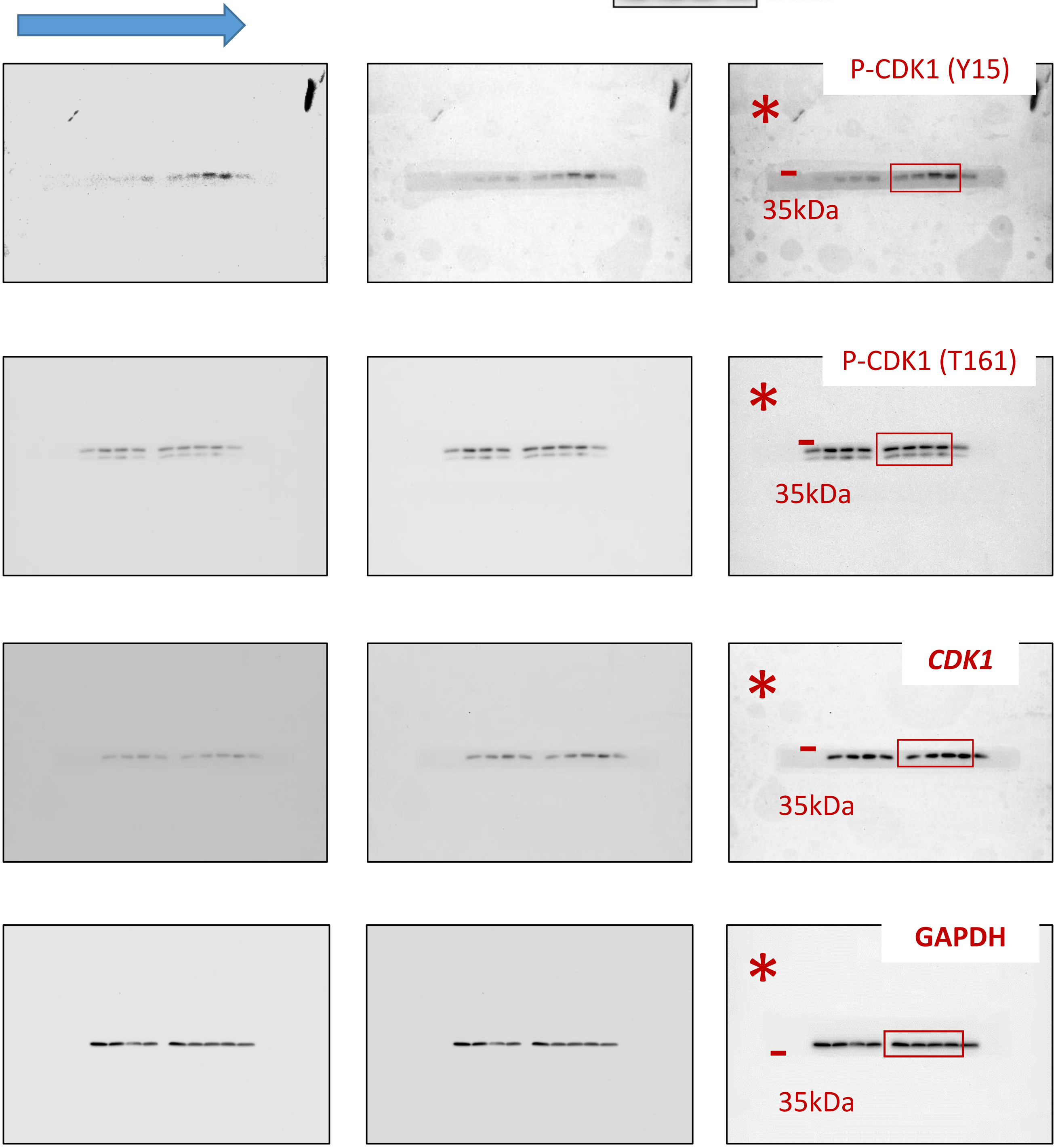

Figure 3A-dose manner

*: Presented photo

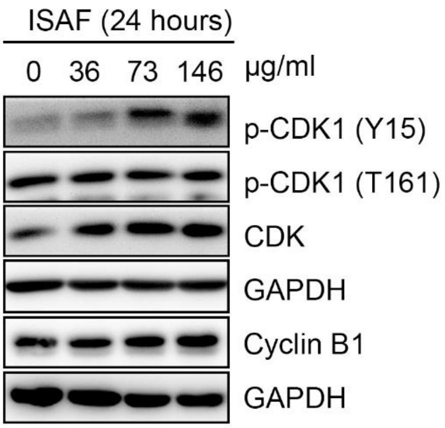

Increasing exposure time

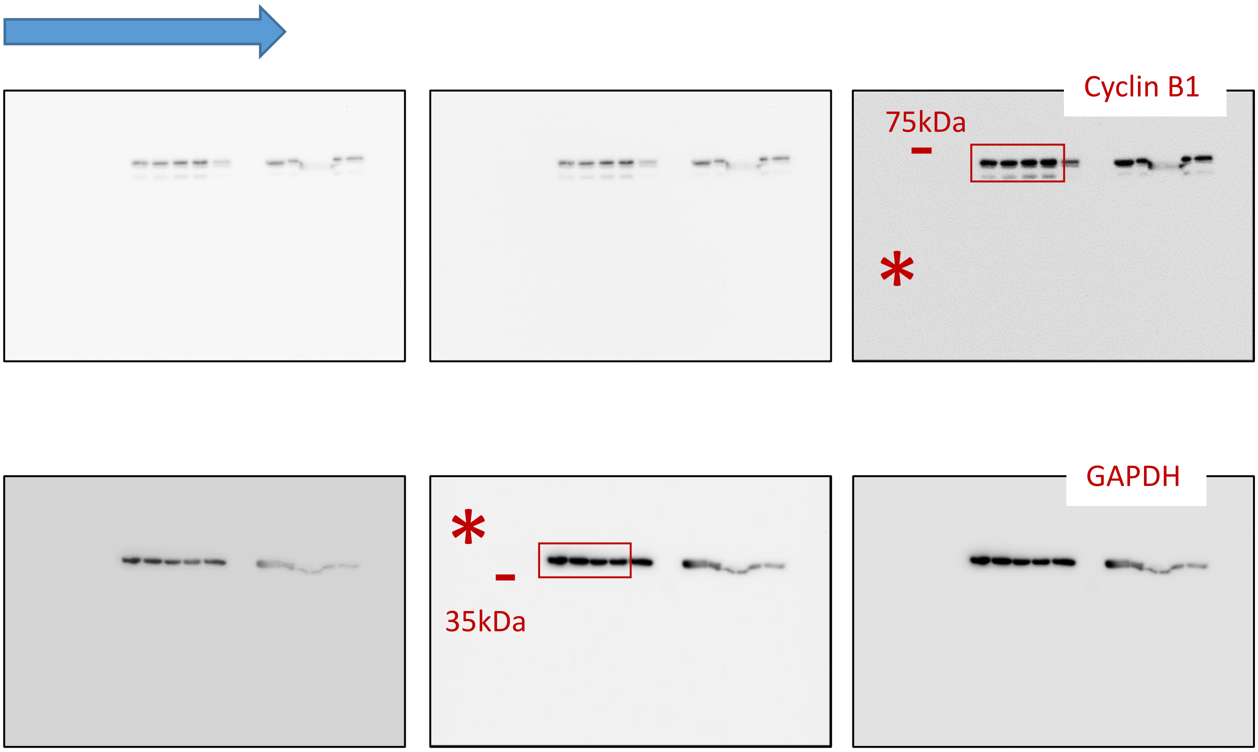

Figure 3A time manner

*: Presented photo

Increasing exposure time

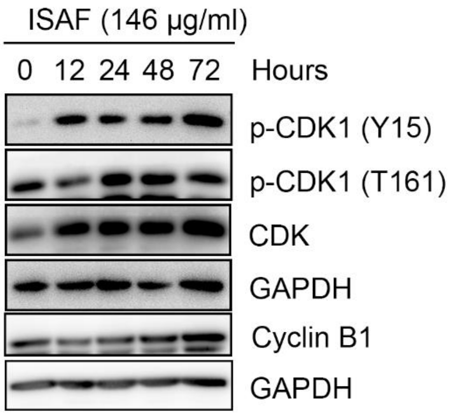

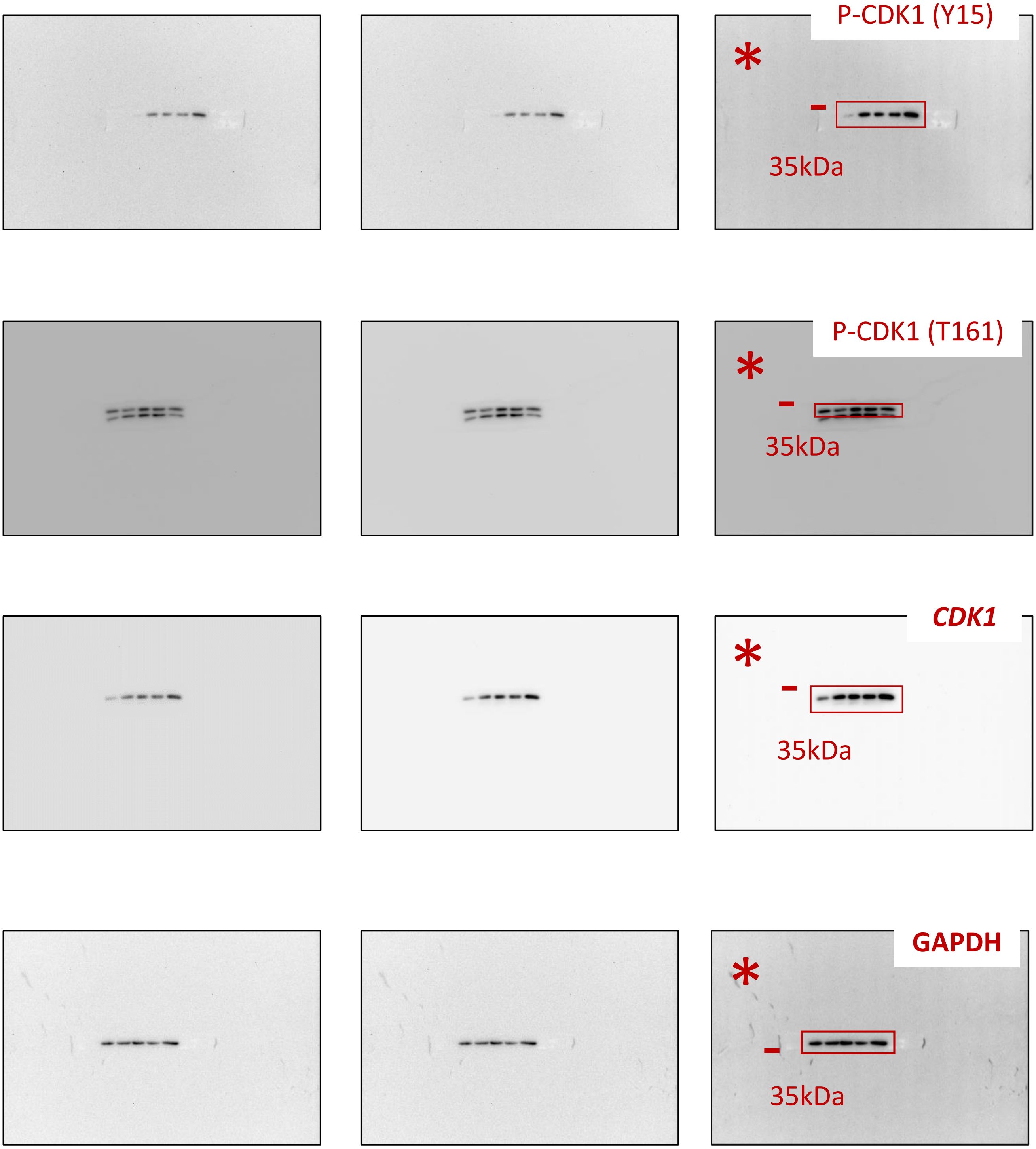

Figure 3A-right panel

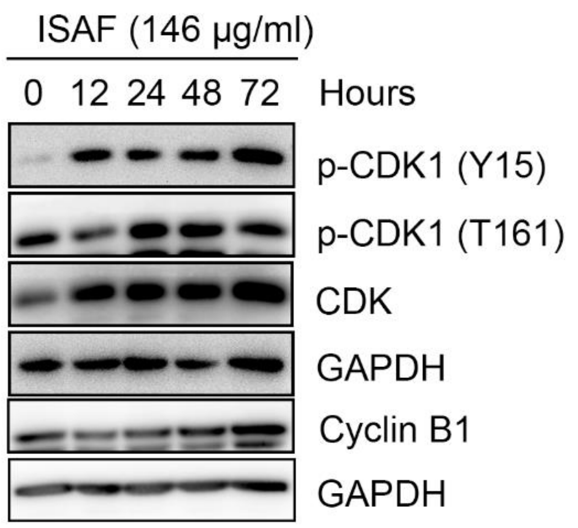

*: Presented photo

Increasing exposure time

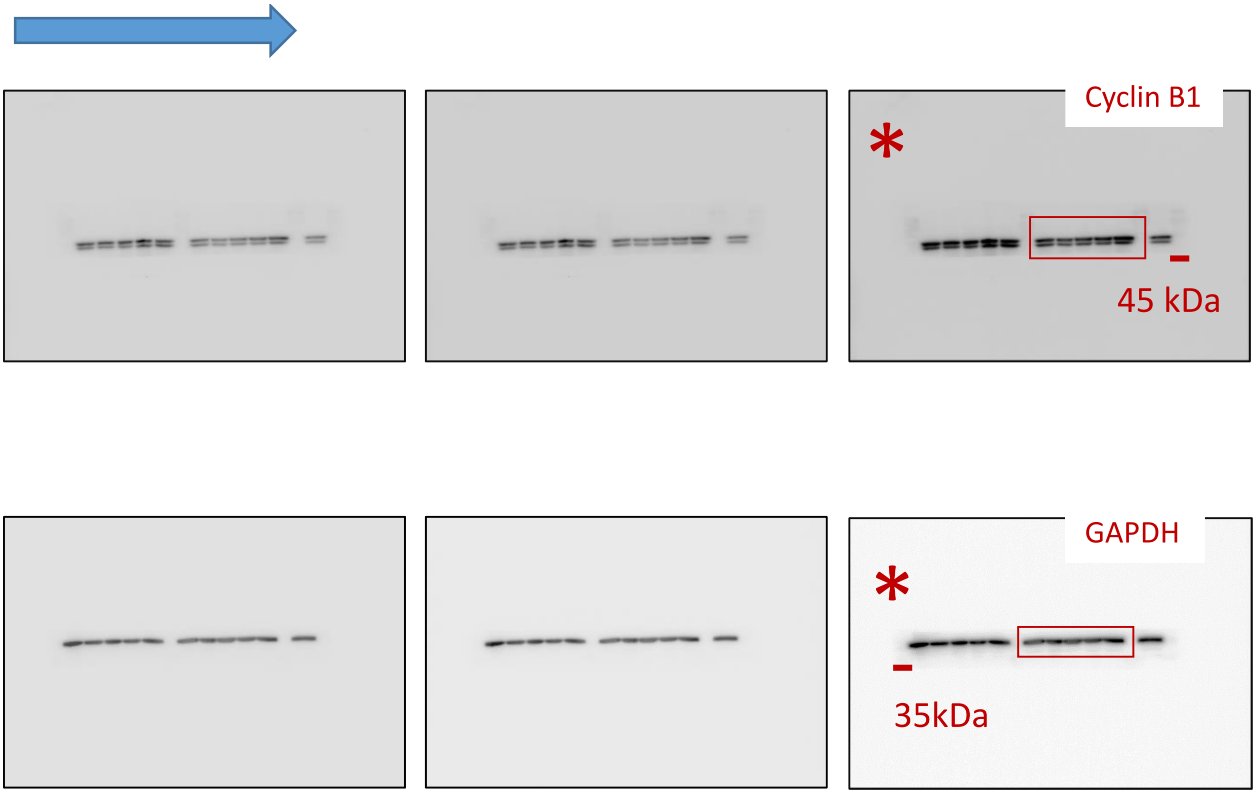

Figure 3B – dose manner

*: Presented photo

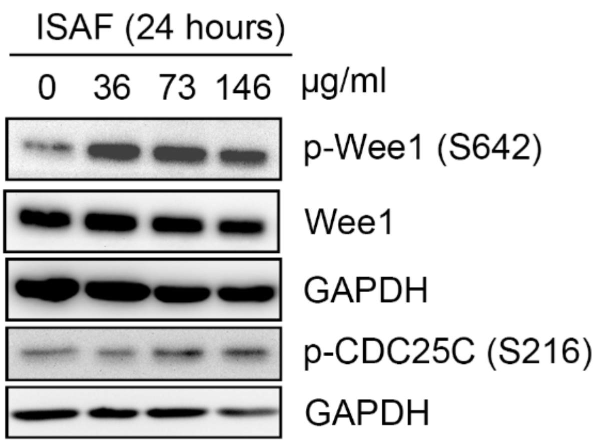

Increasing exposure time

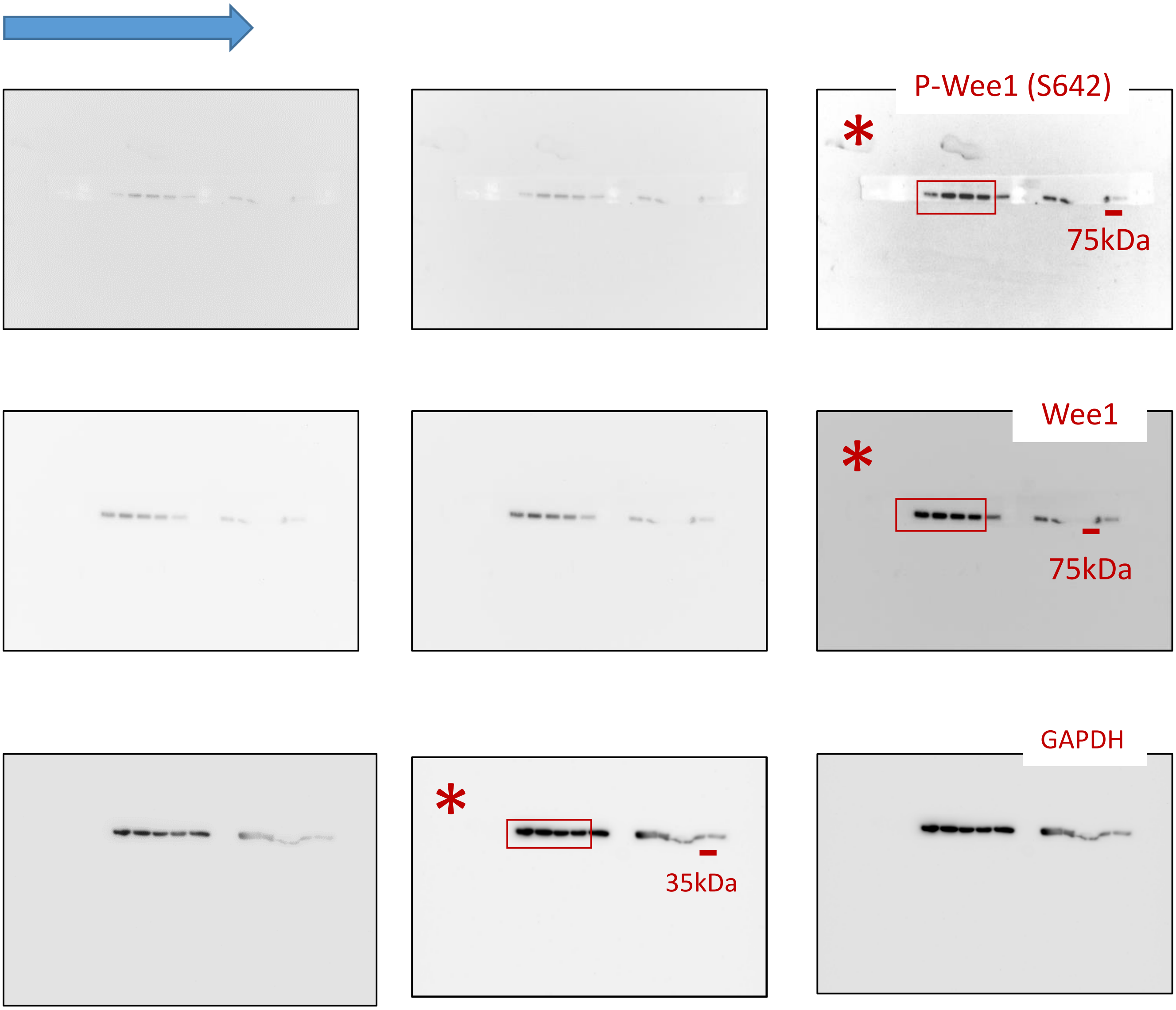

Figure 3B - dose manner

*: Presented photo

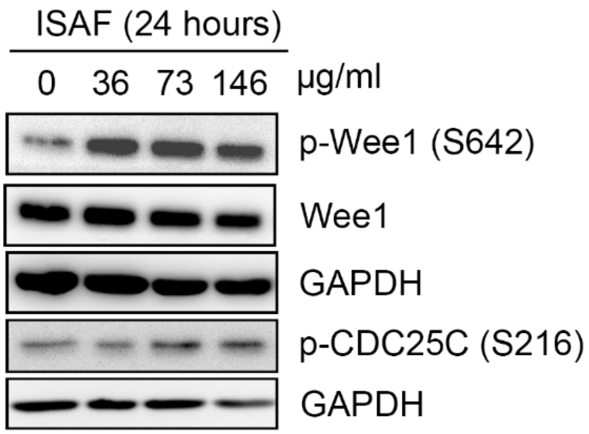

Increasing exposure time

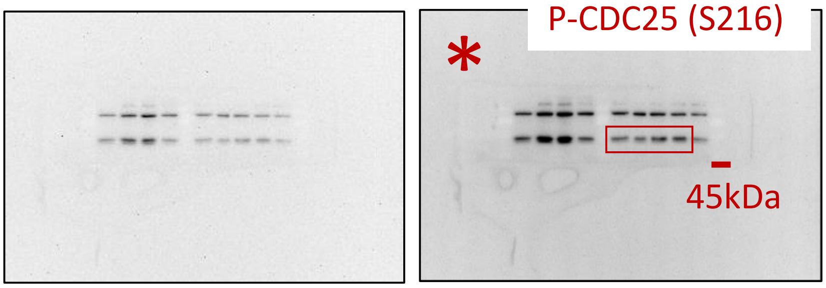

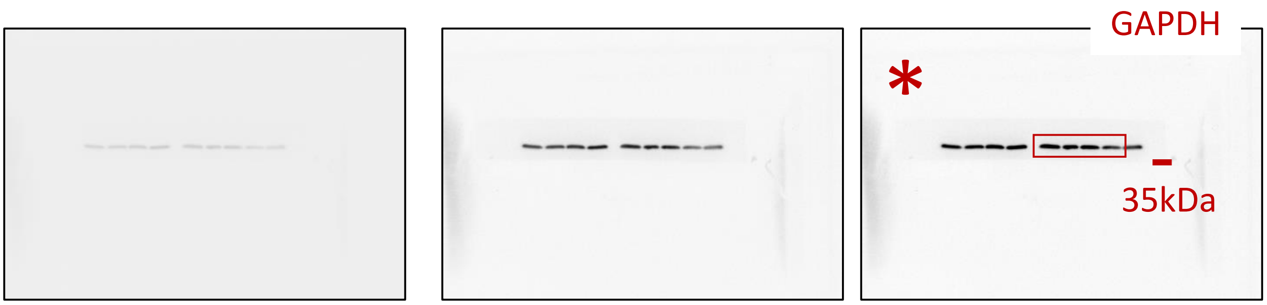

Figure 3B-right panel

*: Presented photo

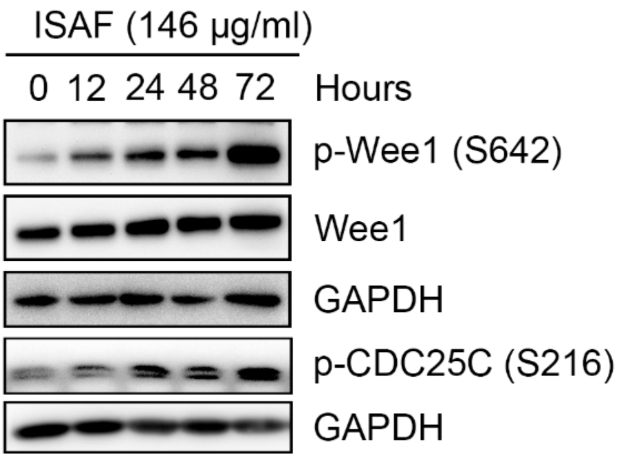

Increasing exposure time

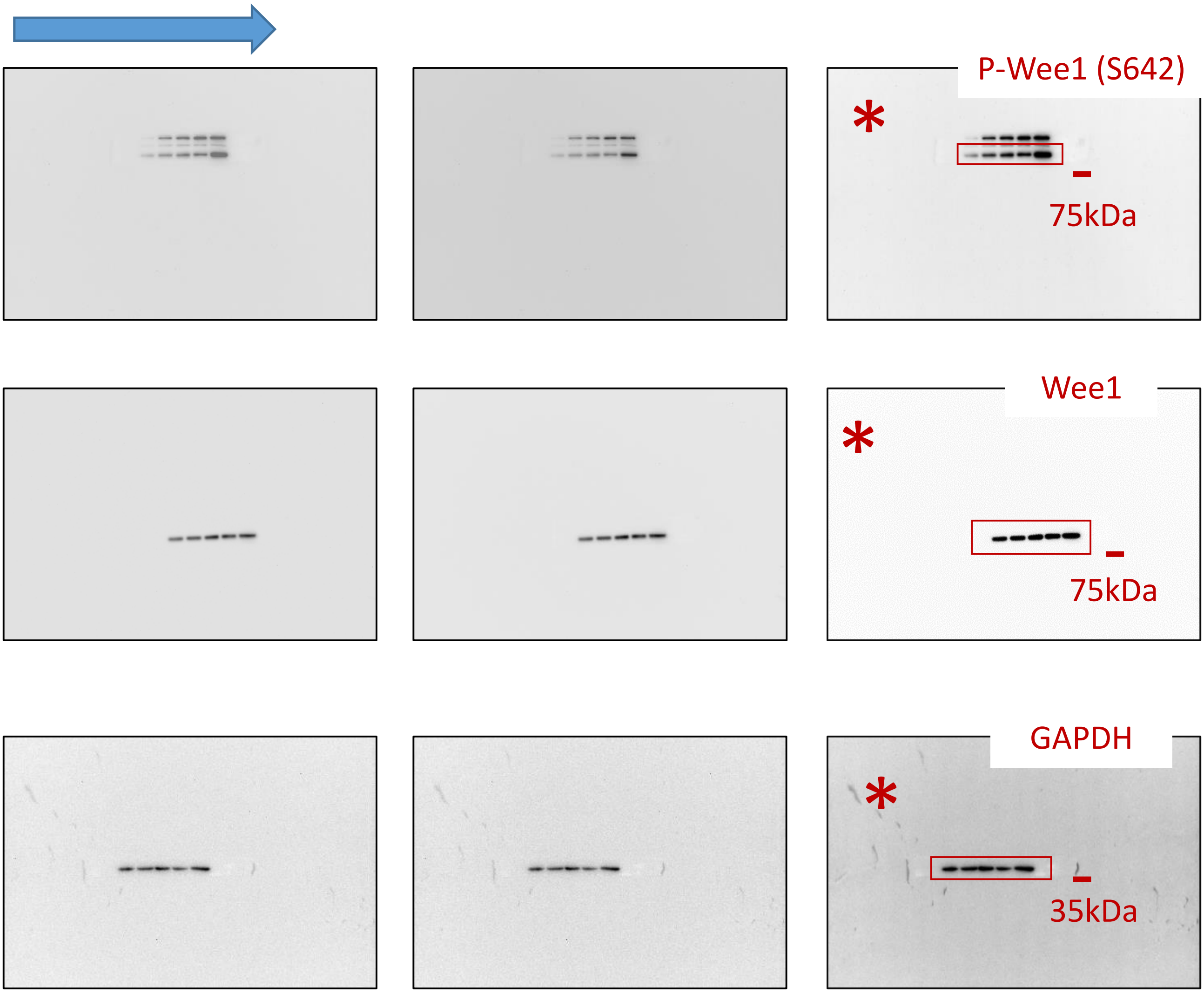

Figure 3B time manner

*: Presented photo

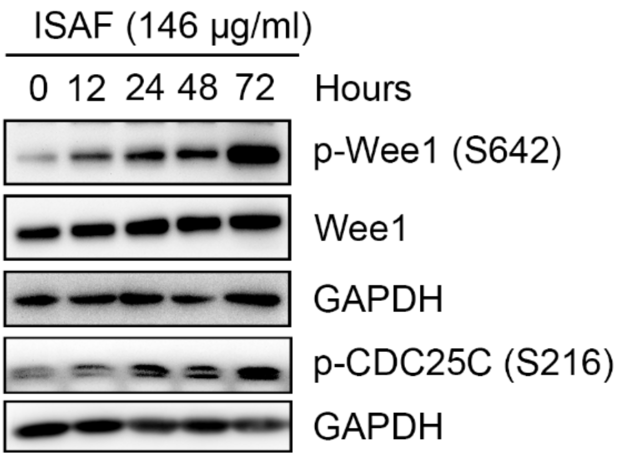

Increasing exposure time

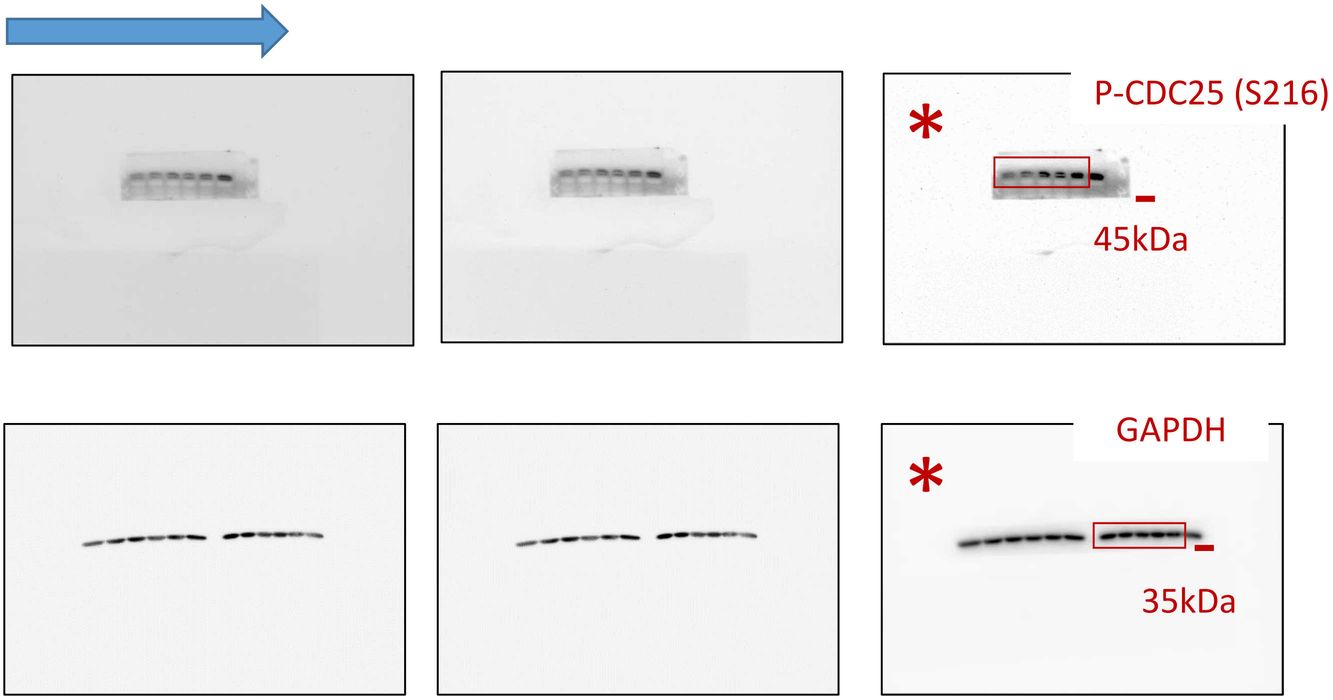

Figure 3C – dose manners

*: Presented photo

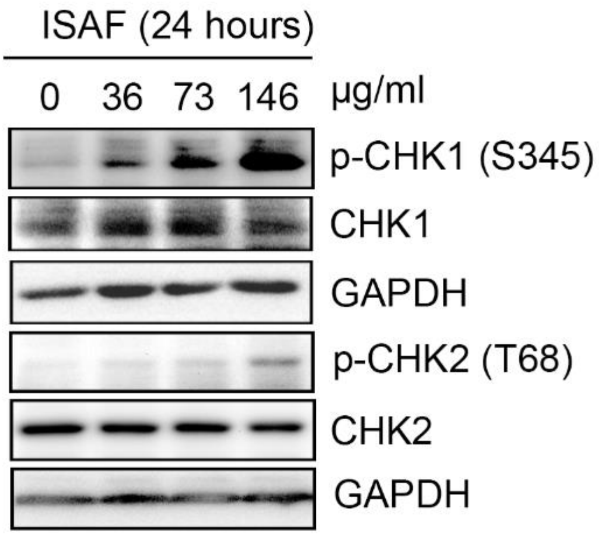

Increasing exposure time

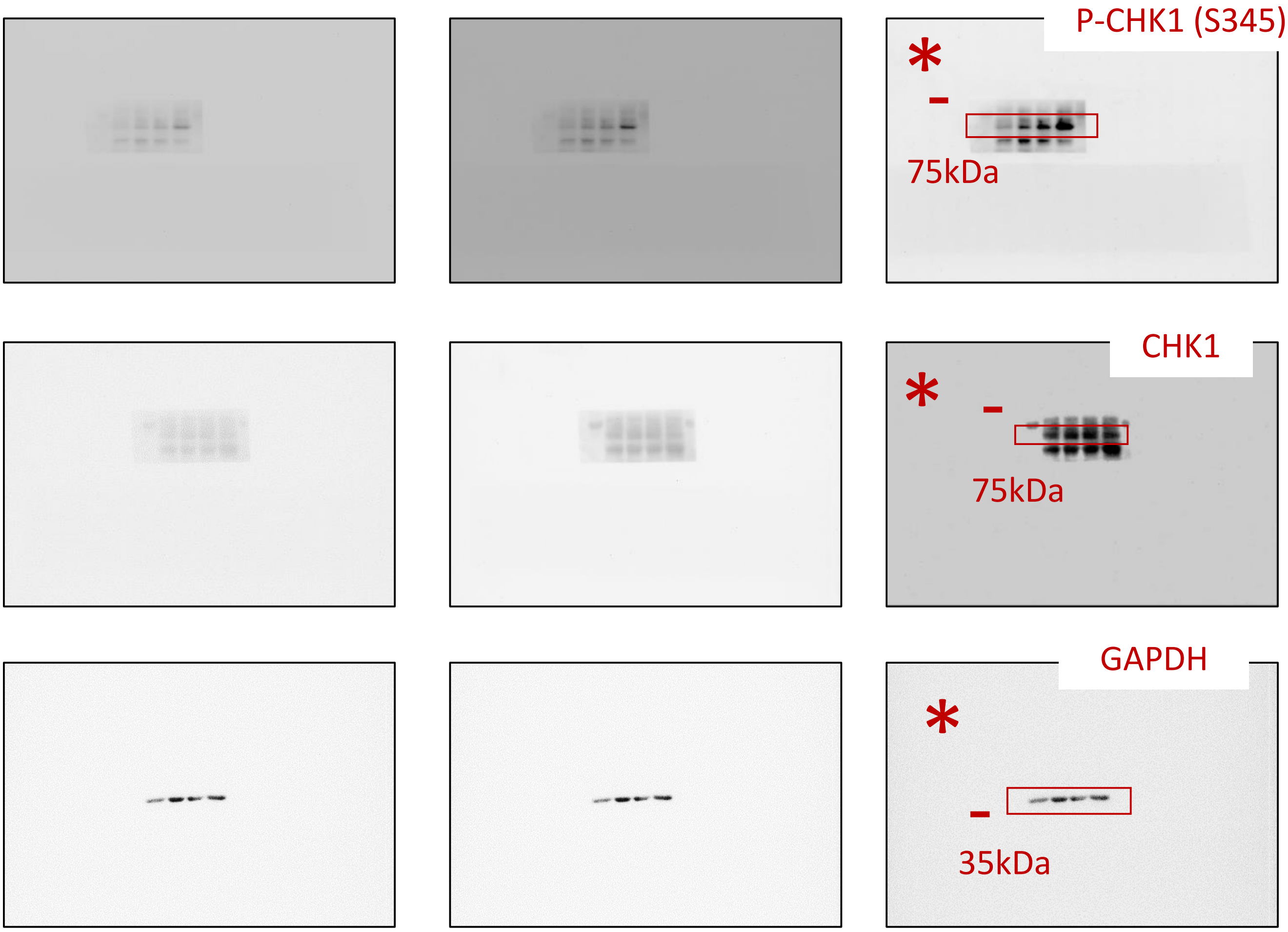

Figure 3C – dose manners

*: Presented photo

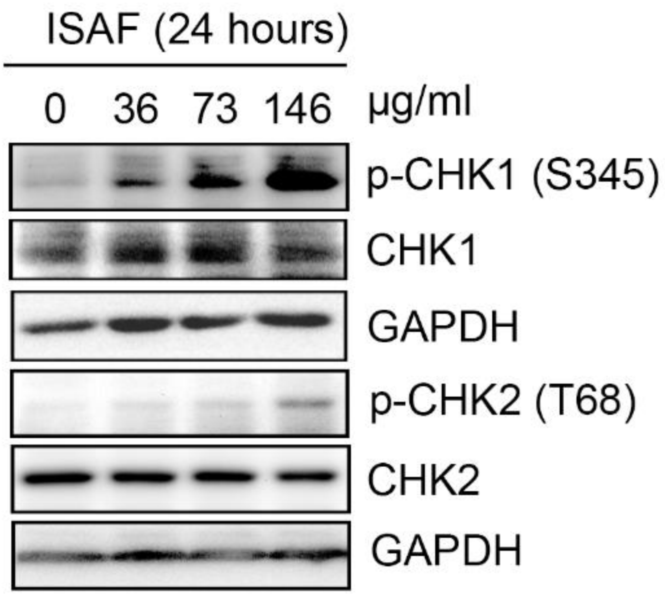

Increasing exposure time

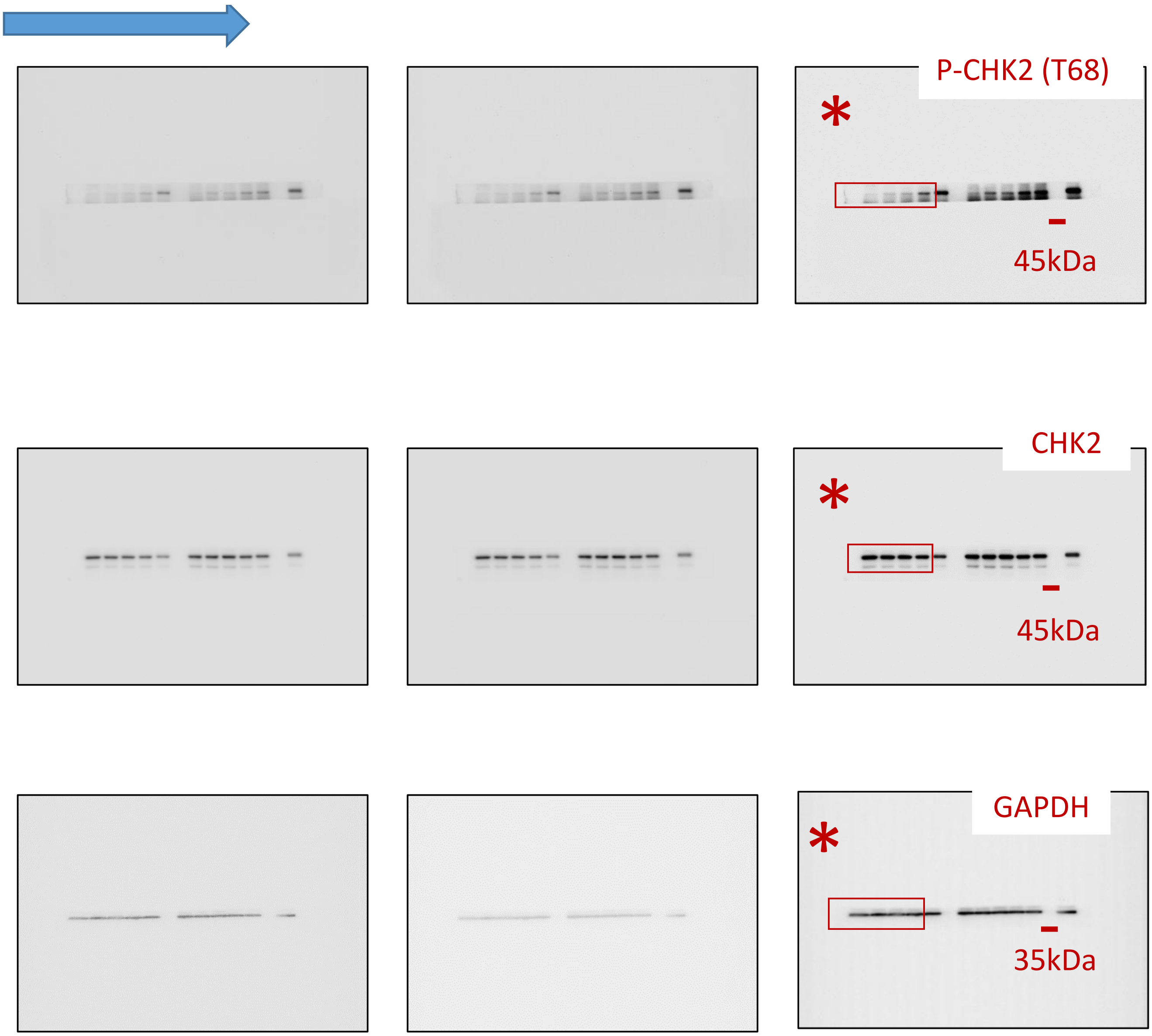

Figure 3C- time manner

*: Presented photo

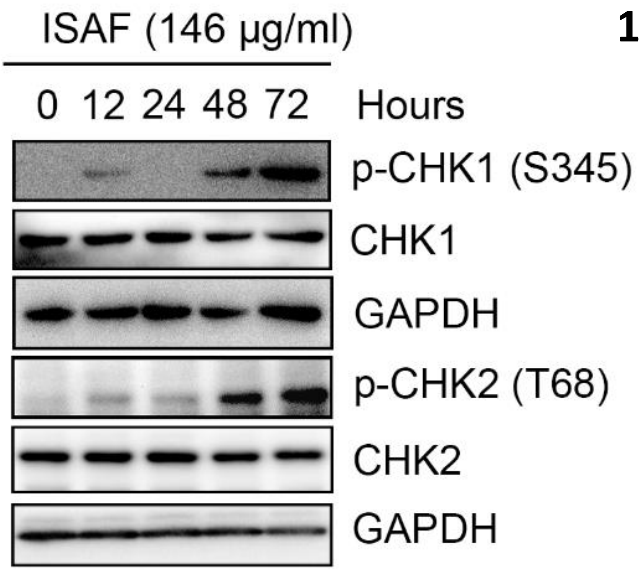

Increasing exposure time

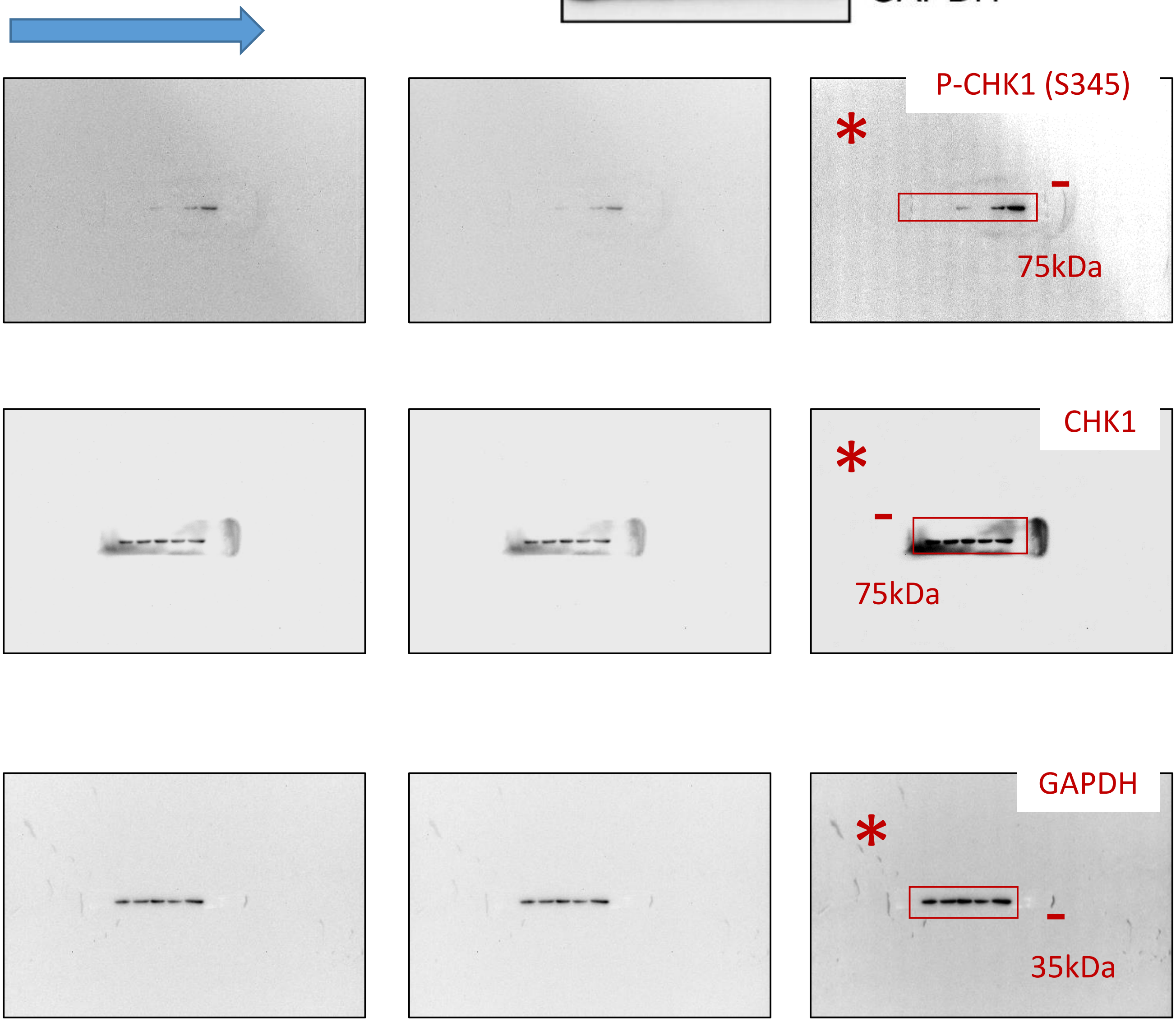

Figure 3C – dose manner

*: Presented photo

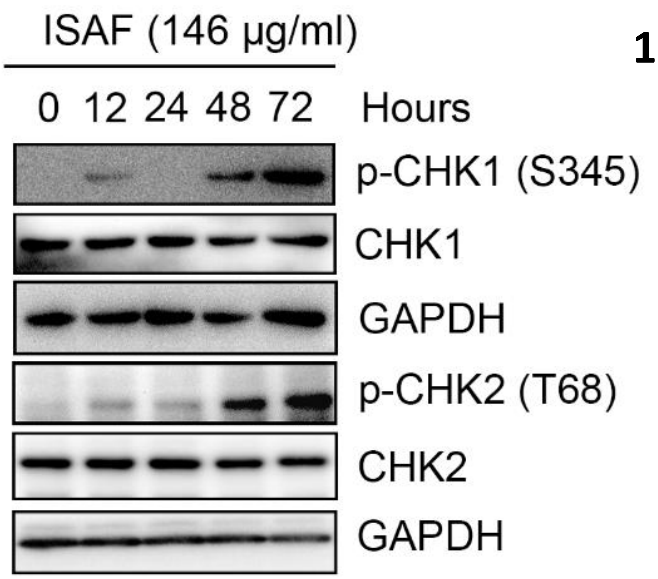

Increasing exposure time

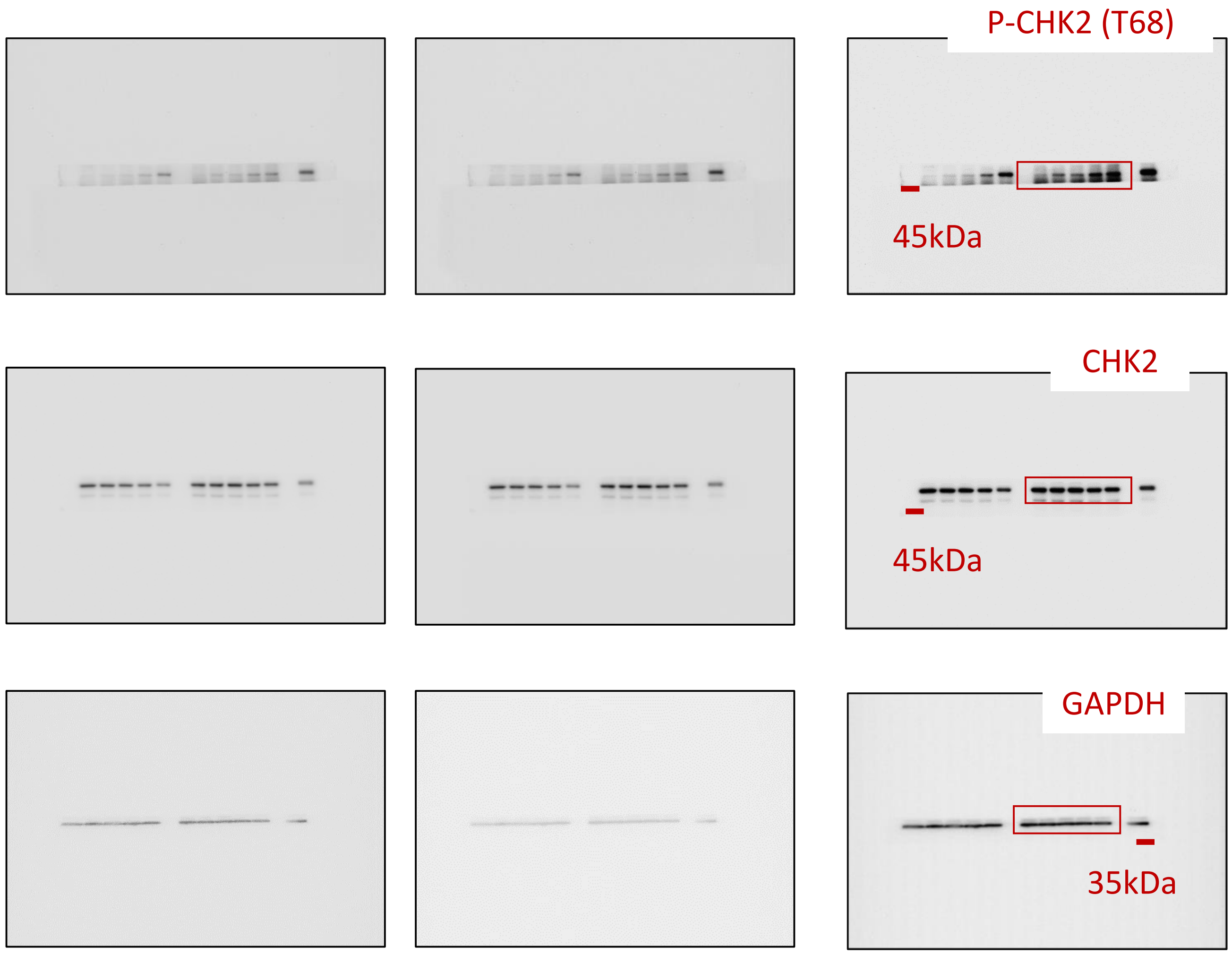

Figure 3D – dose manner

*: Presented photo

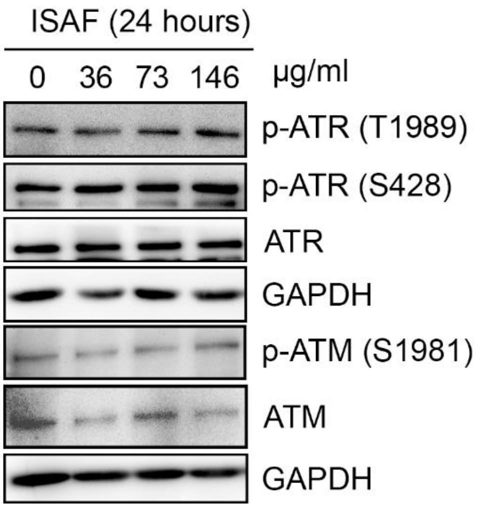

Increasing exposure time

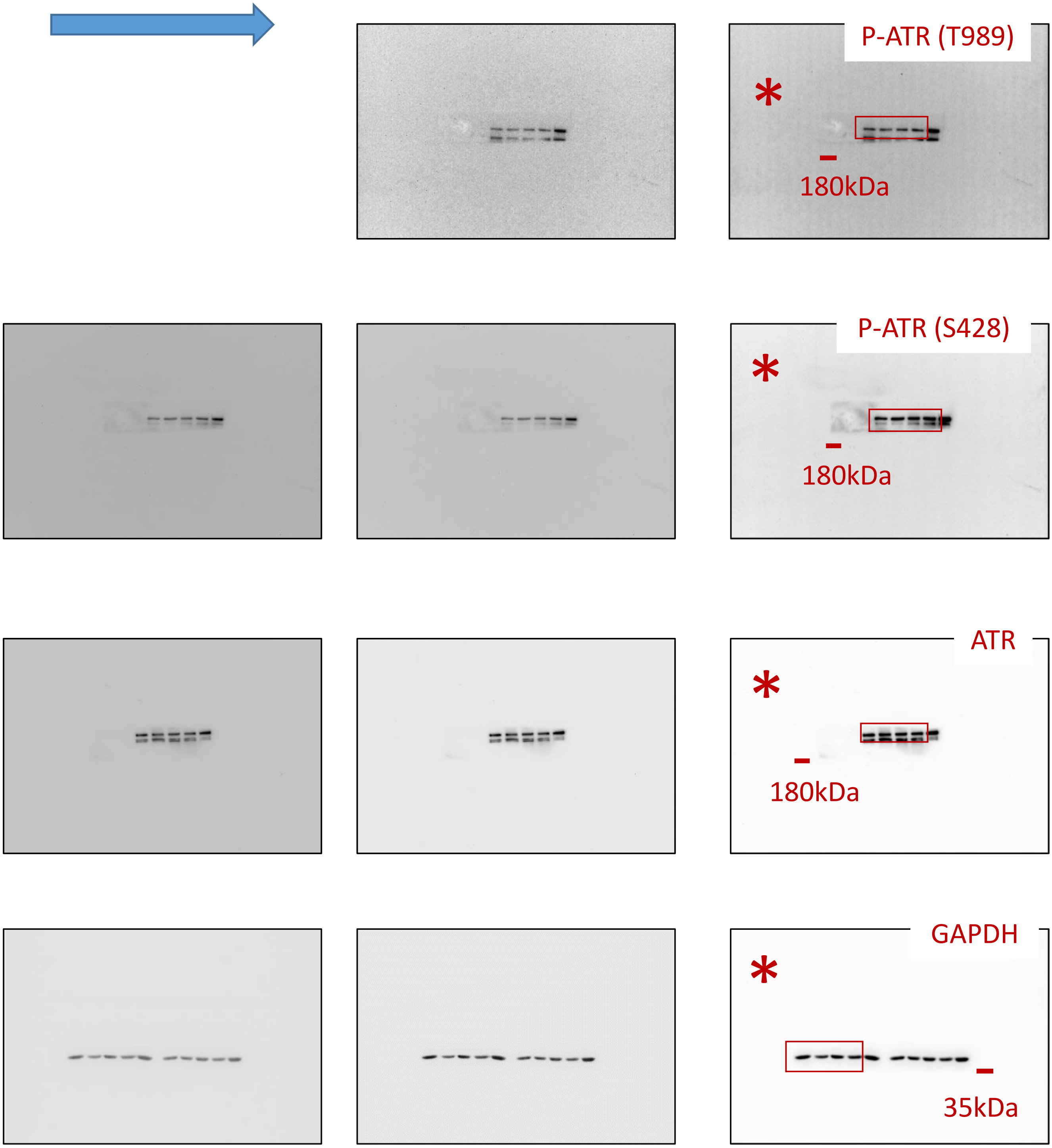

Figure 3D – dose manner

*: Presented photo

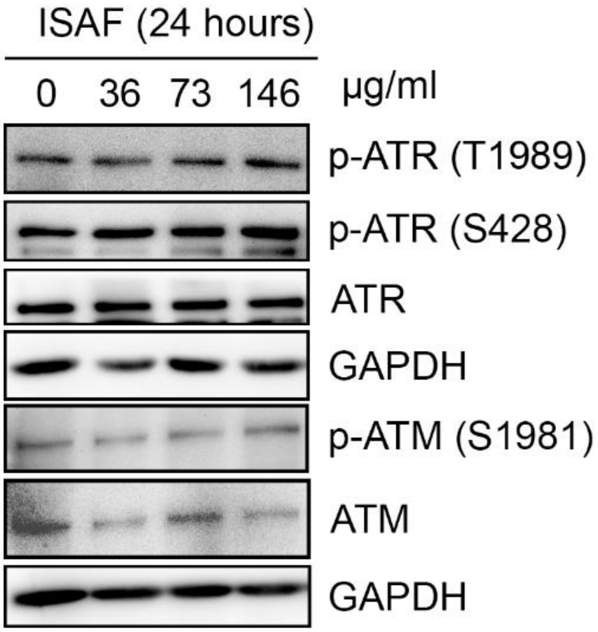

Increasing exposure time

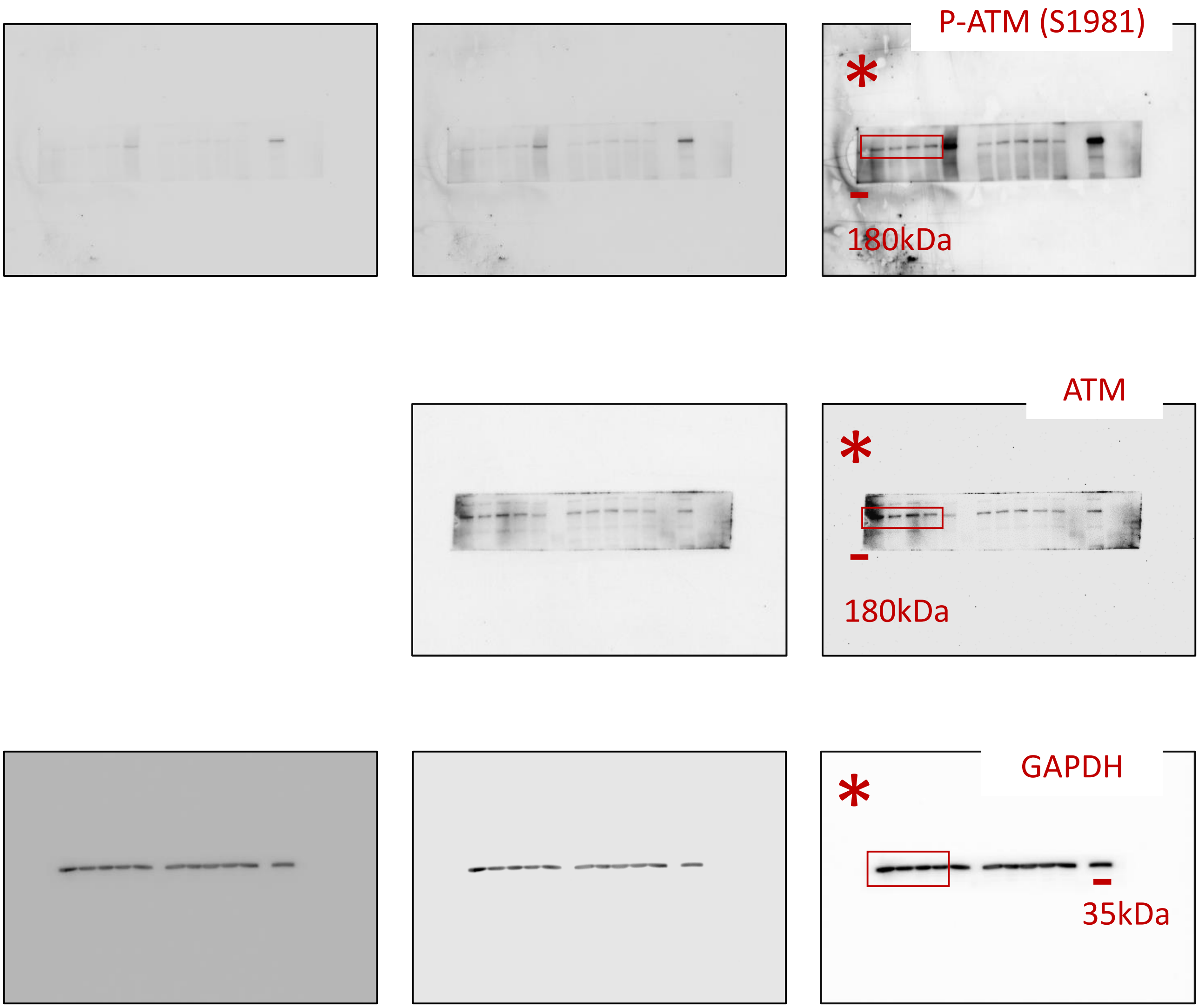

Figure 3D – time manner

*: Presented photo

Increasing exposure time

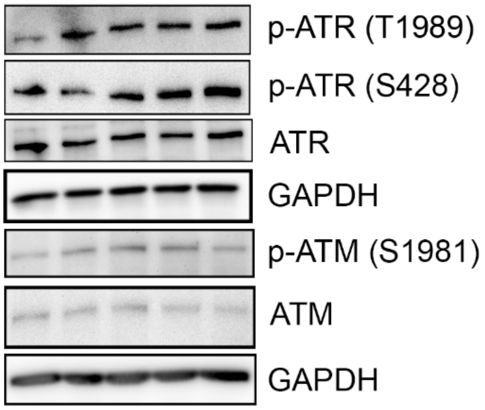

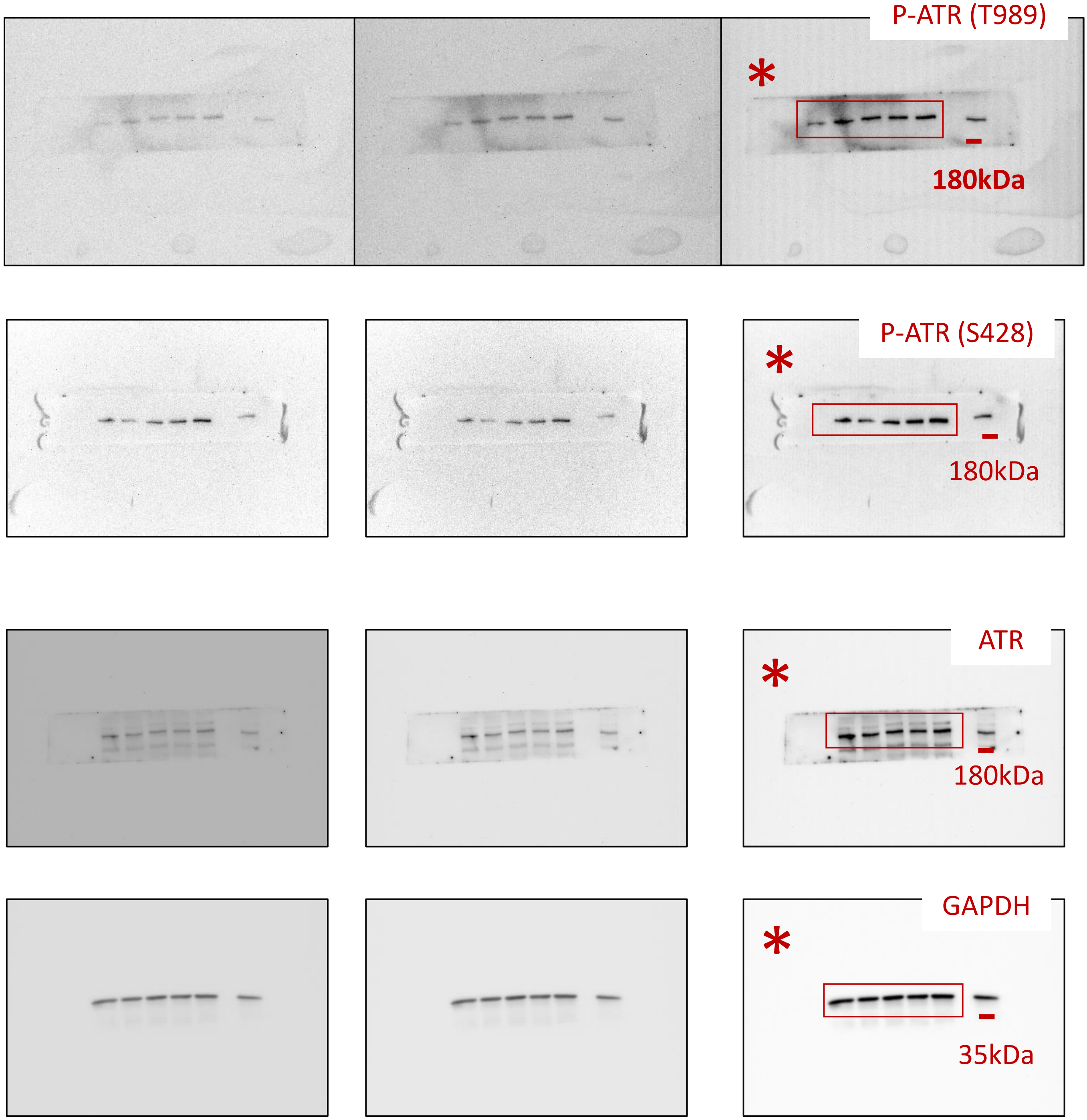

Figure 3D – time manner

*: Presented photo

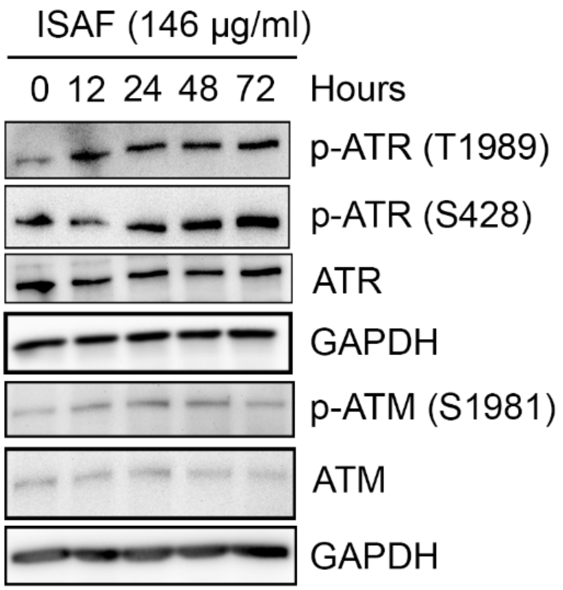

Increasing exposure time

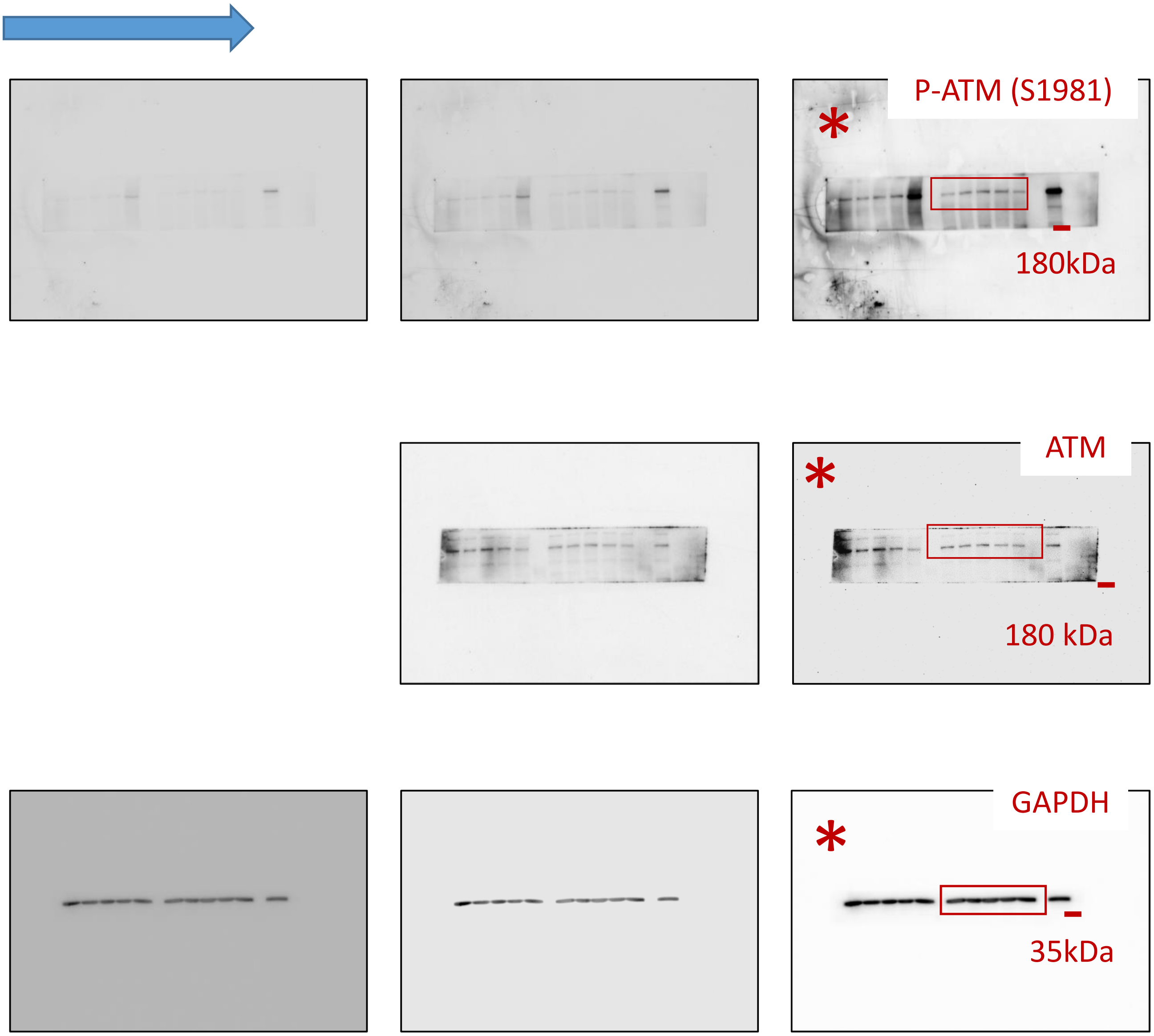

Figure 4C

*: Presented photo

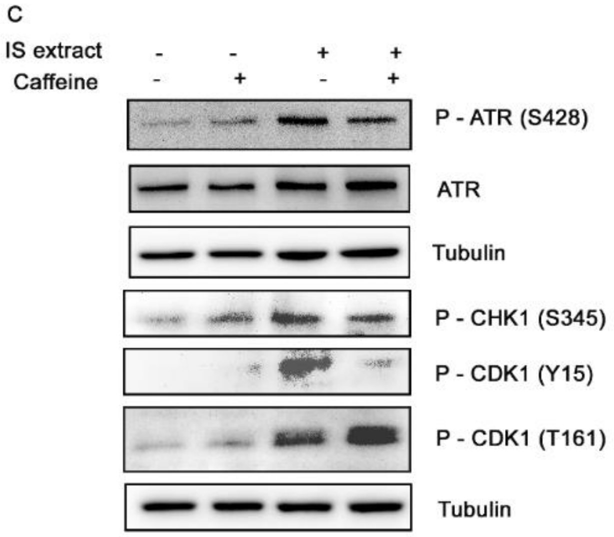

Increasing exposure time

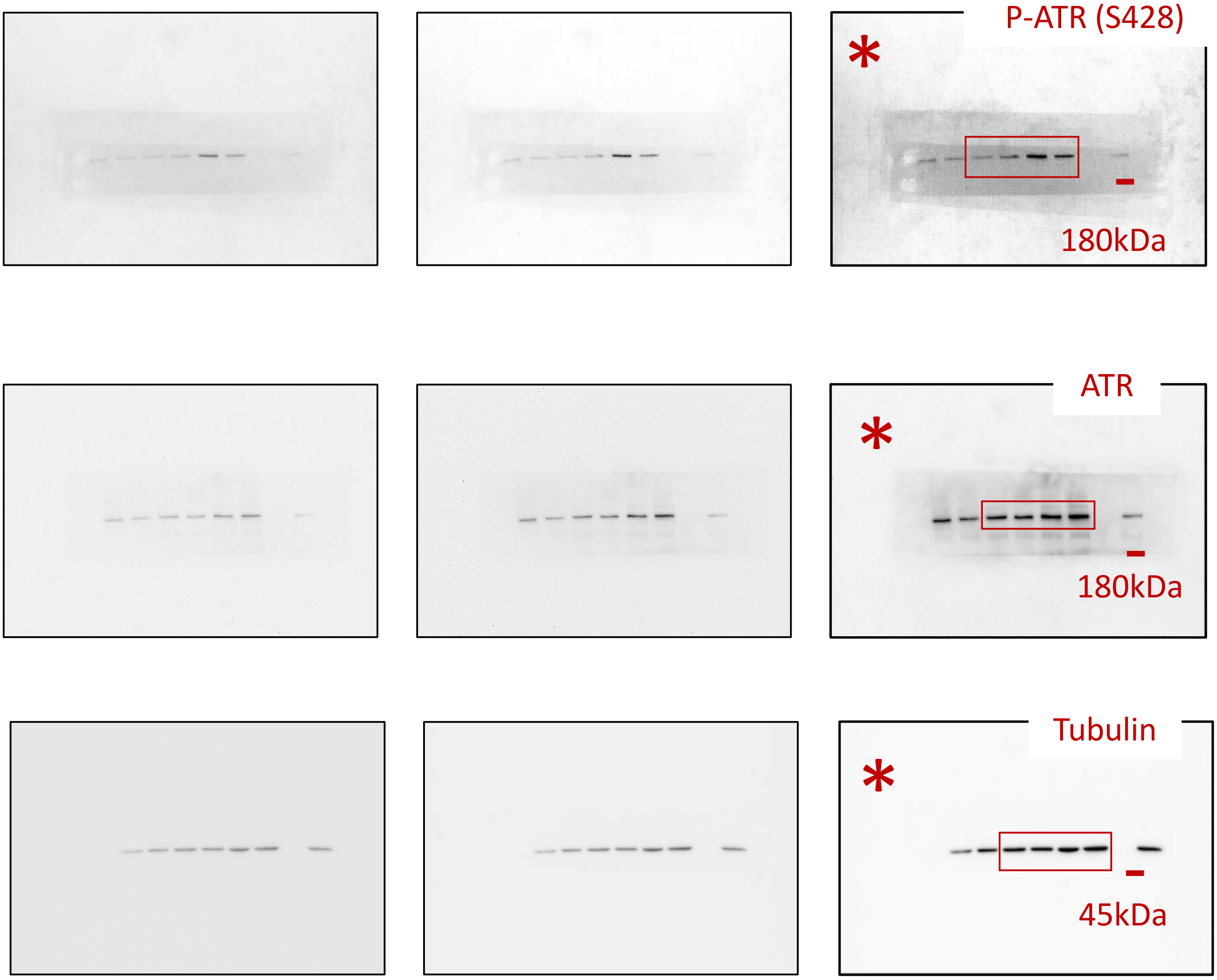

Figure 4C

*: Presented photo

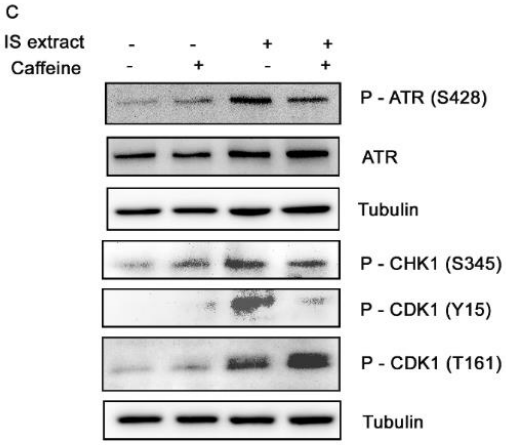

Increasing exposure time

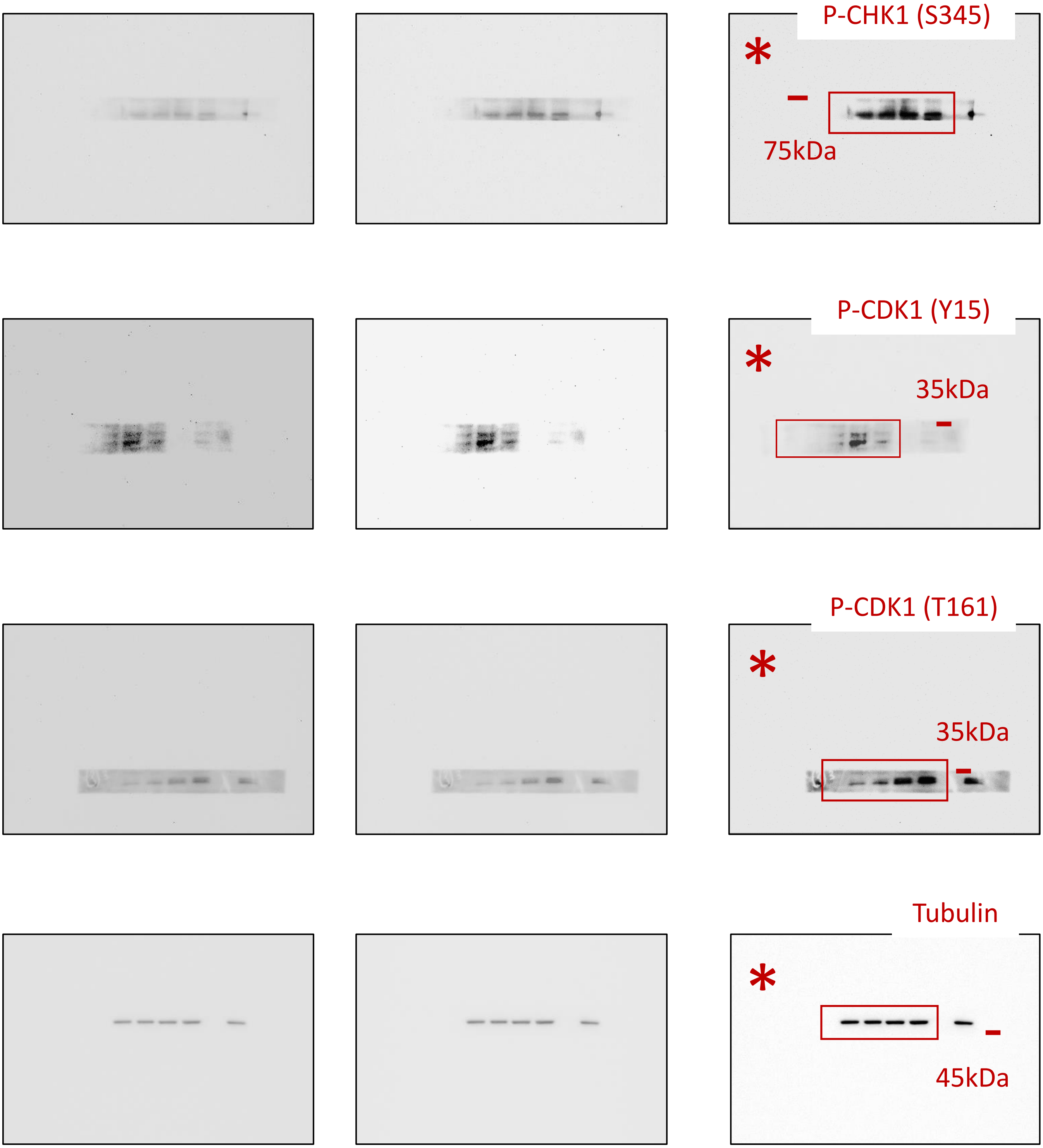

Supplement: Supplementary file 1 — Additional file 1: Supplementary Table S1. Antibodies used in this study. Supplementary Fig. S1. Representative plot of Annexin V staining results. This figure is related to Fig. 2D. Supplementary Fig. S2. The caspase-3/7 activities in Jurkat cells after 12 h of ISAE treatment were assessed by Caspase-Glo® 3/7 assay (Promega) according to the manufacturer's instructions. The graph was expressed as fold changes to control group. Supplementary Fig. S3. Relative quantitative analysis of tryptanthrin, indigo, and indirubin in ISAE. The selected ion current chromatograms of the ISAE extract, tryptanthrin, indigo, and indirubin were carried out through MS full scan experiment in positive mode. The relative abundance of the three selected compounds in ISAE extract were calculated based on comparing the peak area ratios with standard compounds. Supplementary Fig. S4. Cell gating for cell cycle analysis. For cell cycle analysis, cells were gated using forward scatter (FSC) and side scatter (SSC) properties. This helped in excluding cell debris and selecting the desired cells (within the black circle) for analysis. The gating area was established based on the solvent control group (0 μg/mL of ISAE) and was consistently applied to all other groups. This illustration corresponds to Fig. 2A. Original images of western blot. Multiple exposure images of WB. [file 12906_2023_4325_MOESM1_ESM.pdf]
